# Acupuncture for Chronic Low Back Pain in Older Adults: A Randomized Clinical Trial

**DOI:** 10.1001/jamanetworkopen.2025.31348

**Published:** 2025-09-12

**Authors:** Lynn L. DeBar, Robert D. Wellman, Morgan Justice, Andrew L. Avins, Matthew Beyrouty, Carolyn M. Eng, Patricia M. Herman, Arya Nielsen, Alice Pressman, Katie L. Stone, Raymond Y. Teets, Andrea J. Cook

**Affiliations:** 1 Kaiser Permanente Center for Health Research, Portland, Oregon; 2 Kaiser Permanente Washington Health Research Institute, Seattle; 3 Division of Research, Kaiser Permanente Northern California, Oakland; 4 Department of Epidemiology and Biostatistics, University of California, San Francisco; 5 Kaiser Permanente Bernard J. Tyson School of Medicine, Pasadena, California; 6 The Institute for Family Health, New York, New York; 7 Alfred and Gail Engelberg Department of Family Medicine and Community Health, Icahn School of Medicine at Mount Sinai, New York, New York; 8 RAND, Santa Monica, California; 9 California Pacific Medical Center Research Institute, Sutter Health, San Francisco; 10 Center for Health Systems Research, Sutter Health, Walnut Creek, California

## Abstract

**Importance:**

The study was carried out to inform Medicare acupuncture coverage decisions addressing the gap in evidence on acupuncture effectiveness, specifically for older adults with chronic low back pain (CLBP).

**Objective:**

To determine the effectiveness of standard acupuncture (SA) or SA plus maintenance (enhanced acupuncture [EA]) to improve CLBP-related disability relative to usual medical care (UMC) at 3, 6, and 12 months after randomization.

**Design, Setting, and Participants:**

This multisite, 3-arm, parallel-group randomized clinical trial of older adults with CLBP collected data from 4 US health care systems in 3 geographic areas and compared SA and EA treatment with UMC only. Study enrollment was conducted from August 12, 2021, to October 27, 2022; follow-up concluded on November 7, 2023.

**Interventions:**

Both SA (8-15 treatment sessions over 12 weeks plus UMC) and EA (SA plus 4-6 maintenance sessions during the next 12 weeks) were provided by experienced, community-based licensed acupuncturists. Participants were randomized 1:1:1 to the 3 groups.

**Main Outcomes and Measures:**

The primary outcome was CLBP-related disability measured by a baseline-to-6-month change in the Roland-Morris Disability Questionnaire (RMDQ) score. Secondary outcomes included pain intensity and the percentage of participants with clinically meaningful (≥30%) improvements.

**Results:**

The trial identified 800 individuals who were randomized to 3 groups (mean [SD] age, 73.6 [6.0] years; 496 females [62.0%]). At 6 months, RMDQ change scores were significantly better in both the SA and EA groups compared with the UMC only group (SA vs UMC: adjusted mean difference, −1.0 [95% CI, −1.9 to −0.1] and EA vs UMC: adjusted mean difference, −1.5 [95% CI, −2.5 to −0.6]). SA and EA change scores did not differ significantly from one another. The relative benefit of acupuncture compared with UMC on disability persisted at 12 months. Pain intensity exhibited a relative benefit of EA over SA at 6 months, and both acupuncture groups had significant improvement over UMC. The adjusted percentage with clinically meaningful improvements in RMDQ at 6 months was greater for SA (39.1% [95% CI, 33.1%-46.1%]; adjusted relative risk, 1.33 [95% CI, 1.04-1.70]) and for EA (43.8% [95% CI, 38.0%-50.4%]; adjusted relative risk, 1.49 [95% CI, 1.19-1.86]) compared with UMC (29.4% [95% CI, 24.3%-35.5%]) and persisted at 12 months. Rates of serious adverse events were low and similar among groups, with less than 1% that was possibly acupuncture-intervention related.

**Conclusions and Relevance:**

The findings of this randomized clinical trial of older adults with CLBP suggest that acupuncture needling provided greater improvements in back pain–related disability at 6 months and at 12 months compared with UMC alone. These findings support acupuncture needling as an effective and safe treatment option for older adults with CLBP.

**Trial Registration:**

ClinicalTrials.gov Identifier: NCT04982315

## Key Points

**Question:**

Is acupuncture needling (both a standard acupuncture course and additional maintenance sessions) an effective treatment for older adults with chronic low back pain (CLBP)?

**Findings:**

In this randomized clinical trial that included 800 older adults with CLBP, acupuncture needling (both a standard course and additional maintenance sessions) improved pain-related disability with CLBP at 6 months and 12 months, with no statistically discernible benefit of additional maintenance sessions.

**Meaning:**

These findings suggest that acupuncture needling is an effective and safe treatment option for older adults with CLBP.

## Teaser

This randomized clinical trial assesses the effectiveness of acupuncture needling (both a standard course and additional maintenance sessions) for older adults with chronic low back pain.

## Introduction

Low back pain is the leading cause of disability worldwide, with both prevalence and burden increasing with age.^1,2^ Over one-third of US adults aged 65 years or older experience chronic low back pain (CLBP),^3^ with symptoms and disability for many persisting for 1 year or more,^4^ generating costs of over $134 billion annually in the US (with escalating costs in Americans aged ≥65 years).^5^ These large investments have focused primarily on pharmacologic or invasive therapies (back surgery and spinal injections) and have had questionable impact (unproven or modest short-term effects) on the health and functioning of older Americans with CLBP.^6,7,8,9,10,11,12,13^ Older adults have greater prevalence of comorbidities with attendant polypharmacy,^14,15^ and normal age-related physiologic changes place older adults at a substantially increased risk for adverse effects with commonly prescribed CLBP medications including opioids, gabapentinoids, and nonsteroidal anti-inflammatory drugs,^16,17,18,19,20^ making low-risk nonpharmacologic options appealing. Acupuncture has demonstrated effectiveness for CLBP,^21,22,23^ is recommended by the American College of Physicians guidelines as first-line care for treating CLBP,^24,25^ and has an excellent safety profile reported across large studies.^26,27^ Acupuncture also improves sleep and emotional symptoms, which are common concerns among older adults with CLBP.^28,29,30^ However, to our knowledge, no large-scale randomized clinical trials have focused on adults aged 65 years or older, and the optimal dose and timing of acupuncture are unknown for older adults.

In response to a call by the Centers for Medicare & Medicaid Services to inform a national-coverage determination for Medicare reimbursement of acupuncture for CLBP among older adults, a pragmatic randomized clinical trial was designed to address this critical evidence gap. This 3-arm trial compared a standard acupuncture (SA) course (8-15 sessions across 12 weeks plus usual medical care [UMC]) and an enhanced acupuncture (EA) course (SA plus 4-6 additional sessions across the subsequent 12 weeks) with UMC alone for improving CLBP-related disability among adults aged 65 years or older.

## Methods

### Study Design and Setting

The Acupuncture for Chronic Low Back Pain in Older Adults (BackInAction) pragmatic, parallel-group randomized clinical trial was conducted across 4 health care systems in 3 geographic regions (Pacific Northwest: Kaiser Permanente Washington [KPWA], Northern California: Kaiser Permanente Northern California [KPNC] and Sutter Health [SH], and New York City: The Institute for Family Health [IFH]) of different delivery types: integrated-care delivery, fee-for-service, and a Federally Qualified Health Center. Study enrollment was conducted from August 12, 2021, to October 27, 2022; follow-up data collection ended on November 7, 2023. Site-specific recruitment targets were proportional to the health care system’s size. Collectively, these health care systems serve ethnic, racial, and socioeconomic diverse populations.^31,32^ The design, setting, and recruitment have been detailed previously (trial protocol in Supplement 1).^33^ Central ethics approval was provided by the KPNC institutional review board. The trial was monitored by an independent data and safety monitoring board. The research was classified as minimal risk, and all participants provided written, oral, or electronic written consent, according to the local site’s requirements. This report followed Consolidated Standards of Reporting Trials (CONSORT) reporting guideline for parallel-group randomized clinical trials and complies with the Revised Standards for Reporting Interventions in Clinical Trials of Acupuncture (STRICTA).^34^

### Participants

Recruited individuals were aged 65 years or older with nonspecific CLBP (with or without radicular symptoms [sciatica]) persisting for 3 months or longer with pain-related interference (≥3 on the general activity PEG [pain intensity, interference with enjoyment of life, and interference with general activity] item, a 3-item, pain-intensity and pain-related interference measure, in which scores range from 0 to 10 for each of the 3 areas, with higher scores indicating worse impact^35^). Exclusions included vertebral fracture, spinal infection, or active inflammatory disease in the prior year; a current cancer-related diagnosis or serious underlying illness; severe cognitive impairment (dementia, active psychosis, or <3 on cognitive screener)^36^; lower back surgery within the past 3 months; acupuncture within the past 6 months; litigation issues; an inability to speak English (English or Spanish at the IFH site); an inability to attend acupuncture sessions; or being a nursing-home resident or current recipient of hospice or palliative care.

**Figure 1.**
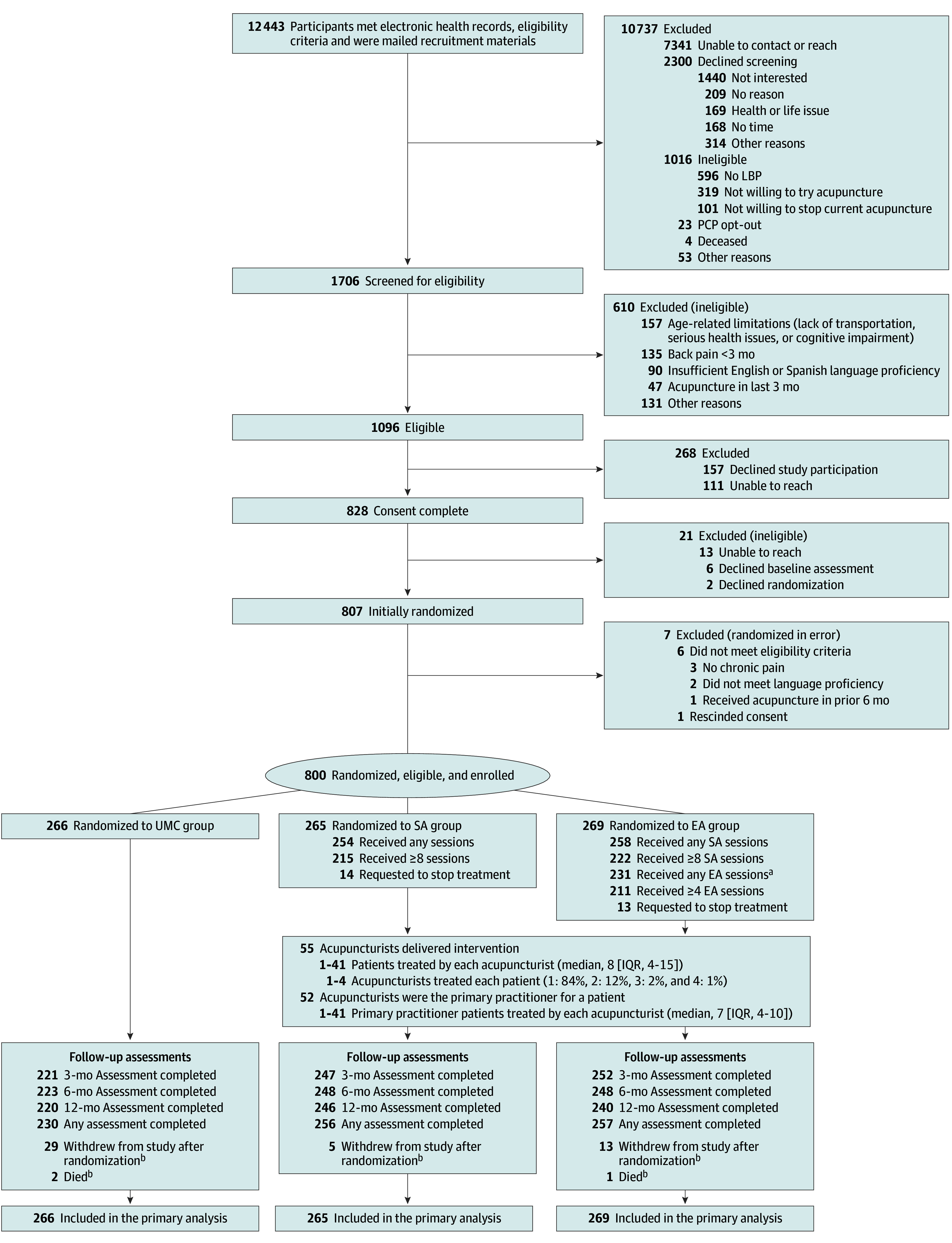
Study Flow Diagram EA indicates standard plus enhanced acupuncture; LBP, low back pain; PCP, primary care provider; SA, standard acupuncture; and UMC, usual medical care. ^a^One participant had 0 SA sessions but attended EA sessions. ^b^Those who withdrew from the study or died may have completed follow-up assessments prior to the event and would consequently be counted as having completed any follow-up.

At KPWA, KPNC, and SH, informational letters were sent to random samples of participating health care system members who met electronic health record (EHR) prescreening criteria. Prescreening was conducted with EHRs, and eligibility was confirmed with interviews. At IFH, most participants were identified by primary care practitioners, and study staff confirmed EHR eligibility. Recruitment and enrollment details are provided in Figure 1, Supplement 1, and elsewhere.^31^

### Randomization and Blinding

After the baseline assessment, participants were randomized 1:1:1 to the 3 groups by research personnel using REDCap software. A study biostatistician (R.D.W.) computer-generated the randomization scheme in R, version 3.6.3 (R Project for Statistical Computing). Participants were assigned to the 3 groups using a random permuted block scheme (block size 3 or 6) that was stratified by site, age (65-74, 75-84, or ≥85 years), and sex. At randomization, research personnel and participants knew only whether participants were randomized to UMC or an acupuncture group but not whether they were randomized to SA or EA, maintaining participants’ blinding to possible later receipt of maintenance acupuncture. Approximately 10 weeks after randomization, all acupuncture-randomized participants and their acupuncturists were informed whether they were randomized to EA by unblinded study personnel who did not conduct follow-up assessments. Baseline interviewers did not conduct any follow-up assessments for any participants who they randomized.

### Interventions

Participants randomized to UMC were asked to avoid acupuncture during the study; those randomized to SA were provided with 15 or fewer treatment sessions (8 sessions were considered a minimum therapeutic dose) plus UMC across 12 weeks, and those randomized to EA received SA plus 6 or fewer additional sessions (4 sessions were considered a minimum therapeutic dose) in the subsequent 12 weeks. As a pragmatic trial, we sought to evaluate acupuncture as delivered in everyday health care settings. More than 50 participating acupuncturists mirrored typical community delivery (ie, licensed acupuncturists [LAcs] practicing independently at KPWA, KPNC, and SH and directly within the clinics at IFH). Importantly, the intervention was restricted to needling only by the funder to align with the Centers for Medicare & Medicaid Services’ expected parameters for Medicare-reimbursable acupuncture treatment, which was under public comment at the time the study was proposed (and has since been approved for coverage). While based in principles of traditional East-Asian acupuncture, the design did not allow other forms of needling (dry needling) or adjuncts such as electroacupuncture, moxibustion, application of heat, *Gua sha*, *Ba guan* cupping, *Tu ina*, or herbal medicine.^37,38^ Based on existing literature and expert consensus, the intervention protocol balanced standardization with flexibility to adapt treatment to individual participant presentations.^38^ The intervention approach is described in detail in the trial protocol (Supplement 1) and elsewhere.^33,38^ All study participants had access to UMC pain management services available in the participating health care systems (eTables 18 and 19 in eAppendix 4 in Supplement 3).

### Follow-Up

Trained interviewers, masked to treatment groups, collected data by telephone at baseline (before randomization) and at 3, 6, and 12 months after randomization. Participants completed the follow-up assessments online or by telephone and were compensated for completing each assessment.^33^

### Measures

Sociodemographic and back-pain information was obtained at baseline. Similarly, baseline measures included measures of frailty^39^ and medical morbidity using the EHR-derived *International Statistical Classification of Diseases and Related Health Problems, Tenth Revision* diagnosis code–based measure, the Elixhauser Comorbidity Index.^40^ Self-reported race and ethnicity categories were Asian, Black, Hispanic, White, and other (American Indian or Alaska Native, Native Hawaiian or Other Pacific Islander, or multiracial) and were included in the study because the funders required this information to track inclusion as well as variation in outcomes by race and ethnicity.

#### Primary Outcome

The primary study outcome was back-pain–related disability as measured by the change in score on the 24-item Roland-Morris Disability Questionnaire (RMDQ) from baseline to 3, 6 (primary time point), and 12 months after randomization.^41^ The RMDQ is a well-validated, patient-reported count of limitations, from 0 to 24, during the past week due to LBP, with higher scores indicating greater functional limitation.

#### Secondary and Post Hoc Pain-Related Outcomes

Secondary measures included the PEG scale^35^ and the proportion of participants with clinically meaningful improvement (≥30% improvement from baseline^42^) on the RMDQ and PEG (including pain intensity) measures. Although extracted from the PEG scale and prespecified as a secondary outcome, pain intensity was inadvertently omitted from the BackInAction clinicaltrials.gov record and, hence, listed here as a post hoc outcome. A final secondary measure was the Patient Global Impression of Change [for Pain] (PGIC),^43^ in which participants rate their improvement in pain on a 7-point scale (much, moderately, or a little worse; no change; a little, moderately, or much better), in which higher scores indicate better pain outcomes.

#### Other Outcomes

We used the validated Patient-Reported Outcomes Measurement Information System (PROMIS) short-form measures^44,45^ to assess physical (T score range from 21 to 59) and social (T score range from 27.5 to 64.2) functioning. Higher scores represent higher functioning; scores less than 40 indicate moderate impairment. Depression was measured using the 2-item Patient Health Questionnaire-2 (scores range from 0 to 6, with higher scores indicating greater severity).^46^ Anxiety was measured using the 2-item Generalized Anxiety Disorder 2-item scale (scores range from 0 to 6, with higher scores indicating greater severity).^47^ Opioid use was ascertained from the EHRs at KPWA, KPNC, and SH only.

#### Adverse Events

Adverse events were identified by acupuncturists, follow-up mailers, and ad hoc participant reports. Serious adverse events, defined as hospitalizations and deaths, were assessed monthly from the EHRs and medical record reviewed by physician monitors (A.L.A. at KPNC and R.Y.T. at IFH) at each site to determine relatedness.

### Sample Size

The sample size of 789 (263 per group) aimed to provide at least 90% power to detect a mean difference of 2 points between each acupuncture group compared with the UMC group (pairwise comparison power) on the primary outcome at 6 months, separate from the 30% or higher improvement from baseline used to reflect a clinically meaningful improvement.^42^ We assumed an SD of 6^48,49,50^ and a missing outcome rate of 20% and controlled for multiple comparisons among study groups using Fisher least significant difference.^51^ Power was calculated with simulation using R software, version 3.6.3.50 (R Project for Statistical Computing).

### Statistical Analysis

Following the prespecified analysis plan (Supplement 2), differences among the 3 groups in the primary outcome (change in RMDQ from baseline) were assessed by fitting a linear regression model that included outcome measures from all 3 follow-up time points (3, 6, and 12 months). Indicators for acupuncture compared with UMC at 3 months (SA and EA are equivalent at 3 months) and interaction terms for the intervention groups (SA and EA) and time points (6 and 12 months) were included in the model to estimate adjusted mean intervention effects, adjusted mean differences (AMDs), and standardized mean differences (SMDs [AMDs divided by SD of outcome change]) between groups at each time point. To control for multiple comparisons, we only compared groups if the omnibus Wald test was statistically significant. Models were fit using generalized estimating equations with an independent working correlation and sandwich SEs to account for within-person and within-practitioner correlation.^52^ We repeated this analysis for secondary and tertiary outcomes but used modified Poisson regression^53^ for binary outcomes to estimate adjusted relative risks (RRs).

To account for potential bias due to missing data, we prespecified adjusting for baseline RMDQ, age, sex, race and ethnicity, and health care system. We further applied missing not-at-random imputation and nonresponse inverse weighting. Additional details are provided in Supplement 2; eAppendix 1 including eTables 1 and 3-9 and the eFigure in Supplement 3; and eTable 2 in Supplement 4.

For the RMDQ primary outcome, we also conducted a prespecified analysis at each time point (6 or 12 months), combining the 2 acupuncture groups; if they were not statistically significant and meaningfully different (>1-point difference), we compared that single group with the UMC group. We also assessed for moderators at 6 months by adding an interaction with the moderator and the combined acupuncture group.

All analyses were intention-to-treat. All tests and CIs were 2-sided, and statistical significance was defined as *P* < .05. All analyses used PC SAS, version 9.4 (SAS Institute Inc) or R, version 4.4 (R Project for Statistical Computing).

## Results

Among 1706 individuals screened, 800 (mean [SD] age, 73.6 [6.0] years; 496 females [62.0%] and 304 males [38.0%]) were eligible, enrolled, and randomized (Figure 1). Among the 534 randomized to both the SA and EA groups, 512 (95.9%) received 1 session or more, and 437 (81.8%) received 8 sessions or more for the SA phase. Of 269 participants randomized to EA, 231 (85.9%) received 1 or more maintenance sessions; 211 (78.4%) received 4 sessions or more. Primary outcome assessment response rates at 6 months were 83.8% for UMC, 93.6% for SA, and 92.2% for EA (Figure 1, with further details in eTable 2 in Supplement 4). STRICTA^34^ details and fidelity to the acupuncture intervention are reported in eTable 20 in eAppendix 5 in Supplement 3.

At baseline, treatment groups were similar except for modest differences in ethnic and racial diversity, income, proportion with high-impact chronic pain, and presence of sciatica (Table 1).^54^ Among the total participants, 328 (41.0%) were aged 75 years or older, and most were women (495 [61.9%]). In terms of self-identified race and ethnicity, 42 participants (5.3%) were Asian, 132 (16.7%) were Black, 86 (10.9%) were Hispanic, 510 (64.6%) were White, and 19 (2.4%) were of other race or ethnicity (of whom 3 [0.4%] were American Indian or Alaska Native, 3 [0.4%] were Native Hawaiian or Other Pacific Islander, and 13 [1.6%] were multiracial). Nearly half of the participants (375 [47.2%]) met criteria for high-impact chronic pain, with most (544 [68.6%]) reporting radicular symptoms (sciatica). For 687 participants (85.9%), CLBP was 1 of multiple musculoskeletal pain conditions, with a mean (SD) 1.7 (1.1) of other pain conditions per participant. The baseline mean (SD) RMDQ (13.2 [5.5]), PEG (5.6 [2.2]), and PROMIS physical functioning (T score, 38.2 [6.6]) scores indicate moderate levels of severity. Finally, less than 5% of participants (24 [3.8%]) received long-term opioids.

**Table 1.** Baseline Participant Characteristics Overall and by Group^a^

Characteristic	Overall (N = 800)	Usual medical care (n = 266)	Acupuncture	
			Standard (n = 265)	Enhanced (n = 269)
**Sociodemographic characteristics**				
Age, mean (SD), y	73.6 (6.0)	73.7 (6.0)	73.4 (5.8)	73.8 (6.1)
Age ≥75 y	328 (41.0)	109 (41.0)	106 (40.0)	113 (42.0)
Sex				
Female^b^	496 (62.0)	168 (63.2)	165 (61.3)	165 (61.3)
Male	304 (38.0)	99 (37.2)	100 (37.7)	105 (39.0)
Race and ethnicity				
Asian	42 (5.3)	14 (5.3)	15 (5.8)	13 (4.9)
Black	132 (16.7)	40 (15.2)	51 (19.7)	41 (15.4)
Hispanic	86 (10.9)	24 (9.1)	34 (13.1)	28 (10.5)
White	510 (64.6)	179 (67.8)	151 (58.3)	180 (67.7)
Other^c^	19 (2.4)	7 (2.7)	8 (3.1)	4 (1.5)
Educational level				
≤High school	107 (13.4)	33 (12.4)	36 (13.6)	38 (14.2)
Some college or vocational school	442 (55.4)	147 (55.3)	154 (58.3)	141 (52.6)
College graduate or higher degree	249 (31.2)	86 (32.3)	74 (28.0)	89 (33.2)
Married or domestic-partnered	455 (57.6)	156 (59.3)	147 (56.1)	152 (57.4)
Annual family income <$50 000	223 (34.7)	77 (36.3)	79 (36.1)	67 (31.6)
**Pain-related characteristics**				
High-impact chronic pain	375 (47.2)	112 (42.3)	125 (47.4)	138 (51.9)
Received disability for pain	77 (9.8)	29 (11.0)	24 (9.2)	24 (9.1)
Radicular symptoms (sciatica)	544 (68.6)	163 (61.7)	186 (71.3)	195 (72.8)
Multiple musculoskeletal pain conditions (per EHR)	687 (85.9)	224 (84.2)	220 (83.0)	243 (90.3)
No. of musculoskeletal pain conditions (per EHR), mean (SD)	1.7 (1.1)	1.6 (1.1)	1.7 (1.2)	1.7 (1.0)
Long-term opioid therapy for pain condition	24 (3.8)	14 (6.8)	7 (3.3)	3 (1.4)
**Other clinical characteristics**				
Medical morbidity (per Elixhauser Comorbidity Index [EHR], mean (SD)	2.5 (2.0)	2.4 (1.9)	2.6 (2.1)	2.5 (2.1)
Met frailty criteria	156 (20.3)	49 (19.1)	58 (22.8)	49 (19.2)
Depression diagnosis	119 (14.9)	40 (15.0)	42 (15.9)	37 (13.8)
Anxiety diagnosis	127 (15.9)	46 (17.3)	35 (13.2)	46 (17.1)
**Baseline measures of the primary and secondary outcome scores**				
Back pain disability (RMDQ [modified]), mean (SD)^d^	13.2 (5.5)	12.9 (5.5)	13.5 (5.4)	13.2 (5.5)
Characteristic pain intensity, mean (SD)^e^	5.8 (2.0)	5.7 (1.9)	6.1 (1.9)	5.8 (2.0)
Composite pain severity (PEG), mean (SD)^f^	5.6 (2.2)	5.4 (2.2)	5.7 (2.1)	5.6 (2.2)
Physical functioning (PROMIS), mean (SD)^g^	38.2 (6.6)	38.3 (6.4)	38.2 (6.5)	38.1 (6.8)
**Baseline measures of other tertiary and ad hoc outcomes**				
Social functioning (PROMIS), mean (SD)^g^	45.5 (8.3)	45.7 (8.2)	45.1 (8.3)	45.6 (8.5)
Depressive threshold symptoms (PHQ-2 [≥3])^h^	162 (20.8)	51 (19.7)	62 (24.0)	49 (18.8)
Anxiety threshold symptoms (GAD-2 subscale of PHQ-4 [≥3])^i^	173 (22.0)	53 (20.2)	63 (24.2)	57 (21.7)

Abbreviations: EHR, electronic health record; GAD-2, General Anxiety Disorder 2-item scale; PEG, pain intensity, interference with enjoyment of life, and interference with general activity; PHQ-2, Patient Health Questionnaire-2; PHQ-4, Personal Health Questionnaire-4; PROMIS, Patient-Reported Outcomes Measurement Information System; RMDQ, Roland-Morris Disability Questionnaire.

^a^ Data are presented as No. (%) of participants unless indicated otherwise. Some values may not sum to the total subsample owing to missing data.

^b^ One person classified as female for stratification purposes self-identified as intersex.

^c^ Includes American Indian or Alaska Native, Native Hawaiian or Other Pacific Islander, and multiracial.

^d^ Scores range from 0 to 24, with higher scores indicating greater functional limitation during the past week due to low back pain.

^e^ A single-item numerical rating scale ranging from 0 to 10 in the past week, in which 10 is the worst pain.

^f^ Scores range from 0 to 10 for each of the 3 areas (pain intensity, interference with enjoyment of life, and interference with general activity), with higher scores indicating worse impact.

^g^ Subscales include physical functioning (T score ranging from 21 to 59) and social functioning (T score ranging from 27.5 to 64.2), with higher scores representing higher functioning (scores <40 indicate moderate impairment).

^h^ Scores range from 0 to 6, with higher scores indicating greater severity.

^i^ Scores range from 0 to 6, with higher scores indicating greater severity.

### Primary and Secondary Pain-Related Outcomes

**Figure 2.**
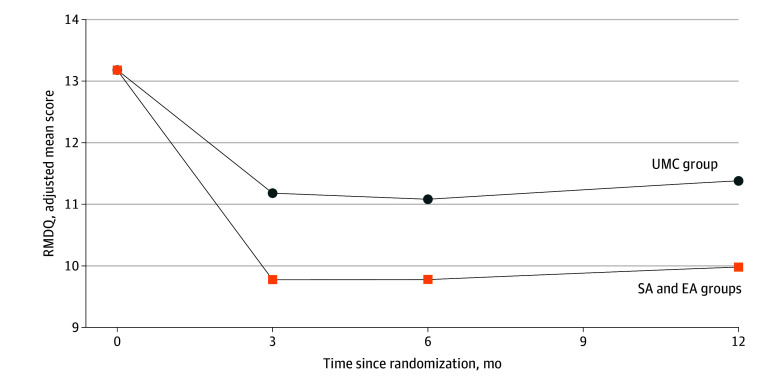
Functional Disability: Acupuncture vs Usual Medical Care (UMC) At 3 months, the adjusted mean difference (AMD) was –1.4 (95% CI, –2.1 to –0.7); the standardized mean difference (SMD) was –0.32. At 6 months, the AMD was −1.3 (95% CI, −2.0 to −0.5); the SMD was −0.27. At 12 months, the AMD was −1.4 (95% CI, −2.2 to −0.6); the SMD was −0.27. EA indicates standard plus enhanced acupuncture; RMDQ, Roland-Morris Disability Questionnaire; and SA, standard acupuncture.

At the 6-month primary time point, there were significantly larger reductions in RMDQ scores in both acupuncture groups compared with usual care (SA vs UMC: AMD, −1.0 [95% CI, −1.9 to −0.1] and SMD, −0.21; EA vs UMC: AMD, −1.5 [95% CI, −2.5 to −0.6] and SMD, −0.32), but SA and EA did not differ significantly (AMD, −0.5 [95% CI, −1.5 to 0.5] and SMD, −0.11) (Table 2). Findings were similar at 12 months. When comparing the acupuncture-combined group with the UMC group, there were statistically significant differences at 3 months (AMD, −1.4 [95% CI, −2.1 to −0.7] and SMD, −0.32), 6 months (AMD, −1.3 [95% CI, −2.0 to −0.5] and SMD, −0.27), and 12 months (AMD, −1.4 [95% CI, −2.2 to −0.6] and SMD, −0.27) (Figure 2). Results were consistent across missing data sensitivity analyses (Supplement 3 eAppendix 2 including eTables 10-14 and eAppendix 3 eTable 17). There were no statistically significant moderators of the 6-month effect between acupuncture groups and UMC alone (eTables 15 and 16 in eAppendix 3 in Supplement 3). Results were consistent across missing data sensitivity analyses (eTables 10-13 in eAppendix 2 and eTables 14 and 17 in eAppendix 3 in Supplement 3).

**Table 2.** Adjusted Mean Changes From Baseline by Group and Pairwise AMDs Between Groups for the Primary Outcome (RMDQ) and All Continuous Secondary Outcomes

Outcome	Adjusted mean change from baseline (95% CI)			Omnibus *P* value^a^	Pairwise AMD (95% CI)			SMD^b^		
	UMC	SA	EA		SA vs UMC	EA vs UMC	EA vs SA			
**Back-related dysfunction (RMDQ)**										
3 mo	−2.0 (−2.6 to −1.4)	−3.4 (−3.8 to −3.0)	NA	<.001	−1.4 (−2.1 to −0.7)	NA	NA	−0.32	NA	NA
6 mo	−2.1 (−2.7 to −1.5)	−3.1 (−3.7 to −2.5)	−3.6 (−4.3 to −3.0)	.002	−1.0 (−1.9 to −0.1)	−1.5 (−2.5 to −0.6)	−0.5 (−1.5 to 0.5)	−0.21	−0.32	−0.11
12 mo	−1.8 (−2.5 to −1.1)	−3.0 (−3.5 to −2.5)	−3.5 (−4.2 to −2.8)	.002	−1.2 (−2.1 to −0.3)	−1.7 (−2.6 to −0.7)	−0.5 (−1.4 to 0.4)	−0.22	−0.31	−0.09
**PEG**										
3 mo	−0.6 (−0.8 to −0.4)	−1.4 (−1.5 to −1.3)	NA	<.001	−0.8 (−1.1 to −0.5)	NA	NA	−0.43	NA	NA
6 mo	−0.6 (−0.9 to −0.3)	−1.1 (−1.3 to −0.8)	−1.5 (−1.8 to −1.3)	<.001	−0.4 (−0.8 to −0.1)	−0.9 (−1.3 to −0.6)	−0.5 (−0.9 to −0.1)	−0.22	−0.45	−0.24
12 mo	−0.7 (−0.9 to −0.4)	−1.1 (−1.3 to −0.9)	−1.3 (−1.5 to −1.1)	.001	−0.4 (−0.8 to −0.1)	−0.6 (−1.0 to −0.3)	−0.2 (−0.6 to 0.1)	−0.21	−0.31	−0.10
**Pain intensity (P of PEG)**										
3 mo	−0.6 (−0.9 to −0.4)	−1.5 (−1.7 to −1.4)	NA	<.001	−0.9 (−1.2 to −0.6)	NA	NA	−0.52	NA	NA
6 mo	−0.6 (−0.8 to −0.3)	−1.1 (−1.3 to −0.9)	−1.6 (−1.8 to −1.3)	<.001	−0.5 (−0.8 to −0.2)	−1.0 (−1.3 to −0.6)	−0.5 (−0.8 to −0.1)	−0.27	−0.51	−0.24
12 mo	−0.8 (−1.1 to −0.6)	−1.2 (−1.4 to −1.0)	−1.3 (−1.6 to −1.1)	.005	−0.4 (−0.7 to 0)	−0.5 (−0.9 to −0.2)	−0.1 (−0.5 to 0.2)	−0.19	−0.27	−0.07
**PROMIS physical function subscale**										
3 mo	0.8 (0.1 to 1.4)	1.6 (1.2 to 2.1)	NA	.03	0.9 (0.1 to 1.7)	NA	NA	0.19	NA	NA
6 mo	1.0 (0.4 to 1.7)	1.2 (0.6 to 1.8)	1.8 (1.2 to 2.5)	.24	0.2 (−0.8 to 1.1)	0.8 (−0.1 to 1.7)	0.6 (−0.4 to 1.6)	0.03	0.16	0.13
12 mo	1.0 (0.3 to 1.7)	1.7 (1.0 to 2.3)	1.5 (0.9 to 2.0)	.35	0.7 (−0.3 to 1.6)	0.5 (−0.4 to 1.4)	−0.2 (−1.0 to 0.7)	0.13	0.09	−0.03
**Patient global impression of change–pain**										
3 mo	3.4 (3.2 to 3.5)	4.5 (4.4 to 4.6)	NA	<.001	1.2 (0.9 to 1.4)	NA	NA	0.86	NA	NA
6 mo	3.3 (3.1 to 3.5)	4.0 (3.8 to 4.1)	4.6 (4.4 to 4.7)	<.001	0.7 (0.4 to 1.0)	1.3 (1.0 to 1.5)	0.6 (0.3 to 0.9)	0.48	0.89	0.41
12 mo	3.3 (3.1 to 3.6)	3.9 (3.7 to 4.1)	4.1 (4.0 to 4.3)	<.001	0.5 (0.2 to 0.8)	0.8 (0.5 to 1.1)	0.3 (0 to 0.5)	0.32	0.49	0.16
**Patient global impression of change–general**										
3 mo	3.4 (3.3 to 3.6)	4.4 (4.3 to 4.6)	NA	<.001	1.0 (0.8 to 1.2)	NA	NA	0.80	NA	NA
6 mo	3.4 (3.2 to 3.6)	4.0 (3.9 to 4.1)	4.5 (4.3 to 4.6)	<.001	0.6 (0.4 to 0.8)	1.1 (0.8 to 1.3)	0.5 (0.2 to 0.7)	0.41	0.74	0.33
12 mo	3.4 (3.2 to 3.6)	3.9 (3.7 to 4.1)	4.1 (3.9 to 4.3)	<.001	0.5 (0.2 to 0.8)	0.7 (0.4 to 1.0)	0.2 (0 to 0.5)	0.29	0.45	0.16
**PROMIS social functioning subscale**										
3 mo	0.4 (−0.5 to 1.2)	1.6 (1.0 to 2.2)	NA	.02	1.2 (0.2 to 2.3)	NA	NA	0.18	NA	NA
6 mo	0.5 (−0.4 to 1.4)	1.3 (0.5 to 2.1)	1.6 (0.5 to 2.6)	.22	0.8 (−0.4 to 2.0)	1.1 (−0.2 to 2.5)	0.3 (−1.0 to 1.7)	0.12	0.16	0.05
12 mo	−0.2 (−1.0 to 0.6)	1.6 (0.9 to 2.3)	1.4 (0.6 to 2.1)	.004	1.8 (0.7 to 2.9)	1.6 (0.4 to 2.7)	−0.2 (−1.2 to 0.8)	0.25	0.22	−0.03
**Depression screener (PHQ-2)**										
3 mo	0 (−0.2 to 0.1)	−0.2 (−0.3 to −0.1)	NA	.19	−0.1 (−0.3 to 0.1)	NA	NA	−0.10	NA	NA
6 mo	0 (−0.2 to 0.2)	−0.2 (−0.4 to −0.1)	−0.3 (−0.4 to −0.1)	.047	−0.3 (−0.5 to 0)	−0.3 (−0.5 to 0)	0 (−0.2 to 0.2)	−0.16	−0.18	−0.02
12 mo	−0.2 (−0.4 to −0.1)	−0.4 (−0.5 to −0.3)	−0.3 (−0.4 to −0.2)	.30	−0.2 (−0.4 to 0.1)	0 (−0.2 to 0.1)	0.1 (−0.1 to 0.3)	−0.10	−0.03	0.07
**Anxiety screener (GAD-2)**										
3 mo	0 (−0.1 to 0.2)	−0.2 (−0.3 to 0)	NA	.07	−0.2 (−0.4 to 0)	NA	NA	−0.13	NA	NA
6 mo	0.1 (−0.2 to 0.3)	−0.3 (−0.4 to −0.1)	−0.2 (−0.4 to 0)	.03	−0.3 (−0.6 to −0.1)	−0.3 (−0.5 to 0)	0.1 (−0.2 to 0.3)	−0.19	−0.16	0.04
12 mo	0 (−0.2 to 0.2)	−0.4 (−0.5 to −0.3)	−0.3 (−0.4 to −0.1)	.006	−0.4 (−0.6 to −0.2)	−0.3 (−0.5 to 0)	0.1 (−0.1 to 0.3)	−0.23	−0.17	0.06

Abbreviations: AMD, adjusted mean difference; EA, enhanced acupuncture; GAD-2, General Anxiety Disorder 2-item subscale; NA, not applicable; PEG, pain intensity, interference with enjoyment of life, and interference with general activity; PHQ-2, Patient Health Questionnaire-2; PROMIS, Patient-Reported Outcomes Measurement Information System; RMDQ, Roland-Morris Disability Questionnaire; SA, standard acupuncture; SMD, standardized mean difference; UMC, usual medical care.

^a^ To account for multiple comparisons, pairwise inference was only performed if the omnibus *P* value was significant.

^b^ Defined as the adjusted mean difference divided by the SD of the change in the outcome.

While patterns were similar across secondary pain-related outcomes, both also showed a relative benefit of EA over SA at the 6-month primary time point (PEG AMD, −0.5 [95% CI, −0.9 to −0.1] and PEG-derived pain intensity rating AMD, −0.5 [95% CI, −0.8 to −0.1]). PGIC for pain showed similar benefit for EA over SA at the 6-month primary time point (AMD, 0.6 [95% CI, 0.3-0.9]). For clinically meaningful improvement analyses of pain-related disability measured by the RMDQ (Table 3), the adjusted percentage at 6 months was greater for SA (39.1% [95% CI, 33.1%-46.1%]; RR, 1.33 [95% CI, 1.04-1.70]) and EA (43.8% [95% CI, 38.0%-50.4%]; RR, 1.49 [95% CI, 1.19-1.86) than for UMC (29.4% [95% CI, 24.3%-35.5%]); this persisted at 12 months.

**Table 3.** Adjusted Percentages of Outcomes by Group and Adjusted Relative Risk Pairwise Comparisons Between Groups for All Binary Secondary Outcomes

Outcome	Adjusted % (95% CI)			Omnibus *P* value^a^	Adjusted relative risk (95% CI)		
	UMC	SA	EA		SA vs UMC	EA vs UMC	EA vs SA
**RMDQ, 30% reduction from baseline^b^**							
3 mo	29.9 (24.9-36.0)	42.0 (38.0-46.4)	42.0 (38.0-46.4)	<.001	1.40 (1.15-1.72)	NA	NA
6 mo	29.4 (24.3-35.5)	39.1 (33.1-46.1)	43.8 (38.0-50.4)	.002	1.33 (1.04-1.70)	1.49 (1.19-1.86)	1.12 (0.88-1.43)
12 mo	28.4 (23.4-34.4)	37.7 (33.6-42.3)	43.8 (39.0-49.2)	<.001	1.33 (1.06-1.66)	1.54 (1.25-1.91)	1.16 (0.98-1.37)
**PEG, 30% reduction from baseline**							
3 mo	23.3 (18.7-29.1)	41.1 (37.6-44.9)	41.1 (37.6-44.9)	<.001	1.76 (1.39-2.24)	NA	NA
6 mo	29.4 (24.4-35.5)	35.0 (30.5-40.2)	40.5 (35.6-46.1)	.02	1.19 (0.94-1.50)	1.38 (1.10-1.73)	1.16 (0.94-1.42)
12 mo	29.5 (24.5-35.5)	35.9 (31.3-41.0)	35.8 (30.4-42.2)	.18	1.22 (0.97-1.52)	1.21 (0.95-1.55)	1.00 (0.80-1.25)
**Pain intensity, 30% reduction from baseline**							
3 mo	22.3 (17.6-28.1)	43.3 (39.3-47.7)	43.3 (39.3-47.7)	<.001	1.95 (1.52-2.49)	NA	NA
6 mo	24.2 (19.4-30.2)	34.8 (28.7-42.2)	41.3 (35.5-48.0)	<.001	1.44 (1.07-1.93)	1.71 (1.31-2.22)	1.19 (0.90-1.56)
12 mo	27.8 (22.8-33.9)	35.0 (30.0-40.8)	35.6 (30.2-41.9)	.10	1.26 (0.98-1.61)	1.28 (1.00-1.64)	1.02 (0.80-1.29)
**Patient global impression of change in pain, much or moderately better**							
3 mo	18.5 (14.0-24.3)	50.2 (45.0-56.0)	50.2 (45.0-56.0)	<.001	2.72 (2.04-3.64)	NA	NA
6 mo	19.5 (15.0-25.4)	37.5 (32.6-43.2)	54.5 (48.0-61.9)	<.001	1.92 (1.43-2.59)	2.80 (2.10-3.73)	1.45 (1.22-1.74)
12 mo	24.2 (19.2-30.3)	35.6 (30.9-41.0)	43.3 (38.0-49.3)	<.001	1.47 (1.13-1.92)	1.79 (1.39-2.32)	1.22 (0.99-1.50)
**Patient global impression of change in general, much or moderately better**							
3 mo	17.8 (13.6-23.5)	49.8 (44.4-55.9)	49.8 (44.4-55.9)	<.001	2.79 (2.08-3.75)	NA	NA
6 mo	22.6 (17.9-28.7)	36.3 (32.6-40.4)	51.8 (46.3-57.8)	<.001	1.60 (1.24-2.07)	2.29 (1.77-2.95)	1.43 (1.23-1.65)
12 mo	24.6 (19.8-30.5)	36.4 (31.3-42.4)	39.9 (33.7-47.2)	.002	1.48 (1.13-1.94)	1.62 (1.24-2.13)	1.10 (0.87-1.38)
**PHQ-2 score ≥3**							
3 mo	11.8 (9.0-15.4)	8.6 (6.6-11.3)	8.6 (6.6-11.3)	.048	0.73 (0.53-1.00)	NA	NA
6 mo	12.8 (10.0-16.4)	8.5 (6.8-10.8)	7.9 (5.7-10.8)	.02	0.67 (0.49-0.92)	0.61 (0.42-0.89)	0.92 (0.65-1.29)
12 mo	8.8 (6.4-12.1)	7.0 (5.4-9.1)	7.8 (5.5-11.2)	.47	0.79 (0.54-1.15)	0.88 (0.55-1.42)	1.12 (0.72-1.75)
**GAD-2 score ≥3**							
3 mo	15.8 (12.4-20.1)	12.2 (10.1-14.7)	12.2 (10.1-14.7)	.06	0.77 (0.59-1.02)	NA	NA
6 mo	17.4 (13.7-22.2)	10.7 (8.3-13.8)	10.9 (8.5-14.1)	.003	0.61 (0.44-0.86)	0.63 (0.45-0.87)	1.02 (0.71-1.48)
12 mo	16.2 (12.5-20.9)	9.5 (7.5-12.1)	9.4 (6.9-12.9)	.006	0.59 (0.42-0.82)	0.58 (0.39-0.87)	0.99 (0.74-1.33)

Abbreviations: EA, enhanced acupuncture; GAD-2, General Anxiety Disorder 2-item scale; NA, not applicable; PEG, pain intensity, interference with enjoyment of life, and interference with general activity; PHQ-2, Patient Health Questionnaire-2; RMDQ, Roland-Morris Disability Questionnaire; SA, standard acupuncture; UMC, usual medical care.

^a^ To account for multiple comparisons, pairwise inference was only performed if the omnibus *P* value was significant.

^b^ The unadjusted percentage of participants with 30% improvement in RMDQ is presented in eTable 17 in eAppendix 3 in Supplement 3.

### Other Secondary and Tertiary Outcomes

There were few significant differences in other outcomes among SA, EA, and UMC (Table 3). The acupuncture-combined group had significantly more improvement in physical and social-role functioning than the UMC group at 3 months. Patients reported significant reductions in anxiety symptoms in both acupuncture groups relative to usual care at 6 months and 12 months; the 2 acupuncture groups did not differ from one another.

### Adverse Events

Rates for serious adverse events were similar across all study groups (hospitalizations: 25 [9.4%] in SA, 23 [8.6%] in EA, and 18 [6.8%] in UMC; deaths: <5, with 0 identified as related or possibly related to the intervention). Only 1 (<1%) hospitalization serious adverse event (lower extremity cellulitis) was adjudicated as possibly related to the study intervention, which was treated successfully with intravenous antibiotics. Potentially treatment-related nonserious adverse events were reported only in the acupuncture groups. There were 71 minor adverse events reported among 52 of the 534 acupuncture-allocated participants (9.7%) with most related (29 [40.8%]) or possibly related (21 [29.6%]) to the intervention. The most common treatment-related adverse events were pain or discomfort at needling sites.

## Discussion

This randomized clinical trial found that among older adults with CLBP, acupuncture needling plus UMC, compared with UMC alone, resulted in greater improvement in CLBP-related dysfunction at 6 months’ follow-up (primary time point) with the modest benefit largely sustained at 12 months. While a 2-point RMDQ difference was used to determine sample-size power, 30% or more improvement from baseline was used as the threshold for clinically meaningful improvement.^42^ Our resulting 1.0- to 1.5-point RMDQ difference is clinically important, congruent with or larger than effects reported for other pain-related treatments,^55,56^ and shows more sustained benefit and substantially lower adverse effects than found for pharmacotherapy, the most prevalent pain-management strategy for older adults with CLBP.^7,9,13^

There were no statistically significant differences in the primary outcome between SA and EA groups. Similar findings were observed with other pain outcomes (PEG [pain intensity] and PGIC) and for those meeting clinically meaningful improvement thresholds for these outcomes. With the exception of a reduction in anxiety symptoms, other secondary outcomes (social and physical functioning and depression) did not suggest an advantage for acupuncture over UMC. Other notable findings include a high level of adherence to acupuncture, with more than 80% reaching critical dose (≥8 sessions) for the SA phase. That we were readily able to enroll a large sample of older adults for in-person treatment despite heightened risks for this population due to the COVID-19 pandemic during the study period further suggests treatment interest and acceptability.

Several factors beyond the magnitude of group differences should be considered when interpreting the clinical importance of these findings. The pattern and magnitude of benefit in this study were comparable with previous acupuncture trials and other evidence-based treatments recommended for CLBP.^22,55,56^ Importantly, minor and serious adverse event rates were low; rates and types of adverse events were similar to acupuncture studies among general adult populations.^27^ As acupuncture has been found to be more effective and safer than medications for LBP,^11,57^ and, given relatively high rates and potential adverse consequences of polypharmacy among older adults,^14,15^ acupuncture may become an important first-line treatment for CLBP in this population.

### Strengths and Limitations

This study’s strengths include a large and geographic diverse sample recruited from multiple health care settings in which racial and ethnic diversity aligned with recent US census estimates, suggesting generalizability of the findings.^58^ Furthermore, the more than 50 licensed acupuncturists who provided the intervention were drawn from those practicing independently in community settings, enhancing the pragmatic nature of the trial. Flexibility for tailoring the needling protocol to individual patient needs also better reflects services in routine clinical care settings than a more rigid needling protocol would.^38^

This study also has limitations. As a pragmatic comparative-effectiveness randomized clinical trial, we used a usual-care comparator, as it was most pertinent to evaluate the added benefit of acupuncture in routine clinical care settings^59,60,61,62^ and purposely focused on patient self-reported pain and pain-related interference as commonly used metrics in frontline clinical care. We did not use a sham control, as it would not have been appropriate to evaluate our central study question on the potential benefit of acupuncture over the availability of usual pain-related medical care services and because of concerns that using sham comparators may underestimate the actual clinical benefit of acupuncture.^59,63^ Yet, such an approach did not allow us to tease out the effect of attention or other nonspecific effects on the outcomes nor the subjectivity of patient self-report on the results. Other limitations included our inability to evaluate the treatment impact on medication changes due to limited availability of medication-dispensing data in 2 of our clinical settings. Furthermore, while we attempted to correct for potential missing-outcome bias with imputation and nonresponse weighting, potential bias may remain, especially given the differential loss to follow-up by groups. Because of the large number of comparisons, significant findings for secondary outcomes should be interpreted cautiously.

Finally, although our favorable findings support the case for enabling broad availability of acupuncture for first-line treatment of CLBP in older adults, licensed acupuncturists, who provide the majority of US acupuncture services, are currently restricted from billing Medicare without a supervising Medicare-approved clinician.^37,64^ Reducing such barriers could vastly improve access to acupuncture for older adults with CLBP.

## Conclusions

In this randomized clinical trial of older adults with CLBP, acupuncture needling provided greater improvements in CLBP-related dysfunction at a 6-month and 12-month follow-up compared with UMC alone with the advantage of a low-risk profile. These findings support acupuncture needling as an effective and safe treatment option for older adults with CLBP.

## Notes

Supplement 1.
Trial Protocol

Supplement 2.
Statistical Analysis Plan

Supplement 3. **eAppendix 1.** Missing Data **eTable 1.** Primary Outcome Follow-Up Rates and Patterns Overall and By Arm **eTable 3.** Baseline Variables Used in Process of Selecting Covariates Related to Missingness and to the 6-Month RDMQ **eTable 4.** Imputation Step 1: Summary of Fitted Imputation Model for Primary Analysis **eTable 5.** Imputation Step 2: Summary of Primary Estimation Model Fit to Imputed Data With Weighting **eTable 6.** Imputation Step 2: Summary of Estimation Model With Combined Acupuncture Groups With Weighting **eTable 7.** Summary of Logistic Weighting Model for Any Observed Follow-Up **eTable 8.** Summary of Estimated Weights **eFigure.** Histogram of Estimated Probabilities and Weights **eTable 9.** Summary of Step 2 Estimation Model Fit on Imputed Data Without Nonresponse Weights **eAppendix 2.** Sensitivity Analyses **eTable 10.** Primary Outcome Results Using Imputed Data Without Weights **eTable 11.** Complete Case Analysis of Primary Outcome Adjusting for Baseline Age, Sex, Race and Ethnicity, HCS, and RMDQ **eTable 12.** Complete Case Analysis of Primary Outcome Adjusting for Baseline Age, Sex, Race and Ethnicity, HCS, Education, RMDQ, PEG, Substance Use Disorder, Fear Avoidance, General Pain, and Pain Catastrophizing **eTable 13.** Complete Case Analysis of Primary Outcome Weighted for Nonresponse and Adjusting for Baseline Age, Sex, Race and Ethnicity, HCS, Education, RDMQ, PEG, Substance Use Disorder, Fear Avoidance, General Pain, and Pain Catastrophizing **eAppendix 3.** Secondary and Exploratory Analyses **eTable 14.** Adjusted Mean Changes from Baseline by Combined Acupuncture Arm and Adjusted Mean Differences in Change Between Combined Acupuncture Arm Versus UMC for the Primary Outcome (RMDQ) **eTable 15.** Moderator Analysis of the Effect of Acupuncture Versus Usual Medical Care (UMC) on Change in RMDQ at 6 Months From Baseline **eTable 16.** Moderator Analysis of the Effect of Acupuncture Versus Usual Medical Care (UMC) by Baseline RMDQ on 30% Reduction in RMDQ at 6 Months From Baseline **eTable 17.** Unadjusted Percentage of Participants With 30% Improvement in RMDQ eAppendix 4. Usual Medical Care Services and Fidelity **eTable 18.** Health Care System Provided Health Insurance Covered/Partially Covered Pain-Related Usual Medical Care **eTable 19.** Back Pain-Related Health Care Utilization by Study Group eAppendix 5. STRICTA CONSORT Extensions (Standards for Reporting Interventions in Clinical Trials of Acupuncture) **eTable 20.** Fidelity to STRICTA Checklist for Reporting Interventions in a Clinical Trial of Acupuncture
eReferences

Supplement 4. **eTable 2.** Baseline Characteristics Overall and by Category of Data Primary Outcome Completion: Missing All 3 Follow-Up, Missing 1 or 2 Follow-Ups, or Having All Follow-Ups Observed

Supplement 5.
Data Sharing Statement

## References

[1] GBD 2021 Low Back Pain Collaborators. Global, regional, and national burden of low back pain, 1990-2020, its attributable risk factors, and projections to 2050: a systematic analysis of the Global Burden of Disease Study 2021. Lancet Rheumatol. 2023;5(6):e316-e329. doi:10.1016/S2665-9913(23)00098-X 37273833 PMC10234592

[2] de Souza IMB, Sakaguchi TF, Yuan SLK, . Prevalence of low back pain in the elderly population: a systematic review. Clinics (Sao Paulo). 2019;74:e789. doi:10.6061/clinics/2019/e789 31664424 PMC6807687

[3] Wong CK, Mak RY, Kwok TS, . Prevalence, incidence, and factors associated with non-specific chronic low back pain in community-dwelling older adults aged 60 years and older: a systematic review and meta-analysis. J Pain. 2022;23(4):509-534. doi:10.1016/j.jpain.2021.07.012 34450274

[4] Rundell SD, Sherman KJ, Heagerty PJ, Mock CN, Jarvik JG. The clinical course of pain and function in older adults with a new primary care visit for back pain. J Am Geriatr Soc. 2015;63(3):524-530. doi:10.1111/jgs.13241 25754841

[5] Dieleman JL, Cao J, Chapin A, . US health care spending by payer and health condition, 1996-2016. JAMA. 2020;323(9):863-884. doi:10.1001/jama.2020.0734 32125402 PMC7054840

[6] Mitchell JM, Hadley J. Treatments and health outcomes of Medicare patients with back pain. Med Care Res Rev. 2020;77(2):121-130. doi:10.1177/1077558717751209 29298545

[7] Busse JW, Wang L, Kamaleldin M, . Opioids for chronic noncancer pain: a systematic review and meta-analysis. JAMA. 2018;320(23):2448-2460. doi:10.1001/jama.2018.18472 30561481 PMC6583638

[8] Deyo RA, Mirza SK, Martin BI, Kreuter W, Goodman DC, Jarvik JG. Trends, major medical complications, and charges associated with surgery for lumbar spinal stenosis in older adults. JAMA. 2010;303(13):1259-1265. doi:10.1001/jama.2010.338 20371784 PMC2885954

[9] Chou R. Review: opioids improve chronic noncancer pain, but difference may not be clinically meaningful in most patients. Ann Intern Med. 2019;170(8):JC41. doi:10.7326/ACPJ201904160-041 30986831

[10] Deyo RA, Mirza SK, Martin BI. Error in trends, major medical complications, and charges associated with surgery for lumbar spinal stenosis in older adults. JAMA. 2011;306(10):1088. doi:10.1001/jama.2011.1300 21917578

[11] Lin H, Wang X, Feng Y, . Acupuncture versus oral medications for acute/subacute non-specific low back pain: a systematic review and meta-analysis. Curr Pain Headache Rep. 2024;28(6):489-500. doi:10.1007/s11916-023-01201-7 38190024 PMC11156714

[12] McCullough BJ, Comstock BA, Deyo RA, Kreuter W, Jarvik JG. Major medical outcomes with spinal augmentation vs conservative therapy. JAMA Intern Med. 2013;173(16):1514-1521. doi:10.1001/jamainternmed.2013.8725 23836009 PMC4023124

[13] McDonagh MS, Selph SS, Buckley DI, . Nonopioid Pharmacologic Treatments for Chronic Pain. Agency for Healthcare Research and Quality; 2020. AHRQ Comparative Effectiveness Reviews report No. 20-EHC010. doi:10.23970/AHRQEPCCER228 32338847

[14] Innes GK, Ogden CL, Crentsil V, Concato J, Fakhouri TH. Prescription medication use among older adults in the US. JAMA Intern Med. 2024;184(9):1121-1123. doi:10.1001/jamainternmed.2024.2781 38949837 PMC11217884

[15] Harris E. Study: polypharmacy nearly doubled in 20 years among older adults in US. JAMA. 2024;332(7):524. doi:10.1001/jama.2024.13387 39058505

[16] Arnstein P. Balancing analgesic efficacy with safety concerns in the older patient. Pain Manag Nurs. 2010;11(2)(suppl):S11-S22. doi:10.1016/j.pmn.2010.03.003 20510845

[17] Schofield P, Dunham M, Martin D, . Evidence-based clinical practice guidelines on the management of pain in older people—a summary report. Br J Pain. 2022;16(1):6-13. doi:10.1177/2049463720976155 35111309 PMC8801690

[18] Chen C, Winterstein AG, Lo-Ciganic WH, Tighe PJ, Wei YJ. Concurrent use of prescription gabapentinoids with opioids and risk for fall-related injury among older US Medicare beneficiaries with chronic noncancer pain: a population-based cohort study. PLoS Med. 2022;19(3):e1003921. doi:10.1371/journal.pmed.1003921 35231025 PMC8887769

[19] Cooper C, Chapurlat R, Al-Daghri N, . Safety of oral non-selective non-steroidal anti-inflammatory drugs in osteoarthritis: what does the literature say? Drugs Aging. 2019;36(suppl 1):15-24. doi:10.1007/s40266-019-00660-1 31073921 PMC6509083

[20] Oh TK, Song IA. Impact of prescribed opioid use on development of dementia among patients with chronic non-cancer pain. Sci Rep. 2024;14(1):3313. doi:10.1038/s41598-024-53728-3 38331973 PMC10853162

[21] Wu M, Fan C, Liu H, . The effectiveness of acupuncture for low back pain: an umbrella review and meta-analysis. Am J Chin Med. 2024;52(4):905-923. doi:10.1142/S0192415X2450037X 38790086

[22] Giovanardi CM, Gonzalez-Lorenzo M, Poini A, . Acupuncture as an alternative or in addition to conventional treatment for chronic non-specific low back pain: a systematic review and meta-analysis. Integr Med Res. 2023;12(3):100972. doi:10.1016/j.imr.2023.100972 37637183 PMC10448023

[23] Vickers AJ, Vertosick EA, Lewith G, ; Acupuncture Trialists’ Collaboration. Acupuncture for chronic pain: update of an individual patient data meta-analysis. J Pain. 2018;19(5):455-474. doi:10.1016/j.jpain.2017.11.005 29198932 PMC5927830

[24] Chou R, Huffman LH; American Pain Society; American College of Physicians. Nonpharmacologic therapies for acute and chronic low back pain: a review of the evidence for an American Pain Society/American College of Physicians clinical practice guideline. Ann Intern Med. 2007;147(7):492-504. doi:10.7326/0003-4819-147-7-200710020-00007 17909210

[25] Qaseem A, Wilt TJ, McLean RM, ; Clinical Guidelines Committee of the American College of Physicians. Noninvasive treatments for acute, subacute, and chronic low back pain: a clinical practice guideline from the American College of Physicians. Ann Intern Med. 2017;166(7):514-530. doi:10.7326/M16-2367 28192789

[26] Bäumler P, Zhang W, Stübinger T, Irnich D. Acupuncture-related adverse events: systematic review and meta-analyses of prospective clinical studies. BMJ Open. 2021;11(9):e045961. doi:10.1136/bmjopen-2020-045961 34489268 PMC8422480

[27] Witt CM, Pach D, Brinkhaus B, . Safety of acupuncture: results of a prospective observational study with 229,230 patients and introduction of a medical information and consent form. Forsch Komplementmed. 2009;16(2):91-97. doi:10.1159/000209315 19420954

[28] Amorim D, Amado J, Brito I, . Acupuncture and electroacupuncture for anxiety disorders: a systematic review of the clinical research. Complement Ther Clin Pract. 2018;31:31-37. doi:10.1016/j.ctcp.2018.01.008 29705474

[29] You J, Li H, Xie D, Chen R, Chen M. Acupuncture for chronic pain-related depression: a systematic review and meta-analysis. Pain Res Manag. 2021;2021:6617075. doi:10.1155/2021/6617075 33680223 PMC7925064

[30] Zhao FY, Spencer SJ, Kennedy GA, . Acupuncture for primary insomnia: effectiveness, safety, mechanisms and recommendations for clinical practice. Sleep Med Rev. 2024;74:101892. doi:10.1016/j.smrv.2023.101892 38232645

[31] Teets RY, Nielsen A, Mah D, . Recruitment and retention for an acupuncture trial in an underrepresented 65 and older population with chronic low back pain. Glob Adv Integr Med Health. 2025;14:27536130251340921. doi:10.1177/27536130251340921 40353070 PMC12064889

[32] Davis AC, Voelkel JL, Remmers CL, Adams JL, McGlynn EA. Comparing Kaiser Permanente members to the general population: implications for generalizability of research. Perm J. 2023;27(2):87-98. doi:10.7812/TPP/22.172 37170584 PMC10266863

[33] DeBar LL, Justice M, Avins AL, . Acupuncture for chronic low back pain in older adults: design and protocol for the BackInAction pragmatic clinical trial. Contemp Clin Trials. 2023;128:107166. doi:10.1016/j.cct.2023.107166 36990274 PMC10416311

[34] MacPherson H, Altman DG, Hammerschlag R, ; STRICTA Revision Group. Revised Standards for Reporting Interventions in Clinical Trials of Acupuncture (STRICTA): extending the CONSORT statement. PLoS Med. 2010;7(6):e1000261. doi:10.1371/journal.pmed.1000261 20543992 PMC2882429

[35] Krebs EE, Lorenz KA, Bair MJ, . Development and initial validation of the PEG, a three-item scale assessing pain intensity and interference. J Gen Intern Med. 2009;24(6):733-738. doi:10.1007/s11606-009-0981-1 19418100 PMC2686775

[36] Callahan CM, Unverzagt FW, Hui SL, Perkins AJ, Hendrie HC. Six-item screener to identify cognitive impairment among potential subjects for clinical research. Med Care. 2002;40(9):771-781. doi:10.1097/00005650-200209000-00007 12218768

[37] National Coverage Determination. Acupuncture for chronic lower back pain (cLBP). Centers for Medicare & Medicaid Services. 2020. Accessed February 7, 2025. https://www.cms.gov/medicare-coverage-database/view/ncd.aspx?NCDId=373

[38] Nielsen A, Ocker L, Majd I, . Acupuncture intervention protocol: consensus process for a pragmatic randomized controlled trial of acupuncture for management of chronic low back pain in older adults: an NIH HEAL Initiative funded project. Glob Adv Health Med. 2021;10:21649561211007091. doi:10.1177/21649561211007091 34104574 PMC8161858

[39] Morley JE, Malmstrom TK, Miller DK. A simple frailty questionnaire (FRAIL) predicts outcomes in middle aged African Americans. J Nutr Health Aging. 2012;16(7):601-608. doi:10.1007/s12603-012-0084-2 22836700 PMC4515112

[40] Yurkovich M, Avina-Zubieta JA, Thomas J, Gorenchtein M, Lacaille D. A systematic review identifies valid comorbidity indices derived from administrative health data. J Clin Epidemiol. 2015;68(1):3-14. doi:10.1016/j.jclinepi.2014.09.010 25441702

[41] Roland M, Fairbank J. The Roland–Morris Disability Questionnaire and the Oswestry Disability Questionnaire. Spine (Phila Pa 1976). 2000;25(24):3115-3124. doi:10.1097/00007632-200012150-00006 11124727

[42] Ostelo RWJG, Deyo RA, Stratford P, . Interpreting change scores for pain and functional status in low back pain: towards international consensus regarding minimal important change. Spine (Phila Pa 1976). 2008;33(1):90-94. doi:10.1097/BRS.0b013e31815e3a10 18165753

[43] Guy W. Clinical Global Impressions, *ECDEU Assessment Manual for Psychopharmacology*, revised. National Institute of Mental Health; 1976;218-222. DHEW Publication No. ADM 76-338.

[44] Yu L, Buysse DJ, Germain A, . Development of short forms from the PROMIS™ sleep disturbance and Sleep-Related Impairment item banks. Behav Sleep Med. 2011;10(1):6-24. doi:10.1080/15402002.2012.636266 22250775 PMC3261577

[45] Cook KF, Jensen SE, Schalet BD, . PROMIS measures of pain, fatigue, negative affect, physical function, and social function demonstrated clinical validity across a range of chronic conditions. J Clin Epidemiol. 2016;73:89-102. doi:10.1016/j.jclinepi.2015.08.038 26952842 PMC5131708

[46] Kroenke K, Spitzer RL, Williams JB. The Patient Health Questionnaire-2: validity of a two-item depression screener. Med Care. 2003;41(11):1284-1292. doi:10.1097/01.MLR.0000093487.78664.3C 14583691

[47] Skapinakis P. The 2-item Generalized Anxiety Disorder scale had high sensitivity and specificity for detecting GAD in primary care. Evid Based Med. 2007;12(5):149. doi:10.1136/ebm.12.5.149 17909240

[48] Cherkin DC, Sherman KJ, Avins AL, . A randomized trial comparing acupuncture, simulated acupuncture, and usual care for chronic low back pain. Arch Intern Med. 2009;169(9):858-866. doi:10.1001/archinternmed.2009.65 19433697 PMC2832641

[49] Cherkin DC, Sherman KJ, Kahn J, . A comparison of the effects of 2 types of massage and usual care on chronic low back pain: a randomized, controlled trial. Ann Intern Med. 2011;155(1):1-9. doi:10.7326/0003-4819-155-1-201107050-00002 21727288 PMC3570565

[50] Sherman KJ, Cherkin DC, Erro J, Miglioretti DL, Deyo RA. Comparing yoga, exercise, and a self-care book for chronic low back pain: a randomized, controlled trial. Ann Intern Med. 2005;143(12):849-856. doi:10.7326/0003-4819-143-12-200512200-00003 16365466

[51] Meier U. A note on the power of Fisher’s least significant difference procedure. Pharm Stat. 2006;5(4):253-263. doi:10.1002/pst.210 17128424

[52] Liang K-Y, Zeger SL. Longitudinal data analysis using generalized linear models. Biometrika. 1986;73(1):13-22. doi:10.1093/biomet/73.1.13

[53] Zou G. A modified Poisson regression approach to prospective studies with binary data. Am J Epidemiol. 2004;159(7):702-706. doi:10.1093/aje/kwh090 15033648

[54] Justice M, Piccorelli A, Avins AL, . Baseline sample characteristics for the BackInAction pragmatic trial of acupuncture for chronic low back pain in older adults. Contemp Clin Trials. Published online June 6, 2025. doi:10.1016/j.cct.2025.107981 40484274 PMC12303732

[55] Skelly AC, Brodt ED, Kantner S, Diulio-Nakamura A, Mauer K, Shetty KD. Systematic Review on Noninvasive Nonpharmacological Treatment for Chronic Pain: Surveillance Report 2: Literature Update Period: October 2021 through December 2021. Agency for Healthcare Research and Quality; 2019. 36893299

[56] Skelly AC, Brodt ED, Kantner S, Diulio-Nakamura A, Mauer K, Shetty KD. Systematic Review on Noninvasive Nonpharmacological Treatment for Chronic Pain: Surveillance Report 3: Literature Update Period: January 2021 through March 2022. Agency for Healthcare Research and Quality; 2022. 36893300

[57] Jones CMP, Day RO, Koes BW, ; OPAL Investigators Coordinators. Opioid analgesia for acute low back pain and neck pain (the OPAL trial): a randomised placebo-controlled trial. Lancet. 2023;402(10398):304-312. doi:10.1016/S0140-6736(23)00404-X 37392748

[58] Caplan Z, Rabe M. The older population: 2020. 2020 Census Briefs. C2020BR-07. US Census Bureau, US Department of Commerce; May 2023. Accessed December 16, 2024. https://www2.census.gov/library/publications/decennial/2020/census-briefs/c2020br-07.pdf

[59] Lee B, Kwon CY, Lee HW, . Concerns about the use of verum acupuncture points in sham acupuncture studies for pain conditions: findings and insights from network meta-analysis. Integr Med Res. 2025;14(1):101122. doi:10.1016/j.imr.2025.101122 39944110 PMC11815645

[60] Freedland KE, King AC, Ambrosius WT, ; National Institutes of Health Office of Behavioral and Social Sciences Research Expert Panel on Comparator Selection in Behavioral and Social Science Clinical Trials. The selection of comparators for randomized controlled trials of health-related behavioral interventions: recommendations of an NIH expert panel. J Clin Epidemiol. 2019;110:74-81. doi:10.1016/j.jclinepi.2019.02.011 30826377 PMC6543841

[61] Sox HC, Lewis RJ. Pragmatic trials: practical answers to “real world” questions. JAMA. 2016;316(11):1205-1206. doi:10.1001/jama.2016.11409 27654606

[62] Zuidgeest MGP, Welsing PMJ, van Thiel GJMW, ; WP3 of the GetReal consortium. Series: pragmatic trials and real world evidence: paper 5. Usual care and real life comparators. J Clin Epidemiol. 2017;90:92-98. doi:10.1016/j.jclinepi.2017.07.001 28694123

[63] Nielsen A, Wieland LS. Cochrane reviews of acupuncture are dated, do not account for the specific effects of sham controls and likely underestimate the efficacy of acupuncture therapy. Integr Med Res. 2025;14(3):101195. doi:10.1016/j.imr.2025.101195 40689021 PMC12271578

[64] Liou KT, Korenstein D, Mao JJ. Medicare coverage of acupuncture for chronic low back pain: does it move the needle on the opioid crisis? J Gen Intern Med. 2021;36(2):527-529. doi:10.1007/s11606-020-05871-6 32378010 PMC7878593

