# Supplementary material for: Acupuncture for Chronic Low Back Pain in Older Adults: A Randomized Clinical Trial
Source: JAMA Netw Open. 2025 Sep 12;8(9):e2531348. doi: 10.1001/jamanetworkopen.2025.31348 (PMC12432643; doi:10.1001/jamanetworkopen.2025.31348)
Supplement: Supplement 1. — Trial Protocol [file jamanetwopen-e2531348-s001.pdf]

Study Protocol

Pragmatic Trial of Acupuncture for Chronic Low Back Pain in Older Adults

(BackinAction)

Principal Investigators:

Andrea J. Cook, PhD

Senior Investigator

Kaiser Permanente Washington Health Research Institute

Lynn L. DeBar, PhD, MPH

Distinguished Investigator

Kaiser Permanente Center for Health Research

Supported by:
The National Center for Complementary and Integrative Health

1UH3 AT010739

May 30, 2020

Tool Revision History

Version Number: 1.2
Version Date: May 7, 2021
Summary of Revisions Made:

1. Updated the Study Team Roster to reflect the current team
2. Added an additional inclusion and an additional exclusion criterion
3. Revised Outcome Measures to streamline questionnaire/reduce participant burden (remove PROMIS Pain Intensity, Pain Interference, Anxiety, Depression and replace with PEG, PHQ-4)
4. Updated the Precis and Study objectives to reflect use of the PEG as a core secondary outcome measure
5. Minor modifications and clarifications on recruitment and consenting procedures and connecting patients with acupuncturists for the first visit
6. Minor modification on compensation schedule for interviews. The total amount remains the same, but prospective participants are now paid for the baseline assessment ($15) and each major follow-up assessment is $5 less than originally proposed
7. Clarification regarding the electronic assessments of hospitalizations for SAE's that are at least probably related to acupuncture
8. Minor clarification re data management
9. Updated the reporting requirements for reportable events to align with KPNC IRB requirements

Version Number: 1.3
Version Date: June 23, 2021
Summary of Revisions Made:

1. Added an inadvertently omitted exclusion criterion from the screener (a fever most days in the last month), which is red flag of serious underlying illness
2. Removed any remaining references to former measures of pain intensity and pain interference and replaced them throughout with the 3-item PEG, which is now an outcome for the baseline and all follow-up questionnaires
3. Clarified a few questions related to recruitment, randomization and Serious Adverse Event reporting

Version Number: 1.4
Version Date: July 7
Summary of Revisions Made:

1. Clarified in a few more places that the biostatisticians will be masked to treatment group assignment until the data base is locked.

Version Number: 1.5
Version Date: March 2022
Summary of Revisions Made:

1. Updated information related to aim 3 to conduct optional, one-time, 60-minute phone interviews with study participants to learn about their study and acupuncture experiences.
2. Updated the protocol for clarity and consistency with current IRB-approved processes.
3. Updated study team roster.

Version 1.6
Version Date: June 2022
Summary of Revisions Made:

1. Updated aim 3 with modest change to scope with elimination of patient interviews to be conducted later in the study to corroborate findings from earlier patient interviews (summative evaluation).
2. Updated study team roster.
3. Updated recruitment targets to reflect a possible increase in total study enrollment in addition to possible increases in recruitment targets at individual sites.
4. Updated study measures table to be consistent with approved procedures for use of acupuncture and adverse event questions.

Version 1.7
Version Date: January 2023
Summary of Revisions Made:

1. Updated PIs listed on cover page.
2. Updated study team roster.
3. Included language to distinguish the participant formative interviews from the acupuncturist formative surveys and interviews.

Version 1.8
Version Date: September 2023
Summary of Revisions Made:

1. Added statement that the cost-effectiveness analysis will be conducted by Dr. Patricia Herman at RAND using de-identified data.
2. Changed institutional affiliation of Alice Pressman to KP School of Medicine.

Version 1.9
Version Date: January 2024
Summary of Revisions Made:

1. Included details of the stakeholder interviews.
2. Added data sharing section describing planned public release database.
3. Added new study team member at RAND.

LIST OF ABBREVIATIONS

AAP	Acupuncture Advisory Panel
AE	Adverse Event/Adverse Experience
CEC	Core Executive Committee
CFR	Code of Federal Regulations
CPT	Current Procedural Terminology
cLBP	Chronic Low Back Pain
CMS	Centers for Medicare and Medicaid Services
CRC	Clinical Research Coordinator
DSMB	Data and Safety Monitoring Board
EA	Enhanced Acupuncture
EBT	Evidence Based Treatment
EHR	Electronic Health Record
FQHC	Federally Qualified Health Center
GAD-2	Generalized Anxiety Disorder 2-item screener
GEE	General Estimating Equations
HCS	Health Care System (refers to IFH, KPNC, KPWA, SH)
HHS	Health and Human Services
HIPAA	Health Information Portability and Accountability Act
ICD	International Classification of Diseases
ICH	International Conference on Harmonization
IFH	Institute for Family Health
IMC	Independent Monitoring Committee
IRB	Institutional Review Board
KPNC	Kaiser Permanente Northern California
KPWA	Kaiser Permanente Washington
LBP	Low Back Pain (duration unspecified)
NCCIH	National Center for Complementary and Integrative Health, NIH
NIA	National Institute of Aging, NIH
NIH	National Institutes of Health
NIH HCS-CCC	NIH Health Care Systems Collaboratory Coordinating Center
OHRP	Office of Human Research Protections
OA	Older Adults (65 and older)
PC/PCP	Primary Care / Primary Care Provider

PEG	Pain, Enjoyment, General Activity (pain scale)
PHQ-2	Patient Health Questionnaire 2-item depression screener
PI	Principal Investigator
PM	Project Manager
PROMIS	Patient Reported Outcomes Measurement Information System
PRO	Patient-Reported Outcomes
RE-AIM	Reach, Effectiveness – Adoption, Implementation, Maintenance
RMDQ	Roland Morris Disability Questionnaire
RTF	NIH Research Task Force for Low Back Pain
SA	Standard Acupuncture
SAE	Serious Adverse Event/Serious Adverse Experience
sFTP	Secure File Transfer Protocol
sIRB	Single IRB (required for multi-site trials)
SH	Sutter Health
SID	Study Identifier
SMC	Statistical Methods Committee
STRICTA	Standards for Reporting Interventions in Clinical Trials of Acupuncture
UMC	Usual Medical Care

TABLE OF CONTENTS

Page

Study Protocol **1**

Pragmatic Trial of Acupuncture for Chronic Low Back Pain in Older Adults **1**

Tool Revision History 2

LIST OF ABBREVIATIONS **4**

TABLE OF CONTENTS **6**

STUDY TEAM ROSTER 9

PARTICIPATING STUDY SITES 10

1. STUDY OBJECTIVES **13**

1.1 Aim 1: Primary Objective 13

1.2 Aim 1: Secondary Objectives 13

2. BACKGROUND AND RATIONALE **13**

2.1 Background on Condition, Disease, or Other Primary Study Focus 13

2.2 Study Rationale 14

3. STUDY DESIGN **15**

4. SELECTION AND ENROLLMENT OF PARTICIPANTS **16**

4.1 Inclusion Criteria 16

4.2 Exclusion Criteria 17

5. STUDY INTERVENTIONS **22**

5.1 Interventions, Administration, and Duration 22

5.2 Handling of Study Interventions 25

5.3 Concomitant Interventions 25

5.3.1 Allowed Interventions 25

5.3.3 Prohibited Interventions 25

5.4 Assessment of Adherence 25

6. STUDY PROCEDURES **26**

6.1 Schedule of Evaluations 26

6.2 Description of Evaluations 27

6.2.1 Screening Evaluation 27

6.2.2 Enrollment, Baseline, and/or Randomization 28

6.2.4 Follow-up Assessments 35
6.2.5 Completion/Final Evaluation 35

7. SAFETY ASSESSMENTS 35

7.2 Methods and Timing for Assessing, Recording, and Analyzing Safety Parameters 36

7.4 Reporting Procedures 38

7.5 Follow up for Adverse Events 38

7.6 Safety Monitoring 39

8. INTERVENTION DISCONTINUATION 39

9. STATISTICAL CONSIDERATIONS 39

9.1 General Design Issues 39

Aim 2 Objective: To conduct a cost-effectiveness analysis of enhanced acupuncture and standard acupuncture compared to usual care. 39

9.2 Sample Size and Treatment Assignment Procedures 39

Treatment Assignment Procedures 40

9.3 Definition of Populations 41

9.4 Interim Analyses and Stopping Rules 41

9.5 Outcomes 41

9.5.1 Aim 1: Primary Outcome 41
9.5.2 Aim 1: Secondary Outcomes 41

10. DATA COLLECTION AND QUALITY ASSURANCE 45

10.1 Data Collection Forms 45

10.2 Data Management 45

10.3 Quality Assurance 47

10.3.1 Training 47
10.3.2 Quality Control Committee 48
10.3.3 Metrics 48
10.3.4 Protocol Deviations 49

11. PARTICIPANT RIGHTS AND CONFIDENTIALITY 49

11.1 Institutional Review Board (IRB) Review 49

11.2 Informed Consent Forms 49

11.3 Participant Confidentiality 50

11.4 Study Discontinuation 51

12. COMMITTEES **51**

13. PUBLICATION OF RESEARCH FINDINGS **52**

14. DATA SHARING **52**

15. SUPPLEMENTS/APPENDICES **61**

1. Acupuncture Treatment Protocol with Acupoints **61**

I.Acupuncture Intervention Guidelines with Acupoints

II. Acupuncture Treatment Visit Form

III. Informed Consent Form Template (not yet available)

STUDY TEAM ROSTER

Name	Address		
Kaiser Permanente Washington Health Research Institute	1730 Minor Avenue Suite 1600, Seattle, WA 98101		
	**Phone number**	**Fax**	**Email**
Andrea Cook, PhD (Multiple PI; Biostatistician)	(206) 287-4257		andrea.j.cook@kp.org
Clarissa Hsu, PhD (Qualitative Researcher)	(206) 287-4276		clarissa.w.hsu@kp.org
Douglas Kane	(206) 442-4075		douglas.e.kane@kp.org
Carolyn Eng, PhD	(919) 225-0778		carolyn.m.eng@kp.org
Robert Wellman, MS	(206) 287-2557		robert.d.wellman@kp.org
Laurel Hansell	(206) 442-5208		laurel.d.dillon-sumner@kp.org
Morgan Justice, MA	(256) 508-4613		morgan.j.fuoco@kp.org
Annie Piccorelli, PhD	(206) 287-2885		Annie.V.Piccorelli@kp.org
Chi Tran, MHI			chi.q.tran@kp.org
Luesa Healy	(206) 287-2794		luesa.l.jordan@kp.org
Juanita Trejo, MPH	(206) 287-2329		juanita.i.trejo@kp.org
Kaiser Permanente Center for Health Research	3800 North Interstate Portland, OR 97227		
	**Phone number**	**Fax**	**Email**
Lynn DeBar, PhD, MPH (Multiple PI)	(206) 287-2426		lynn.debar@kpchr.org
Sutter Health Research Development and Dissemination Center for Health Systems Research	2121 North California Blvd Suite 310, Walnut Creek, CA 94596		
	**Phone number**	**Fax**	**Email**
Katie Stone, PhD, MA (Site PI)	(833) 942-2844		StoneKL@sutterhealth.org
Shruti Vaidya, MS	(925) 287-4031		vaidyas@sutterhealth.org
Lisa Dean			deanl@sutterhealth.org
Alex Scott			alex.scott@sutterhealth.org
The Institute for Family Health	230 West 17th Street, New York, NY 10011		
	**Phone Number**	**Fax**	**Email**

Ray Teets, MD (Site PI)	(212) 206-5200	347-917-1906	rteets@institute.org
Arya Nielsen, PhD (Acupuncture Expert)	(914) 388-2816		arya@guasha.com Arya.Nielsen@mountsinai.org
Matthew Beyrouty, MPA	(212) 659-1409	212-659-9071	mbeyrouty@institute.org
Ariel Jacobs	(646) 483-6558		ajacobs@institute.org
Donna Mah, DACM, Lac (Clinical Site Coordinator)	(917) 453-1778		Mahdonnam@gmail.com
Kaiser Permanente School of Medicine	98 S. Los Robles Ave. Pasadena, CA 91101		
	**Phone number**	**Fax**	**Email**
Alice Pressman, PhD, MS	(925) 287-4018		Alice.R.Pressman@kp.org
Kaiser Permanente Northern California Division of Research	2000 Broadway, Oakland, CA 94612		
	**Phone Number**	**Fax**	**Email**
Andrew Avins, MD, MPH (Site PI)	(510) 891-3557	510-891-3606	andy.l.avins@kp.org
Gabriela Sanchez	(510) 891-3435	(855) 414-2842	Gabriela.X.Sanchez@kp.org
Mahesh Bulbule, MS	(510) 891-5958	510-891-3606	Mahesh.S.Bulbule@kp.org
RAND Corporation	1776 Main St, Santa Monica, CA 90401		
	**Phone Number**	**Fax**	**Email**
Patricia Herman, PhD (Cost-effectiveness Expert)	(310) 393-0411		patricia_herman@rand.org
Samuel Mann, PhD			mann@rand.org

PARTICIPATING STUDY SITES

Please see above

PRÉCIS

After a formative year to refine procedures, we will conduct a pragmatic trial of two types of acupuncture, standardized (SA) and enhanced (EA), compared to Usual Medical Care (UMC) in 789 older adults. The randomized trial tests the value of both groups of acupuncture (SA, EA) for improving back pain related disability relative to usual medical care.

Study Title

Pragmatic Trial of Acupuncture for Chronic Low Back Pain in Older Adults

Objectives

1. Determine the effectiveness of a standard course of acupuncture (SA: up to 15 sessions of acupuncture over 3 months) and an enhanced course of acupuncture (EA: up to an additional 6 sessions of acupuncture over months 4-6) in improving back-related disability in older adults with chronic low back pain (cLBP) compared to usual care alone. Key secondary aims are to determine the effectiveness of acupuncture in improving a composite score of pain intensity and pain interference.

2. Determine the cost-effectiveness of both types of acupuncture versus usual medical care.

3. Describe, understand and explain the barriers and facilitators to adoption, implementation, and sustainability of acupuncture treatment for older adults from patient and acupuncturist perspectives.

Design and Outcomes

In this pragmatic, three-arm parallel groups multi-site randomized controlled trial, we will recruit and randomize 789 adults ≥ 65 years of age with cLBP to SA, EA or UMC alone in four health-care systems. These include two integrated health care systems (HCSs), a fee-for-service system and a network of federally qualified health centers.

Objective 1 outcome data will be collected by questionnaire at 3-, 6-, and 12-months post-randomization. In addition, short monthly surveys will capture data on physical function and a composite score of pain intensity and pain interference.

Our primary outcome will be back-related dysfunction measured by the Roland Morris Disability Questionnaire and our primary timepoint will be 6 months. Key secondary outcomes include the PEG scale (a composite three-item scale assessing pain and pain interference). Other secondary outcomes will include the 6-item PROMIS physical function scale and patient global impression of change. Tertiary outcomes will include the two PROMIS 29 measures (fatigue, social function), the PHQ-4 screener for anxiety and depression and monthly measures of physical function and the PEG scale. We will collect data (EuroQol patient reported outcome scale and health care utilization from the electronic health record) for conducting a cost-effectiveness analysis (Objective 2). We will collect qualitative data from interviews and focus groups that are pertinent to our third objective.

Interventions and Duration

SA will consist of 3 months (90 days) of acupuncture needling, with a proposed minimum of 8

treatments and a maximum of 15. EA will include the standard acupuncture plus an additional 3-month maintenance period, with a proposed minimum of 4 additional acupuncture needling treatments and a maximum of six. UMC will consist of the care that individuals receive according to their insurance benefits plus anything else they pay for out of pocket. We will ask those assigned to UMC to avoid acupuncture for the year they are enrolled in the study. Both active treatment arms (SA, EA) will also have access to UMC as described above. Participants will be enrolled in the study for 12 months.

Sample Size and Population

We will include patients at least 65 years of age with uncomplicated cLBP with or without radiculopathy. We plan to enroll a total of 789 participants (263 per study arm), with the total number enrolled not to exceed 820 participants. Participants will be recruited from four health plans, with varying numbers of participants from each site. Randomization will be stratified by health care system, age category and gender. We expect the racial and ethnic mix will roughly parallel that of the older Medicare population.

1. STUDY OBJECTIVES

1.1 Aim 1: Primary Objective

We hypothesize that both types of acupuncture will result in improved back-related disability compared to usual medical care at 6 months. We further hypothesize that enhanced acupuncture will be superior to standard acupuncture, albeit not expected to be a clinically important difference.

1.2 Aim 1: Secondary Objectives

To examine the effectiveness of acupuncture at 3 and 12 months for improving back-related disability and to evaluate additional outcomes, including a composite score of pain interference and pain intensity at 3, 6 and 12 months.

1.3 Aim 2:

To conduct a cost-effectiveness analysis of enhanced acupuncture and standard acupuncture compared to usual care.

1.4 Aim 3:

To utilize qualitative methods to understand, describe and explain barriers and facilitators to adoption, implementation, and sustainability of acupuncture treatment for older adults from patient and acupuncturist perspectives.

2. BACKGROUND AND RATIONALE

2.1 Background on Condition, Disease, or Other Primary Study Focus

Back pain is the leading cause of disability worldwide, with both prevalence and burden increasing with age.[1] In the United States, about $86 billion is spent annually on direct costs of medical care for back/neck pain,[2] with particularly marked escalating costs for back pain in older Americans (those at least 65 years of age). While the Medicare population increased only 42% between 1991 and 2002, expenditures for back pain increased 387%.[3] Despite these large investments in care for back pain,[4] the health and functional status of Americans with back pain has deteriorated.[2] Roughly a quarter of older adults report low back pain[5] with prognosis worsening with age.[6] Persistent pain, typically lasting three months or longer, is the most consequential type of back pain.

A critical gap exists in the evidence on the safety and effectiveness of treatments for older adults with cLBP. This gap is of particular concern because about 12% of adults age 65 and over suffer from impairing cLBP,[7] the prevalence is increasing[8] and older adults commonly have more disabling back pain than those under age 65.[6] Many treatments considered appropriate for adults under age 65 may not be appropriate for older adults given their greater prevalence of comorbidities with attendant polypharmacy.[9] In addition, burgeoning imaging rates reveal incidental pathology in many cases, placing older adults at risk for inappropriate invasive treatments.[8,10] Because of normal physiological changes with aging (e.g., reduced tolerance of medications and increased prevalence of osteoporosis), older adults are at substantially increased risk of adverse effects of commonly used LBP treatments[7,11-13] including medications (e.g., non-steroidal anti-inflammatory drugs [NSAIDs], muscle relaxants, and opioids) and complementary and integrative (CIM) treatments such as high velocity spinal manipulation techniques. While numerous randomized clinical trials (RCTs) have evaluated treatments for

chronic pain, their applicability to older adults is unclear because most of these RCTs included few, if any, older adults.

Thus, research is needed to clarify the cLBP treatments that are safe and effective for older adults. Evidence-based strategies that incorporate a biopsychosocial approach are recommended to address chronic pain most effectively for a general adult population.[14,15] Studies focused on nonpharmacological treatments may be especially beneficial, given concerns about medication safety.[16] Research demonstrates that older adults are open to trying nonpharmacological therapies,[17] yet few studies of these treatments are published.[18] More research will help clinicians focus on the safest, most effective and most acceptable treatments for older adults, resulting in improved outcomes and fewer adverse experiences.

Evidence-based guidelines from 2017[19] recommended 13 nonpharmacological treatments for cLBP, including acupuncture. Acupuncture is considered to have moderate evidence of effectiveness for improving pain and function compared to usual care.[20] These therapies are now recommended as first-line therapy for cLBP.

In a survey to evaluate the important outcomes for people with chronic pain, participants identified numerous aspects of daily life, including sleep quality, emotional well-being, ability to participate in everyday activities, enjoyment of life, decreased fatigue, and minimizing cognitive difficulties as things that were important to them.[21] Some evidence exists that acupuncture treatment can improve many of these concerns, though studies are typically small and low quality. These outcomes may be especially important to older adults.

2.2 Study Rationale

Acupuncture has been found effective for cLBP in adults largely under 65 years old, with moderate effect sizes, using individual patient level data-analysis, with little diminution of effectiveness over 12 months of follow-up.[22] However, few studies of acupuncture have either included or focused on adults 65 years of age and older. In an unpublished subgroup analysis from the individual patient level meta—analysis conducted by Acupuncture Trialist Collaboration that focused on LBP, Vickers (personal communication) found that only 2.4% of trial participants were 75 years or older.

The optimal dose of acupuncture is unknown for any condition. Large trials of acupuncture for cLBP have typically included 10[23-25] to 12 treatments [26,27] with some using up to 15.[28-30] In a feasibility trial comparing different doses of acupuncture (4, 7, 10 treatments) for cLBP,[31] the largest improvements in function, pain intensity, and pain bothersomeness were seen with 10 treatments. Many trials have focused on acupuncture needling. Our standard course of acupuncture needling will include up to 15 visits over the course of 3 months. This maximum is based on the prior trials as well as the belief of acupuncturists that older adults with more co-morbidities may take longer to improve. We will encourage acupuncturists to treat patients for a minimum of 8 treatments, which is based on data from the AADDOPT-2 trial.[32] We will allow variability in the number of treatment sessions so that the acupuncturist and patient can, within the study guidelines, select the number of treatment sessions optimal for any particular patient.

A critical question is whether long-term outcomes from a course of acupuncture can be improved by offering “maintenance treatment” over a longer period of time, as recommended clinically in some circumstances. No studies have examined this issue. We allow a maximum of six acupuncture needling treatments during this additional 3-month period, anticipating that the frequency of sessions will be tapered during the maintenance phase. We suggest that

acupuncturists see patients at least four times.

We plan to compare our acupuncture groups (SA alone or EA [standard treatment followed by maintenance treatment] to UMC alone. Although there is no accepted standard treatment for cLBP in older adults, we have developed a flexible approach for our treatments with the help of an experienced Acupuncture Advisory Panel. Treatment parameters are described in more detail in Section 5.

Acupuncture has an excellent safety profile. Several large studies collected data on adverse events (AEs) from more than 235,000 patients[33,34] and studies involving more than 63,000 treatments are reported from 156 providers.[35,36] Collectively, this work found that minor AEs, for example bleeding or needle pain, are the most common, in the range of 1–10 in 100 for bleeding and hematoma, and 1–10 in 1000 for strong pain during needling.[34] Hopton[37] found no evidence that patients, who reported bothersome treatment reactions, were less willing to try acupuncture again than those who did not, suggesting that most patients perceive common AEs as minor. In a large trial of acupuncture for cLBP, 2.3% of patients reported a moderate AE that was likely due to treatment and 1 of 477 reported pain lasting one month.[25] Serious AEs, such as pneumothorax, persistent nerve pain, or needle breakage are very rare (typically 1 in 100,000 or less).[34]

CMS recently published an Acupuncture-related National Coverage Decision for the treatment of chronic low back pain.[38] Acupuncture is now covered by Medicare, and over time will become increasingly available to many older adults. In addition, older adults are interested in acupuncture[39] and Medicare is interested in data on the value of acupuncture for older adults with cLBP. They cited the NIH plans to study this intervention in their National Coverage Decision. Acupuncture was cost-effective in two large cLBP trials.[30,40] A critical question is whether long-term outcomes from a course of acupuncture can be improved by offering “maintenance treatment” for a period, as recommended clinically in some circumstances. No studies have examined this issue. This pragmatic RCT will offer clear guidance about the value of acupuncture for improving functional status and reducing pain intensity and pain interference for older adults with cLBP. This evidence will provide essential information for Medicare regarding their coverage decision and for individual physicians and patients deciding on a course of treatment.

3. STUDY DESIGN

We propose a three-arm multi-site parallel design, pragmatic trial with 789 participants to evaluate the effectiveness of two groups of acupuncture to improve functional outcomes (primary outcome), and pain intensity and pain interference (key secondary outcomes) of older adults (≥65 years) with cLBP. We will compare SA (3 months of acupuncture, with up to 15 visits) and EA (3 months of SA, 3 months maintenance acupuncture with up to 6 visits) to UMC (each group, N=263). Participants will be recruited from four healthcare systems (HCSs): Kaiser Permanente Washington - KPWA and Kaiser Permanente Northern California - KPNC, which have Kaiser Permanente integrated health plans; Sutter Health- SH, a largely fee for service organization; and the Institute for Family Health - IFH, a network of federally-qualified health centers (FQHCs). Study participants will be individually randomized with randomization stratified by HCS, age, and gender. The primary outcome will be back-related disability, measured by the Roland Morris Disability Questionnaire (RMDQ) with a primary time point of 6 months. Key secondary outcomes include pain interference and pain intensity and back-related disability outcomes at 3 and 12-months. Thus, we will be able to assess the value of both a course of standard acupuncture (up to 15 sessions over 3 months of treatment) and of the addition of

maintenance acupuncture (up to 6 additional treatment sessions over months 4-6) for improving outcomes. We will include some pre-planned subgroup analyses to shed light on groups that may particularly benefit from acupuncture and those that do not. We will also conduct a cost-effectiveness analysis of both types of acupuncture compared to UMC and a formative and summative analysis to help us understand important facilitators and barriers for broader scale implementation of acupuncture – should it prove useful.

Participants will remain in the trial for 12 months and we anticipate the duration of enrollment and follow-up to be 29 months. We anticipate that acupuncture will be administered in outpatient clinics within participating HCSs and acupuncturist's offices in the community.

4. SELECTION AND ENROLLMENT OF PARTICIPANTS

We plan to recruit a total of 789 participants with chronic low back pain that has persisted for at least three months. Participants will be recruited from four health care systems: IFH (n =123); KPNC (n=288); KPWA (n=174) and SH (n=204). The number of participants recruited from each site may increase by up to 25% from these targets at IFH, KPNC, and SH and may increase by up to 50% from the recruitment target at KPWA with the total number not to exceed 820 participants.

For Aim 3, we plan to recruit and interview 10 participants from each of the four health care systems (n=40) that have been randomized to an acupuncture arm (SA or EA) to learn about their study and acupuncture experiences. We also plan to recruit, survey, and/or interview up to 60 study acupuncturists across the four health care systems to learn about their experience delivering acupuncture as part of the study and more broadly in their community. We will recruit national stakeholders including policy experts, acupuncturists, clinicians that provide acupuncture, and acupuncture researchers that have a vested interest in acupuncture policy around treating older adults to learn more about the impact of the CMS policy for reimbursement for acupuncture treatment on the national landscape for acupuncture reimbursement. We will recruit up to 50 and interview up to 25 stakeholders.

4.1 Inclusion Criteria

We will require all participants to meet all the following inclusion criteria in order to participate in the trial.

Inclusion Criteria	Rationale and Source
Is at least 65 years of age	*Age range of the Medicare older adult population (EHR)*
Is a current member or patient of the healthcare system	*A method for identifying participants who have current and consistent contact with the healthcare system (EHR and PRO)*
Visited a health care provider for low back pain within the past 12 months	*A method for identifying potential participants who may have cLBP (EHR)*
Received primary care at one of the	*Location of our study sites (EHR)*

participating health care systems.	
Has back pain that is uncomplicated with or without radicular pain.	*This is the type of back pain we are studying. (EHR)*
Back pain ≥ 3 months	*Meets our definition of chronic back pain (PRO)*
General activity question from PEG ≥3	*Meets minimum definition of back dysfunction (PRO)*
Primary care provider provides permission to contact patient	*Ensures that there is no medical or related reason not to include patient (via email)*
Willing and able (Callahan screener ≥ 3) to provide consent	*Ethical requirement (PRO)*

Aim 3 inclusion criteria: In order to participate in the patient participant interviews for the Aim 3 study activities, a participant must have been randomized to a treatment arm (SA, EA) for the main trial. In order to participate in the acupuncturist survey and interview, acupuncturist must have been eligible to treat study participants as part of the trial. We will recruit stakeholder participants that have a vested interest in acupuncture policy around treating older adults.

4.2 Exclusion Criteria

Persons who meet any of the exclusion criteria at baseline will be excluded from study participation.

Exclusion Criteria	Rationale
Specific types of back pain (metastatic cancer or bone cancer or secondary cancers, vertebral fractures, spinal infection, active inflammatory disease)	Other treatments are more appropriate than acupuncture for these specific causes (or likely causes) of LBP (EHR)
Low back surgery within past 3 months	May still be healing from surgery (PRO)
Receiving workers compensation or involved in litigation related to cLBP	Additional treatments may be required as there are disincentives to improve (PRO)
Acupuncture within the last 6 months	Ensures that they have not received acupuncture for this episode of care (PRO)
Does not speak or write English or Spanish	Cannot complete assessments re outcomes questionnaires or treatments (PRO)
Major psychosis, dementia	Unable to give adequately informed consent (EHR/provider or PRO)

Current cancer treatment	Need for primary focus on cancer treatment (EHR)
Red flags of serious underlying illness (a fever most days in the last month; recent unexplained weight loss of 10 lbs or more)	Need to look for serious underlying illness to not delay any needed treatment for those. (PRO)
Living in a nursing home, on Hospice, or palliative care	Requires a different study design and logistics (EHR; confirm via PRO)
Non-speaking deafness	Cannot communicate with acupuncturists and study staff (EHR)
Non-reliable transportation	Cannot attend acupuncture treatments (PRO)

Aim 3 exclusions criteria: Participants randomized to UMC are not eligible to participate in Aim 3 study activities.

4.3 Study Enrollment Procedures

4.3.1 Participant Identification and Recruitment

4.3.1.1 Recruitment Targets: We plan to recruit a total of 789 older adults from one of four participating HCSs. Targeted enrollment will differ by the size of the health care system as follows: IFH (n=123); KPNC (n=288); KPWA (n=174) and SH (n=204). The number of participants recruited from each site may increase by up to 25% from these targets at IFH, KPNC, and SH and may increase by up to 50% from the recruitment target at KPWA with the total number not to exceed 820 participants. We will monitor recruitment on a weekly basis and make adjustments on targeted enrollment between sites if we are falling behind on our targets at three-month intervals. We have ascertained that these HCSs had over 92,000 older adults who made an ambulatory care visit for LBP in the 12-month period from December 2018 to November 2019 and who met our electronic inclusion and exclusion criteria. With conservative estimates of 72% ineligible via interview (largely because of LBP not chronic or too mild) or primary care provider (PCP) refusal, we would still have over 25,000 patients who were likely eligible. All patients who are contacted will be provided a final baseline status (unable to reach, ineligible – with reasons, eligible and refused, eligible and randomized). This information will be kept in the study database.

For Aim 3, we plan to recruit and interview 10 participants from each of the four health care systems (n=40) that have been randomized to an acupuncture arm (SA or EA). We also plan to recruit up to 60 study acupuncturists for the acupuncturist surveys and interviews. We plan to recruit across the four health care systems to learn about their experience delivering acupuncture as part of the study and more broadly in their community. We plan to recruit up to 50 and interview up to 25 national stakeholders for the stakeholder interviews.

4.3.1.2. Identification of prospective participants and recruitment procedures

We will use several methods for recruiting participants that have been successful in our previous studies. These include referrals from PCPs; EHR identification of potential candidates followed by letters or emails of invitation; and patient self-referral from direct outreach. Each

site may have some slightly different variations in outreach to prospective participants and the basic pattern of outreach is described below.

Method 1: Referral from PCPs: IFH has had good success recruiting patients for acupuncture research and other integrative health interventions by referrals from PCPs. In addition, we will explore whether PCPs at other sites might wish to refer patients and will develop relevant study processes to accommodate if relevant.

Recruitment procedures: Dr. Ray Teets, the site PI at IFH and a PCP, will inform his colleagues of the study and basic requirements for enrollment. PCPs will use basic eligibility criteria to guide patient referral to the study; an Epic smart phrase will be developed for PCPs that gives a brief synopsis of the study and phone number for the clinical research coordinator (CRC). PCPs will share the CRC's phone number with interested patients. In addition, PCPs will obtain permission from interested patient's to be contacted by the CRC. The CRC will check the patient's EHR to ensure that the patient meets basic inclusion/exclusion criteria and then send provisionally eligible patients an invitational letter with information about the study. The CRC will then call these patients referred for the study. Using a structured script, the CRC will ascertain continued interest in the study. If the prospective participant is interested in participating in the study, they will be asked to provide oral consent for screening and be administered the oral eligibility screener, which captures data that are not in the electronic health record (EHR). If the prospective participant meets the oral screening criteria and agrees to learn more about the study, they will be scheduled for a phone visit to have their questions answered and provide oral consent for the baseline and follow-up data collection, including HIPAA authorization. For study participants randomized to acupuncture and receiving at least one treatment, written consent for acupuncture treatments would be obtained at the first acupuncture visit and before treatment. IFH will also allow for the possibility of using method 2 (below), if recruitment via provider referral is not robust enough, *i.e.,* if recruitment and enrollment targets are not being met.

Method 2: Invitational letters (or emails) sent after identification of candidate participants using ICD-10 diagnoses from automated data: At KPNC, KPWA and SH, we will then send letters of invitation (KPNC, KPWA, SH) or emails of invitation via "MyChart" (SH), a HIPAA compliant secure web-based platform. At these participating HCSs, we expect this will be our primary method of recruitment.

Recruitment procedures: Following approval of a HIPAA Waiver, we will use EHR data to identify members at least 65 years of age who have a) made a visit to a PCP in the last year for pain consistent with non-specific uncomplicated low back pain with or without radiculopathy, and b) appear to otherwise meet eligibility criteria for the study (see sections 4.1 and 4.2). Patient's PCPs will be up to two weeks to remove any patients they deem inappropriate in participating HCSs that require or prefer such PCP review. We will then mail (or securely email) a recruitment packet to patients at each site who meet the EHR-derived eligibility criteria delineated above. The packet will include a description of the study and an informational letter that includes all elements of informed consent, including a clear statement of the option to opt out of further contact for recruitment to this study by calling the provided site-specific study telephone number or returning a statement of interest form and checking the box that indicates their desire to opt out. The invitational letter states that patients might be contacted to determine their interest in participating in the study if they have not called the study contact number or sent their return form indicating either their interest in participating or that they wish to opt-out. Invitational materials will include a HCS site specific telephone number patients can call to get more information about the study. If the prospective participant is still interested, whether they called in, sent in a statement of interest form or were contacted by HCS recruitment staff, oral

informed consent will be obtained before any screening questions are asked. For those meeting study screening criteria, the study staff will guide them through the consent checklist and obtain oral consent for the baseline and follow-up interviews as well as the electronic health record information needed by the study. This consent will be documented in the patient's baseline study record. For some sites, mailed paper consent or electronic written consent via REDCap and/or Adobe sign will be obtained for the study, instead of oral consent.

Method 3: Other potential outreach to target population
We will consider augmenting study recruitment using posters and study brochures available throughout pertinent clinics in our participating HCSs providing a summary of the key study elements and inviting people to contact the study team to learn more about the study. Patients will be screened and, if eligible, enrolled as described in Method 1 (IFH) or Method 2 (KPNC, KPWA, SH)

As noted, we anticipate differences in recruitment procedures between IFH (PCP in-person or MyChart referral) and other performance sites. In addition, there may be some slight differences between sites in the recruitment procedures (e.g., KPNC and SH ask that study contact PCPs before their patients receive an invitation for the study while the director of primary care at KPWA requested that the study not contact PCPs (due to burden and that acupuncture delivery is outside healthcare system). For KPNC and SH, the PCPs will have the option of declining to have specific patients contacted if the PCP believes that participating in the study would not be in the patient's best interest.

Aim 3 recruitment procedures
Patient participants: A study programmer will identify potentially eligible participants across the four health systems via BackInAction study data. We will recruit participants randomized to a treatment arm (SA, EA) for the main trial. Study staff will mail an invitation letter and information sheet to potential participants. Potential participants can call the study line to indicate that they are interested in participating. If study staff do not hear from potential participants within 3-5 working days of the mailed invitation, they will call participants to follow-up and see if they are interested in participating in Aim 3 study activities. Study staff will make up to three call attempts to reach potential participants; call attempts will be spaced 2-3 working days apart.

Acupuncturist participants: KPWA study staff will send the study acupuncturists a recruitment email with an information sheet and a survey link The email will have a brief description of the purpose for the survey and interview with contact information so that they may ask questions before continuing with the survey. Acupuncturists that complete the survey will be asked if they would like to participate in an interview. The study team will select a sample of acupuncturists that select "Yes" for the interview and invite them to complete an interview.

Stakeholder participants: National stakeholders that have a vested interest in acupuncture policy for older adults will be identified through contacts of the study team at all study sites. KPWA or KPNW study staff will send stakeholders a recruitment invitation email Stakeholder participants will be asked to respond to the recruitment email or follow a link to complete a short REDCap interest survey if they are interested in participating in a study interview.

4.3.2. Eligibility screening and consent procedures

Study staff will ask for verbal consent for the oral eligibility screen, which will be done by telephone. The status of all participants will be collected, including reasons for oral ineligibility (e.g., back pain not $\geq$ 3 months, activity limitation too mild, etc.). If oral consent and HIPAA authorization is permitted at some sites, we will then go over the elements of informed consent in the telephone interview, answering any questions that the patient has and documenting oral consent in the database. Otherwise, we will obtain electronic written consent as described above. Finally, if that is difficult, we will send written consent forms to patients to sign before proceeding with the baseline interview and randomization.

<u>Aim 3 research activities</u>
<u>Patient Participants:</u> Study staff will obtain oral consent from participants for the Aim 3 phone interviews and document it in REDCap. Study staff will discuss the phone interview with participants using a recruitment phone script. The script includes a thorough description of the research activities and gives the participant the chance to ask questions regarding the interviews. In addition, the script contains clear language that participation in the interviews is voluntary and will in no way impact the rights of the participant, their access to care at their health care institution, or impact their participation in the main trial. Study staff will also confirm participant's permission to participate in and record the interview before starting the phone interview.

<u>Acupuncturist participants:</u> KPWA study staff will send the study acupuncturists a recruitment email with an information sheet attached and the survey link embedded at the end of the email. The email will have a brief description of the purpose for the survey and interview with contact information so that they may ask questions before continuing with the survey. Acupuncturists will be given the opportunity and encouraged to ask questions via email or by phone to the study PM sending the recruitment email. The information sheet provides information about their rights and protections as participants, and additional detail regarding the purpose of the study. Acupuncturists will give consent to participate in the Aim 3 acupuncturist survey in the REDCap form and verbal consent for the interview. Consent for both the survey and interview will be documented in REDCap by study staff. Acupuncturists that complete the survey will be asked on the survey form to indicate that they have read the information sheet and that they asked questions if needed by selecting a “Yes” or “No” response at the beginning of the survey in REDCap. On the survey form, the project manager's (PM's) contact information will again be made available for the acupuncturist to ask questions prior to answering questions on the survey. The study team will select a sample of acupuncturists that select “Yes” for the interview and invite them to complete an interview.

Phone interviews will be recorded by the study team via Teams or with an audio recording device. The recording will begin after the interviewer briefly reviews the information sheet with the acupuncturist to check whether they have any questions, and after the acupuncturist gives verbal consent to participate in the interview and be recorded. The KPWHRI staff performing the interview will take notes and will document in REDCap whether the acupuncturist participant gave verbal consent to both participate and record the interview.

<u>Stakeholder participants:</u> KPWA or KPNW study staff will send stakeholders a recruitment invitation email with an information sheet attached that provides information about the study procedures, potential risks and benefits, voluntary participation, confidentiality, and the option to withdraw at any time. Stakeholder participants will be given the opportunity and encouraged to ask questions via email or by phone to the study staff sending the recruitment email. Stakeholder participants will be asked to respond to the recruitment email or follow a link to complete a short REDCap interest survey if they are interested in participating in a study

interview. Prior to beginning the recording, stakeholders will give verbal consent for the interview.

Phone interviews will be recorded by the study team via Teams or with an audio recording device. The recording will begin after the interviewer briefly reviews the information sheet with the participant to check whether they have any questions and after the participant gives verbal consent to participate in the interview. The participant will be asked to give verbal consent to be recorded. While the participant must give verbal consent to participate in the interview, they do not need to consent to be recorded. If the individual consents to the interview but not to be recorded, the interviewer will take notes to capture as much of the interviewee's responses as possible. The KPWHRI or KPNW staff performing the interview will take notes and will document in REDCap whether the stakeholder participant gave verbal consent to both participate and record the interview.

4.3.4. Randomization

One of our study biostatisticians will prepare the randomization scheme for each site using a generic code of Arm 1, 2, 3. The unmasked database programmer can then assign a code to each of the study arms so that the biostatisticians remain blinded to treatment group. The randomization scheme will be embedded in the computer program at the end of the baseline interview. It will include stratified blocked randomization based on site, age group (65-74; 75-84; 85+), and sex. Block sizes will be randomly varied. The study interviewer will press a button and the appropriate group assignment will appear. (In order to mask the follow-up interviewer to participants' group assignments, a different interviewer will administer those interviews. This has worked well in our previous trials[25,41]). Patients randomized to EA will be informed whether or not they were selected to receive additional maintenance treatment sessions close to the end of the first 3 months of treatment (approximately 10 weeks into the study) so that their treatment is unlikely to be altered by the knowledge of additional visits. In addition, acupuncturists will be masked to SA or EA group assignment until 10 weeks into the SA period so that they don't provide different treatments in the standard treatment group based on whether the participant has a maintenance period or not.

4.3.5 Assignment to Acupuncture

At KPNC, KPWA and SH, participants who are randomized to acupuncture will be provided with the names of those acupuncture providers who have experience treating chronic low back pain and working with older adults, have agreed to see study patients, and who are conveniently located for patients. Patients will then select an acupuncturist from those listed as study affiliated and the study will fax a study referral to the acupuncturist with the patient information. Minor variations such as scheduling the first visit online for the patient or calling the acupuncture office with the patient on the call will be permitted. At IFH, acupuncture is provided onsite and patients will be scheduled on site.

4.3.6. Screening and Consent Procedures

These are described in section 6.2.1.

5. STUDY INTERVENTIONS

5.1 Interventions, Administration, and Duration

Acupuncture will be individually administered to patients by a licensed acupuncture practitioner in outpatient HCSs clinics or community acupuncturists' offices.

5.1.1. Standard acupuncture (SA):

Our SA intervention is based on classical Chinese acupuncture therapy and was developed with a team of experts using a modified Delphi process. It involves up to 15 acupuncture treatments over 3 months with the total number being determined by the patient and acupuncturist together. (Fifteen treatments is consistent with the greatest number of allowed treatments in some of the largest acupuncture trials to date.[28-30]) We encourage, however, a minimum of 6-8 treatments in the first 8 weeks,[23,42] with at least 1-2 treatments in the last 4 weeks.[24,25] The Acupuncture Advisory Panel recommended that we consider 8 treatments as the minimum for determining if the patient is not a responder to acupuncture. Treatment visits will typically last 45-60 minutes. All treatments will include only acupuncture needling, even though other adjunctive modalities (e.g., moxibustion or other forms of heat, cupping, gua sha, tui na) are typically part of practice.[43] Treatments will be provided at medical facilities or in private acupuncturists' offices.

Our general approach is classical Chinese acupuncture that includes palpation of the channels and acupoints. We used a modified Delphi process to refine our planned acupuncture intervention over three phone meetings and an additional round of email. Acupuncture Advisory Panel (AAP) members included nine members: expert physician and licensed acupuncturists representing diverse backgrounds (e.g., work with underserved populations, university clinics, work inside of health plans, medical director of a holistic medicine network, academic dean at an acupuncture college) and an acupuncture researcher who has numerous publications on acupuncture treatments for cLBP. We provided the AAP with data on acupuncture interventions from well-designed trials of acupuncture for cLBP,[25,26,29,31,44-52] how a group of experienced China trained practitioners report treating older adults with cLBP[53] and how each of the group members described how they would treat older adults with cLBP (from a survey). We used the STRICTA checklist[54] for reporting acupuncture trials as a guide for organizing our discussions and plan to capture the information necessary to report the treatment provided in the trial according to the STRICTA checklist.

Acupuncturists are expected to begin each visit with the intake questions (aka "asking" interview) and follow with other assessments (range of motion observation, palpation of the region, channels and Hara). This will be followed by point selection and needling (with the characteristic *de qi* at practitioner discretion); needle retention; removal of needles and resting or optionally, changing position to treat another part of the body, further palpation and point selection and needling (de qi at practitioner discretion); needle retention, removal of needles, and resting. Acupuncturists are expected to check in with patients at the end of the session, assessing pain and range of motion and a patient's readiness to leave the treatment room, provide any self-care recommendations, and confirm the next appointment. We expect treatment sessions to include both local and distal acupoints[55] and that specific point combinations will vary between treatment sessions and per patient. We recommend non-coated needles and will capture data on typical needle characteristics (gauge, length).

Through the Delphi process, our Acupuncture Advisory Panel recommended including a total of 113 named acupoints (214 acupoints total if counting bilaterally): low back acupoints (29; 58 if bilateral; 4 central for total of 62), acupoints on the mid and upper back (33; 66 if bilateral; 2 central for total of 68), front of the body including distal leg acupoints (33; 66 if bilateral; 6 central points for total of 72) and ear acupoints (6; 12 if bilateral).These are shown in Appendix

1. Ashi points (tender upon palpation) are also permitted. Other acupoints can be selected if additional rationale is provided. We anticipate 6 to 20 needle insertion sites will be used in each treatment, although the first may involve fewer needles. If patients are treated in one body position, needle retention time is typically 20-30 (up to 40) minutes. If patients are treated in two body positions, needle retention time is typically 15-20 (up to 25) minutes.

5.1.2. Enhanced Acupuncture (EA):

The EA intervention includes the 3 months of standard acupuncture plus an additional 3 months of maintenance acupuncture, which can include up to 6 treatments to be spaced at least every other week (tapered). The maintenance acupuncture treatment sessions are expected to consist of the same intervention used in the first 3 months of care described above.

We are unaware of any trials of acupuncture for cLBP that have evaluated the use of maintenance acupuncture, even though acupuncturists often recommend maintenance visits after a course of more intensive treatment. Berman and colleagues[56] conducted one of the few studies that included a gradually tapering protocol as part of a knee osteoarthritis trial. In that study, treatments decreased from twice weekly to weekly to every other week to monthly. Based on discussions with our AAP, we propose allowing up to 6 treatments during the 3-month maintenance phase.

<u>Integration of Acupuncture into Care and Study Acupuncturists:</u> Each of our participating HCSs has a distinct way of providing general acupuncture services for adults. The most common practice in our HCSs and nationally is to refer patients to acupuncturists practicing in the community and we will use this process for many of our study visits. At IFH, acupuncture will be provided in the FQHC primary care clinics. Acupuncture will be provided in individual treatment sessions in all locations.

Acupuncturists will have at least five years of experience working at least 50% of the time in patient care. There may be exceptions for 3 years' experience per individual applicant, for example with other health care licensure. They will need to be state licensed, malpractice insured (with no current or historic malpractice claims) and experienced in treating patients with chronic low back pain and who have multi-morbidities. They will also need to be experienced working with older adults. In addition to the above, they will be vetted by the health plans and/or lead acupuncturist (for example, at KPWA, we will recruit from our network of providers and at IFH, we will recruit from prior trials).They will all be trained in the protection of Human Subjects for research, as well as the study protocol, special safety issues for our study, and the logistics of delivering and recording study treatments. They will be certified for participation in the study.

<u>Capturing acupuncture treatment visit information:</u>

As a pragmatic trial, patients are not required to complete all acupuncture visits to stay in the study. An intent-to-treat approach to analysis will be used. Data for each visit at KPNC, KPWA and SH will be collected from the treating acupuncturist in a secure, SQL server HIPAA-compliant online database, which in turn will trigger a payment to the acupuncturist for the acupuncture session. Acupuncturists working at IFH clinics will chart in the EHR using a specially designed EPIC template. Data will be securely submitted following each session with the following information: date, visit number, visit length, visit disposition (e.g., completed, cancelled, no show), elements of the visit (e.g., asking interview, palpation of acupoints), selected needling details (e.g., number of needles, retention time, acupoints needled, whether this was this the last recommended treatment), and patient reported adverse events. Based on

ours and others previous studies[25,32,34], we expect that patient reported AEs are most likely to involve minor pain, dizziness, itching or bruising after needling, transitory exacerbation of pain, or fatigue.

The collected information will allow us to fully characterize acupuncture treatment practices and may provide important information for secondary exploratory analyses examining patient responsiveness to treatment. We have developed and used similar treatment visit forms for other studies,[57-59] and have worked with our acupuncture advisory group during the UG3 year to refine these data collection forms and processes for the study. Minor changes were made after the pilot studies were completed. A copy of the acupuncture visit form is shown in Appendix 2.

Trial acupuncturists' fidelity to agreed upon practice parameters will be routinely and systematically assessed via their completion of the online, standardized study forms for each treatment session.

Any acupuncture care participants may seek independent of the study would be subject to usual care billing and documentation. However, no patients nor patient insurance policies will be billed for study-related visits, as consistent with other pragmatic trials conducted in the participating health systems.

5.2 Handling of Study Interventions

Acupuncture sessions will be delivered in the clinics of the participating HCSs or in the private offices of acupuncturists in the community. All treatments will be charted electronically.

5.3 Concomitant Interventions

5.3.1 Allowed Interventions

Participants in all groups will be able to access all treatments available to them as part of their insured health care offerings as well as anything they wish to pay for out of pocket.

5.3.3 Prohibited Interventions

We will also ask study acupuncturists to avoid using specific modalities of treatment that are often used in Chinese medicine as adjuncts to needling such as moxibustion, cupping, and gua sha. We will ask participants in the usual care group to avoid acupuncture over the course of the study.

5.4 Assessment of Adherence

Adherence will be defined for both the standard acupuncture and the maintenance phases of the intervention. For the standard acupuncture period, an adherent participant will be characterized as one who receives at least 8 treatments (but not more than 15) and the acupuncturist indicates that treatment is complete or the participant receives at least 12 treatments (80% of the 15 potential treatments). For the maintenance acupuncture phase, adherent participants will be characterized as those who receive at least 4 treatments (and not more than 6) or for whom the acupuncturist indicates that the treatment is completed. Participants in the EA group who have, according to the acupuncturist, completed treatment in the standard period will still be expected to attend maintenance acupuncture sessions.

Allowable ranges of adherent treatment have been characterized because we expect that the number of treatment sessions in standard or maintenance acupuncture will vary between patients. Participants in the EA group whose acupuncturist stated that treatment was complete in the first 3 months (standard period) will still be asked to attend maintenance treatments because that tests the value of the maintenance period for long-term benefits on study outcomes.

We will assess adherence by several means: attendance at acupuncture visits and the content of those visits. Because different participants might require different numbers of visits, we will ask the acupuncturists at each visit to let us know whether the patient is continuing treatment, has completed treatment or is unlikely to improve (including before and after at least 8 treatments as recommended by the Acupuncture Advisory Panel). This will allow us to determine whether a patient stops treatment prematurely. Even patients who stop the treatment prematurely in the standard treatment period but are randomized to receive maintenance acupuncture will be encouraged to make acupuncture visits in the maintenance period.
We will assess acupuncturists' fidelity to the treatment protocol by reviewing completed treatment forms at the end of each treatment session, which will include key details of their study visit, the structure of the visit, needling details and acupuncture points used, visit duration, and self-care recommendations (Appendix 2 has a copy of the form). We will also ask patients about their use of non-study acupuncture treatments to characterize any acupuncture received by participants outside study provided services.

6. STUDY PROCEDURES

6.1 Schedule of Evaluations

Assessment	EHR screen	Phone screen	Baseline Phone Survey	Acupuncture Visits	Monthly	3,6, 12 Months
Eligibility	✓	✓				
Informed Consent		✓	✓±	✓*		
Demographic Information		✓	✓			
Baseline Questionnaire		✓	✓			
Randomization			✓			
Monthly Short Questionnaire					✓	
Follow-up Questionnaire						✓
Adverse events				✓		✓

± All aspects of the study will be discussed via phone and prospective participants will be provided with consent checklists. IFH and SH plan to obtain electronic written consent for the study; KPNC and KPWA plan to obtain oral consent for the study interviews and electronic data extraction.

* IFH, KPNC and KPWA will also get written or electronic consent for acupuncture treatments before the first treatment.

6.2 Description of Evaluations

A waiver of consent will be obtained to use electronic health records to pre-screen patients according to the eligibility criteria that can be ascertained via those records. We will have ICD-10 codes and contact information, including name, address and phone number.
.

6.2.1 Screening Evaluation

At all sites, the screening evaluation will be done by telephone after obtaining oral consent for screening. If the patient is interested and eligible, we will conduct a second consent process that further explains the study and answers questions.

Consenting Procedure

If the sIRB permits it (for each HCS where the IRB administrator is in agreement), we plan to obtain oral consent (waived written consent) from prospective participants for participation in the baseline and follow-up interviews. (If required by the local IRB, we will obtain written consent). We would then obtain written consent for acupuncture treatment from any participant who is randomized to either SA or EA and makes at least one visit to the acupuncturist. A study interviewer will administer oral consent to prospective participants, ensure full understanding of study focus and procedures, and answer any questions. The oral consent process will describe the purpose of the study, the procedures to be followed, the risks and benefits of participation and how participants' data will be handled. This information will also have been provided in a study information sheet sent with the initial study invitation letter and will include questions related to acupuncture. Thus, participants who are randomized to acupuncture will sign a consent form that contains information they already reviewed with the study interviewer before they receive treatment. For some participants, this will be similar to consent for treatment documents they have signed for treatment as part of their regular medical care. Acupuncture consent forms will be sent by the acupuncturist to each HCS for storage in locked filing cabinets, At IFH, the participant signed consent to acupuncture clinical treatment form, which is not a research document, will be stored in the participants' EHR.

For sites that permit oral consent (waived written consent), a study interviewer will administer oral consent to prospective participants, ensure full understanding of study focus and procedures, and answer any questions. The oral consent process will describe the purpose of the study, the procedures to be followed, the risks and benefits of participation and how participants' data will be handled. This information will also have been provided in a study information sheet provided to the participant previously.

Participants will be told that they are free to not respond (by web or phone) or to terminate involvement at any time, with no adverse consequences. If a participant appears to be distressed during assessments, research staff will halt the interview and offer to call back to complete the interview. The interview will only recommence when and if the participant reports feeling capable of doing so. The interviews during the study involve no specific risk or discomfort beyond those of a standard clinical interview. Interviews will be conducted by experienced and well-trained staff sensitive to these issues.

Screening

The screening process will typically occur on the same day as randomization (for oral consent for the trial). For patients who are required to have electronic written consent, we will aim to obtain that within one week of screening or administer the back pain eligibility questions again when written consent is obtained more than 30 days after screening. Participants will be asked a series of questions that should determine their eligibility (back pain duration, activity limitations due to back pain, capacity to provide consent, recent low back surgery, receiving workers compensations for cLBP or litigation related to cLBP, acupuncture within last 6 months, does not speak or write English or Spanish, unexplained weight loss of 10 lbs or more, fever; see Section 4.1 and 4.2 for more detail). Any of the questions can make them ineligible.

6.2.2 Enrollment, Baseline, and/or Randomization

Enrollment

Enrollment in the study occurs when an individual is screened eligible, has provided oral (if allowed) or written consent, has completed the baseline assessment questionnaire and has been randomized to a treatment. This should be within a month of eligibility assessment.

Baseline Assessment and Outcomes

Study Measures and Schedule of Administration

DOMAINS	Baseline	Monthly Follow-up	3-, 6- and 12-Month Follow-up	Data Source
**Demographic and Clinical Characteristics**				
Patient Characteristics	✓			EHR & PRO
Medical and Back Pain History	✓			EHR & PRO
Expectations of Acupuncture	✓			PRO
Abbreviated COVID questions	✓		✓	PRO
**Primary, Secondary and Tertiary Measures**				
*** Back-related dysfunction (RMDQ)**	✓		✓	PRO
PEG [CDE]	✓	✓	✓	PRO
Physical Function [CDE]	✓	✓	✓	PRO
PHQ-4 [Anxiety/Depression] [CDE]	✓		✓	PRO
Sleep Disturbance [CDE]	✓		✓±	PRO
Patient Global Impression of Change [pain][CDE]			✓	PRO
Fatigue	✓		✓	PRO
Ability to Participate in Social Roles and Activities	✓		✓	PRO
Patient Global Impression of Change [overall status]			✓	PRO
High Impact chronic pain [CDE]	✓		✓±	PRO
Euro-QOL-5D	✓		✓	PRO
**Treatment-Related Information**				

Adverse Events		✓#	PRO; EHR; Treatment Records
Use of acupuncture during study period		✓^	PRO
Adherence to Assigned Treatment		✓	Treatment Records
**Health Care Utilization**			
Health Care Utilization and Costs	✓	✓	EHR; Medicare Fee Schedule; PRO
Pain-related Health Services, Products & Self-Management Practices	✓	✓	PRO
Daily Exercise and Job-related Activity	✓	✓	PRO
**Other HEAL Common Data Elements (CDE)**			
Pain Catastrophizing Questionnaire (6-item)	✓	✓±	PRO
Substance Use (TAPS)	✓	✓^	PRO

Primary Outcome Measure (Roland Morris Disability Questionnaire)

Blue measures are recommended by the NIH Task Force (RTF)

**CDE=HEAL Common Data Elements (required for all HEAL trials)

PROMIS-29 profile V 2.0, note we will add 2 additional questions to the Physical Function and Sleep Disturbance Measures to ensure measures in these domains reflect core HEAL measures. We will not be asking the Pain Intensity, Pain Interference, Depression or Anxiety Scales

± At 6-month timepoint only (Sleep duration is asked at all timepoints.)

#At 3- and 6-month timepoints only

^At 12-month timepoint only

Our approach to measurement includes adoption of many recommendations by the NIH Research Task Force for Low Back Pain (RTF),[60] the IMMPACT domains for clinical trials of chronic pain[61] and the NIH PRISM HEAL Common Data Elements (CDE). In addition, we plan to harmonize as much as possible with the Optimum trial, which is also a study focused on cLBP. In considering measures to include in our assessment battery, we attempted to adopt as many of the HEAL CDE as we reasonably could while ensuring that constructs pertinent for our study target population were included and that redundancy and potential cognitive load was minimized to reduce burden on our older adult population.

Baseline Interview

When possible, we will obtain sociodemographic characteristics from the EHR including: birthdate (to calculate age), sex, race, and ethnicity but will verify this information at the baseline assessment as well as augment it with the “sex at birth” and “gender identity” questions from the HEAL Common Data Elements (CDE). Other sociodemographic variables will be obtained directly from the participant including: employment status, education, marital status, income, tobacco use, alcohol use, duration of pain condition. Further questions characterizing the

participant's current pain condition (presence of sciatica), potential prognostic risk for pain-related impairment (e.g., pain catastrophizing, fear avoidance, treatment expectations, and disability/workers compensation), and pain-related service use (e.g., back surgery, injections, opioid and other common prescription medications, various types of exercise, psychological counseling, and use of acupuncture) align with RTF recommendations or are specific to the study's focus on acupuncture. We will measure patient expectations of acupuncture using one item from the EXPECT short form[62] and using the term acupuncture as the therapy. The question uses a 0 to 10 scale focused on expectations related to acupuncture treatment for chronic low back pain. We will include questions about the impact of the COVID-19 pandemic on the ability to get health care and on their overall health (physical, emotional, and mental, including pain). These were adapted from a patient questionnaire developed by the Pain Management Collaboratory funded by the NIH – Department of Defense and Veterans Administration[63]. We will ask several questions from the FRAIL scale to characterize participant frailty at the time of enrollment in this study. Finally, we will also collect data from the EHR on other co-morbidities, including various pain conditions. This will allow us to describe the population as well as conduct some moderator analyses.

Aim 1 measures (Baseline AND 3, 6 and 12-monthfollow-up).

Back-related disability will be measured with the 24-item Roland-Morris Disability Questionnaire (RMDQ),[64] which asked whether 24 specific activities were limited due to back pain during the past week (yes or no). This legacy measure has been found to be reliable, valid and sensitive to clinical changes.[65-69] It is appropriate for telephone administration for patients with moderate disability.[70] The RMDQ will be our primary outcome measure and will be measured as a continuous variable. The minimal clinically important difference (MCID) for the RMDQ in our population is 2 points[71] or 30% improvement from baseline.[72] The MCID will be examined in a secondary analysis.

As a secondary outcome measure and a HEAL CDE, we will use the PEG scale (a validated, 3-item pain-intensity and pain-related interference composite measure assessing **P**ain intensity, as well as pain interference with **E**njoyment of Life and **G**eneral Activity.[75]

The PROMIS®, the Patient-Reported Outcomes Measurement Information System, was developed as a set of patient reported outcomes that can assess physical, mental and social health in adults and children.[76,77] In this study, we propose to use four domains of the PROMIS®-29, which has eight outcome domains important for a study of cLBP in older adults. These include physical function, sleep disturbance, fatigue and ability to participate in social roles. All short-form measures we will use are 4-items except for Physical Function. The RTF recommends the use of pain intensity, pain interference, physical function, depression, anxiety, and sleep disturbance, all domains covered by the PROMIS®-29.[60] Deyo et al.[78] found that the PROMIS®-29 measures had good to excellent internal consistency and worse scores among older patients with chronic pain associated with falls, high catastrophizing and worker's compensation, suggesting its value for use with older adults. Hence, we believe the PROMIS measures are best suited for use with our older adult population. However, in the interest of adopting all the required HEAL CDEs, we will add 2-items to the physical function and sleep disturbance questions so that they are the 6-item HEAL core recommended versions of these scales, and substitute the PHQ-4 anxiety depression screener rather than use the PROMIS® anxiety and depression scales. All versions of the multi-item short-forms we are using include the range of mild to severe impairment for that domain and measure at least 3 standard deviations of the T-scores[80], the healthy population plus roughly two standard deviations of the unhealthy population.[81] Correlations between each multi-item short form we are using and the

full-item bank are above 0.9 for all domains except physical function, where the correlation is roughly 0.87.[80] Power for detecting a small (0.2) effect in a clinical population for each multi-item measure with our sample sizes is well over 90%.[80] Construct validity was demonstrated for these measures as well.[80] Below, we describe other features of the individual PROMIS® short form measures we are using.

Physical Function will be measured by the 6-item PROMIS® physical function measure. It has been found to perform well psychometrically compared to the RMDQ.[84] It is a secondary outcome.

Sleep quality will be measured by the 6-item PROMIS® Sleep Disturbance Scale. In a study of community-dwelling older adults,[85] the unidimensional measure was found to have acceptable internal consistency and strong construct validity. It is a tertiary outcome and is asked about at baseline and 6-months. We will ask about sleep duration at baseline and all follow-up timepoints.

Depression will be measured by two items from the PHQ-4[86], which are recommended as a screen for depressive symptoms[87] . Use of this measure will allow us to harmonize with the HEAL CDE. It is a tertiary outcome.

Anxiety, which is a tertiary outcome, will be measured with two items of the PHQ-4.[86] that include two core anxiety items from the GAD-7[88] and will allow calculation of the GAD-2 score. This will allow us to harmonize with the HEAL CDE for anxiety.

Fatigue will be measured by the 4-item PROMIS® Fatigue Scale and will be a tertiary outcome. This is of interest because acupuncture can address fatigue and it is a reported concern for many older adults.

We will assess the *ability to engage in social roles* using the 4-item PROMIS® Ability to Participate in Social Roles subscale, which is also pertinent to older adults because of the importance of social interactions to combat loneliness. This will be a tertiary outcome.

We will also ask two questions about high impact chronic pain (one question is about the number of days of pain in the last 3 months and the number of days with limited activities due to pain in 3 months. Using these plus the PEG, we will be able to classify people in our trial into 3 groups: High impact chronic pain (in the last 3 months, both pain on most or every day and limited activities due to pain most or every day); bothersome chronic pain (in the last 3 months, pain on most or every day <u>and</u> never or some days with activity limitation due to pain <u>as well as</u> PEG score of 12 or higher); mild chronic pain (in the last 3 months, pain on most or every day <u>and</u> never or some days with activity limitation due to pain <u>as well as</u> PEG score of less than 12).[89]

<u>Aim 2 measures:</u>

Measures of both costs and effectiveness are needed for cost-effectiveness analysis. We will use the EQ-5D, one of the most commonly used measures of generic health status, to generate our measures of effectiveness. It measures five dimensions of health status (mobility, self-care, usual activities, pain and discomfort, anxiety and depression) and has five levels for each of these (having no problems to having extreme problems). Scores on each of these dimensions are then weighted using US population preference weights[90] to generate a time point-specific

measure of health-related quality of life or utility. These utility values across the study period are used to calculate quality-adjusted life-years (QALYs), a generic measure of health improvement.

We will be calculating cost-effectiveness from both a health care sector and a Medicare perspective. The health care sector perspective includes all "formal health care sector (medical) costs borne by third-party payers."[91] The Medicare perspective includes all costs Medicare would likely reimburse. Because there is no efficient and timely way to capture non-Medicare-reimbursed costs for the SH and IFH health systems, we will only calculate the health care sector perspective for participants in the Kaiser systems. Measures of *health care utilization* within the health care system (provider visits, imaging studies, prescription medications, costs) will be captured from the electronic health record and/or from Medicare claims data. We will identify and separately include back pain-related costs in our analyses. The use of acupuncture sessions will be captured from study records.

Aim 3 qualitative data collection:

The qualitative work on this study focuses on both formative and summative evaluation questions[92] The evaluation will use the RE-AIM (reach, effectiveness, adoption, implementation, maintenance) model.[94,95]. After the clinical trial is well underway (UH3 year 2), we will conduct interviews and/or virtual focus groups with patients (and potentially with acupuncturists) who have participated in the trial to get input on the implementation of the acupuncture interventions and document the experiences of participating patients (and potentially acupuncturists). Feedback from study acupuncturists may also be solicited through surveys.

The RE-AIM guided evaluation work[94,95] will be initiated in UH3 Year 2. This model has four components: Reach, Effectiveness, Adoption, Implementation, and Maintenance. **Reach** reflects the percentage and characteristics of persons who receive the intervention. We will use EHR and assessment data to document: 1) the percentage of patients excluded from the trial and the rationale for exclusion, and 2) the percentage of patients receiving acupuncture based on the denominator of all patients approached for participation, as well as all potentially eligible patients in each health plan regardless of whether or not they were approached for study participation. **Effectiveness** measures the impact of the intervention on important study outcomes (see Study Measures and Schedule of Administration). Qualitative data can be critical for a richer understanding of quantitative study findings (reach, recruitment and effectiveness). HCS **Adoption** is less relevant for this study as many acupuncture services will be provided outside the HCS clinics (as is standard in these settings). However, there may be valuable lessons to be learned from those sites that choose to offer acupuncture within the HCS clinics (IFH and others) and by tracking relevant organizational and policy changes. Furthermore, adoption may be influenced by patient experiences, which will be ascertained through interviews and/or focus groups conducted with patients (and, potentially, acupuncturists). Finally, **Maintenance** (the ability to sustain acupuncture services in these and broader health care settings), will be assessed through qualitative interviews, survey, and/or focus groups with patients, and/or survey data collection and qualitative interviews with study acupuncturists and, other stakeholders. Interviews with acupuncturists will focus on barriers and facilitators to providing acupuncture under the study and CMS guidelines for reimbursement for acupuncture services for Medicare patients. Stakeholder interviews will focus on understanding factors important for clinical and operational leaders in including provisions for acupuncture treatment in their settings and the way that the CMS policy for reimbursement has affected the ability to provide services to older adults. Further, our acupuncture advisory group can provide input into

important factors for offering acupuncture treatment in a variety of settings (including fee for service).

<u>Patient Participant Interviews</u>
Interviews will be conducted by telephone; patient focus groups will be conducted virtually in real-time using a telephone/video conferencing platform such as Teams.

If a participant is interested in participating in the Aim 3 phone interviews, study staff will schedule a 60-minute phone interview for the participant with the qualitative interview team and email or mail the participant a confirmation letter. Study staff will send an email reminder and/or place a reminder call to participants 1-2 days before their scheduled interview day/time. Phone interviews will be recorded by the study team via Teams or with an audio recording device. The recording will begin after all preliminary information has been reviewed and after the participant gives verbal consent to record and participate in the interview. After the recording has started, the participant will confirm their consent to participating in the interview and the recording of the interview. The recording will be sent to a professional transcriptionist (Jackson Street Associates), with whom we have a Business Associate Agreement established, via secure file transfer to be transcribed. Interview transcripts will be returned to the study team via secure file transfer and stored in a secure project folder on the KP Washington network. Only BackInAction study staff will have access to the transcripts and will remove any identifiable information from the final transcripts. The audio files will be destroyed 5 years after the conclusion of the project. Study staff will add $50 to a participant's ClinCard for completing the phone interview.

<u>Acupuncturist Interviews & Survey</u>

BIA acupuncturists will be invited to complete a survey (hosted on REDCap) and interview. Interviews will be conducted by telephone or Teams call.

If an acupuncturist is interested, they will be asked to complete a survey, estimated to take up to 30 minutes to complete. The acupuncturists will receive an invitation via email with a link to the REDCap survey. The recruitment email provides a clear explanation of the purpose of this survey and interview, as well as it being optional, and encourages them to ask the KPWHRI staff questions if they have any. Acupuncturists that complete the survey will be asked to indicate that they have read the information sheet and asked questions (if needed) by selecting a "Yes" or "No" response at the beginning of the survey in REDCap. The project manager's (PM's) contact information will be made available to request clarifications prior to answering questions on the survey. KPWHRI BIA study staff will document acknowledgement of acupuncturist consent as part of the survey in REDCap, which is for both the survey and the interview (if they indicate interest and are selected for interviewing).

If the acupuncturist selected "Yes" in the REDCap survey to participate in the phone interview, they will be invited to participate in a phone/Teams voice call interview with study staff, estimated to last up to 60-minutes. Phone interviews will be recorded by the study team via Teams or with an audio recording device. The recording will begin after the interviewer briefly reviews the information sheet with the acupuncturist to respond to any questions, and after the acupuncturist gives verbal consent to participate in the interview and be recorded. The KPWHRI staff performing the interview will take notes and will make a note in the REDCap database indicating whether the participant gave verbal consent to both participate and record the interview.

The recording will be transcribed automatically by Teams and then reviewed by study team members. Transcripts will be stored in a secure project folder on the KP Washington network. Only BackInAction study staff will have access to the transcripts and will remove any identifiable

information from the final transcripts. The audio files will be destroyed 5 years after the conclusion of the project.

Study staff will send an incentive of $25 after completion of the survey, and those that complete the interview will receive an additional $50.

Stakeholder Interviews

Stakeholder participants will be invited for a 30-45 minute interview. Phone interviews will be recorded via Teams. The recording will begin after the interviewer briefly reviews the information sheet with the stakeholder participant to respond to any questions, and after the participant gives verbal consent to participate in the interview and be recorded (if the individual consents to the interview but not to be recorded, the interviewer will take notes to capture as much of the interviewee's responses as possible). The KPWHRI or KPNW staff performing the interview will take notes and will make a note in the REDCap database indicating whether the participant gave verbal consent to both participate and record the interview. If the interview is performed at KPNW, the recording will be sent to KPWA via secure file transfer for transcription.

The recording will be sent to a professional transcriptionist (Jackson Street Associates), with whom we have a Business Associate Agreement established, via secure file transfer to be transcribed. Interview transcripts will be returned to the study team via secure file transfer and stored in a secure project folder on the KP Washington network. Only BackInAction study staff will have access to the transcripts and will remove any identifiable information from the final transcripts. The audio files will be destroyed 5 years after the conclusion of the project.

Study staff will send an incentive of $50 with a gift card to the stakeholder participant, if they are eligible to receive incentives, after completion of the interview.

Randomization

Patients will be randomized just after the baseline assessment has been completed. It is our intent that the baseline assessment is completed within a week of the eligibility screen (otherwise, back pain eligibility questions will need to be reassessed). Participants will receive $15 for completion of the baseline assessment. Participants are asked to make their first visit to acupuncture within 10 days of being randomized.

6.2.3 Blinding

This is an unmasked trial for participants, although the acupuncture participants will not know whether they are in the SA group or EA group until 10 weeks into the standard treatment period. Interviewers will be masked to treatment group for all standard study assessments. For qualitative data collection, interviewers are expected to be unmasked to participants treatment condition. Acupuncturists will not know whether their patients are in the SA or EA group until 10 weeks into the standard treatment period.

No members of the Core Executive Team (Multiple PI's, site PI's and the Statistical Methods Committee, which includes the study biostatisticians) will have access to the outcomes data during the trial. Conceivably, a site PI or one of the Multiple PI's may become aware of an individual's treatment group if they have an Adverse Event that requires the investigator intervene. One biostatistician, Mr. Wellman, may be supervising some data collection exercises with the programmers as needed, but datasets will not contain a treatment assignment variable prior to the locking of the database. All other members of the CEC will become unblinded after the data base is locked.

6.2.4 Follow-up Assessments

Each participant will be followed for 12-months post-randomization. Participants will be invited to complete short monthly check-ins per their preferred mode (phone or web) between main assessments at 3-, 6- and 12-months. Monthly check-ins will take less than 5-minutes to complete and will be limited to administration of the PROMIS® Physical Function (6-items) and PEG (3-items). Participants will receive a $5 incentive for each check-in completed typically payable as a lump sum at the end of the study (up to $45 total for the 9 monthly check-ins). Patients will be contacted via their preferred mode (phone or web) for the 3-, 6-, and 12-monthfollow-up surveys. Advance letters will be mailed as reminders to those preferring phone follow-up and emails with the reminder and a live hyperlink will be sent to those preferring to complete follow-ups online. Participants may opt to change their preferred mode at any time. Incentive payments of $20, $25 and $30 will be sent for completing 3-, 6- and 12-month follow-ups, respectively.

6.2.5 Completion/Final Evaluation

The final 12-month assessment will include the same measures as those included in the 3-month follow-up assessment battery.

7. SAFETY ASSESSMENTS

We will monitor patient safety for all individuals during their 12-month enrollment in the trial. Acupuncturists will perform an intake assessment, which will include a thorough understanding of the patient's health relevant to the provision of acupuncture needling. The needling protocol, although broad, is based on care delivered in clinical practice[47,48,55,99] and the highest quality trials.[23,25,29,30,45] Our acupuncturists will be trained on the study protocol and reminded of best practices when treating older adults. They will be able to provide enough treatments to allow them to start with gentle treatments at the beginning for patients who are apprehensive about needling.

We will select acupuncturists who have experience working with older adults and cLBP, and, if applicable, are already credentialed to provide acupuncture for patients within the local health care system. We will ensure that they review universal precautions and care guidelines in older adults. We will make sure that the older adults know how to prepare for their treatment (e.g., be fully satiated and hydrated in advance of a needling session). Although treatment is expected to be focused on needling treatment for cLBP, acupuncturists will tailor treatment to the presentation and needs of each participant. Acupuncturists will use only single-use pre-sterilized needles that are immediately discarded after use. This is already required of all acupuncture providers in the US. They will avoid needling areas of the skin that may have rashes or lesions. Finally, patients whose symptoms significantly worsen or who present with any new symptom will be referred to their PCP for evaluation. Medical care of patients invited to participate in the study will not be affected by their decision whether or not to participate.

7.1 Specification of Safety Parameters

Our study protocol is designed to minimize AEs with this population. Because electrical stimulation is prohibited, the needling treatment will be safe for patients with pacemakers. This study will comply with the national standard requiring acupuncturist to use only single-use (one-time use), pre-sterilized needles that are discarded immediately after use. This practice reduces

risk of transfer of blood-borne pathogens and is already required of all acupuncture providers in all states where this pragmatic trial will be implemented. Acupuncturists are trained to avoid areas of the body where there are rashes, skin lesions or any breach in normal skin barrier. They will have flexibility in positioning the patient for treatment so that it is comfortable for the patient. Finally, patients whose symptoms significantly worsen will be referred to their PCP within their healthcare system.

7.2 Methods and Timing for Assessing, Recording, and Analyzing Safety Parameters

Acupuncture has an excellent safety profile. Collectively, several large studies gathering data on adverse events from more than 235,000 patients[33,34] and involving more than 63,000 treatments provided by 156 clinicians[35,36] found that minor adverse events, for example bleeding or needle pain, are the most common, in the range of 1–10 in 100 patients for bleeding and hematoma and only 1–10 in 1000 for strong pain during needling.[34] Further, Hopton[37] found no evidence that patients who reported bothersome treatment reactions were less willing to try acupuncture again than those who did not, suggesting that most patients perceive common adverse events as minor. In a large trial of acupuncture for cLBP, 2.3% of patients reported a moderate adverse experience that was deemed likely due to treatment and only 1 of 477 reported pain lasting one month.[25] The most commonly reported AEs were short-term pain, with one participant reporting dizziness and another, back spasms. Because acupuncture involves needle insertion, transient pain is possible, especially at acupoints in more sensitive regions of the body. The most serious adverse events, such as pneumothorax, infection, organ or tissue injury, persistent nerve pain, or needle breakage are very rare (typically 1 in 100,000 or less).[34]

AEs reported by patients to their acupuncturists or study staff will be recorded and reported to appropriate regulatory bodies (single IRB and Independent Monitoring Committee (IMC)). Acupuncturists will record AEs on their treatment forms but alert the research team immediately if they are concerned about any AE or learn a participant is injured, needs hospitalization, trip to the emergency department or medical attention. In addition, patients will have the phone numbers of study staff to report any side effects they are concerned about in real time. We will collect data on “health problems that have interfered with your functioning for a few days or more that you think may have been caused by your study acupuncture treatment” using a mailed questionnaire sent to all acupuncture-randomized participants at 3 months after randomization and enhanced acupuncture participants again at 6 months after randomization. Any positive responses will be reviewed by study medical personnel and followed up by study personnel to obtain additional information on the patient’s condition, whether it has resolved or not, how long that took and whether the patient needed to modify their activities. The previous AE form we used for an acupuncture study (which we filled out based on data from the follow-up interview) included: anxiety, bruising, dizziness, fainting, fatigue, headache, nausea/vomiting, pain from needle insertion or stimulation, and other pain (including increased back pain). We graded non-serious AEs as mild, moderate and severe. We also included information on relatedness to treatment (definitely, probably, possibly, definitely not). Note, increased back pain is an expected variation in the natural history of cLBP and, as such, would not without concomitant disability be considered an AE for this study.

7.3 Adverse Events and Serious Adverse Events

Acupuncture can have transient side effects as described in the previous section. For the proposed study, we are operationally defining a serious adverse event (SAE) as a death, hospitalization, prolongation of a hospitalization, or other serious or life-threatening event during a patient’s active participation in the trial. We will review/query active study participants’ EHR

data every month to identify deaths and hospitalizations among enrolled participants. In the case of a death, a chart review will be conducted by a physician at the clinical site to assess whether the death was related to the study intervention (definitely, probably, possibly, or unrelated to the study intervention). For hospitalizations, a study clinician at each HCS will review the list of diagnoses for possible relatedness to the acupuncture intervention. For any diagnosis at least possibly related to acupuncture, a more in-depth examination of the medical chart will be conducted. Because the number of hospitalizations in our older adult study population may be high and acupuncture poses only minimal risk, we do not plan to chart-review hospitalizations as a matter of course. However, if our analytic reports suggest a possible increased risk of hospitalizations associated with acupuncture, we will work with our monitoring groups to develop a plan to do chart reviews on all or a subset of patients charts who were hospitalized. A non-serious AE will be defined as an unfavorable and unintended diagnosis, symptom, syndrome, or disease that occurs or worsens during the acupuncture intervention period and is plausibly related to acupuncture. We believe this definition is appropriate, given that older adults have many diagnoses, symptoms, and syndromes, most of which would be expected to be of longer duration and unrelated to acupuncture, for which AEs have typically been shown to be relatively brief in duration. Non-serious AEs will be collected in multiple ways: (1) at 3 and 6 months using a mailed questionnaire sent to all acupuncture-randomized participants at 3 months after randomization and enhanced acupuncture participants again at 6 months after randomization asking about "health problems that have interfered with your functioning for a few days or more that you think may have been caused by your study acupuncture treatment" ; (2) from participants who may phone the study team at any time to report AEs; and (3) via electronic acupuncturist treatment reports for events that occur or were reported in the acupuncturist's office. Because acupuncture has relatively short-term physiological effects, we will not report AEs that first manifest more than 30 days after a participant's final acupuncture treatment.

We will classify each non-Serious AE using the following definitions: Mild (transient or minimal symptoms; no changes in activity level; no therapy or only symptomatic therapy; Moderate (symptomatic with moderate changes in activity level; no decrease in social activities; specific therapy required); Severe (incapacitating; bed rest; substantial decrease in social activities; loss of work). These definitions are consistent with the International Council for Harmonisation (ICH) standards in characterizing AEs. In the unlikely event that an adverse effect occurs that requires medical care, treatment will be provided according to each participants' existing health care coverage. We will also assess the likely relatedness of the adverse event and acupuncture given the nature of the event, the timing related to treatment and any important contextual factors.

Unanticipated problems that include risks to participants or others are defined by the Office for Human Research Protections as any incident, experience or outcomes that meets all the following criteria:

1. Unexpected in terms of nature, severity, or frequency given (a) the research procedures that are described in the protocol-related documents, such as the IRB-approved research protocol and informed consent document; and (b) the characteristics of the subject population being studied;

2. Related or possibly related to participation in the research ("possibly related" means there is a reasonable possibility that the incident, experience, or outcome may have been caused by the procedures involved in the research); and

3. Suggests that the research places subjects or others at a greater risk of harm (including physical, psychological, economic, or social harm) than was previously known or recognized.

Unanticipated problems will be recorded in the data collection system throughout the trial.

7.4 Reporting Procedures

A report of SAEs will be reviewed by the PIs and Co-Investigators every six months and by the Independent Monitoring Committee (IMC) every six months. Reports of AE's will be reviewed from the 3 and 6-month questionnaires at regular study meetings. They will be signed off by the Site PI and a study physician. If acupuncturists or patients report an AE, study staff will complete an AE form and follow-up with the patient as appropriate. Drs. Avins and Teets, our two physician site PIs and co-investigators and Dr. Nielsen, our acupuncture consultant, will be available as needed to assist with attribution of the AE to acupuncture and guidance on follow-up. Non-serious AE's will be reported to the IRB yearly and to the IMC at the next meeting.

Incidents or events that meet the OHRP criteria for unanticipated problems will be reported to the IRB and the BackInAction (BIA) IMC. We will report Unanticipated Problems to our NCCIH Program Director and the BIA IMC by submission of an Unanticipated Problem Report via fax or email to the IMC chair and via secure email to the NCCIH (Program Officer and OCRA).

Per HHS regulations 46.103(b)(5), the "appropriate time frame for satisfying the requirement for prompt reporting will vary depending on the specific nature of the unanticipated problem, the nature of the research associated with the problem, and the entity to which reports are to be submitted". We will apply the following recommended OHRP and KPNC guidelines to satisfy prompt reporting requirements:

- Unanticipated problems that are breaches of confidentiality or are deaths should be reported to the IRB and NCCIH (Program Director and OCRA) within 1 business day of the investigator becoming aware of the event.

- Any other unanticipated problem should be reported to the IRB and NCCIH (Program Director and OCRA) within 5 business days of the investigator becoming aware of the problem.

- All SAEs that are not unanticipated problems should be reported on a yearly basis to the IRB.

- All unanticipated problems should be reported to appropriate institutional officials (as required by an institution's written reporting procedures), the supporting agency head (or designee), and OHRP within one month of the IRB's receipt of the report of the problem from the investigator.

7.5 Follow up for Adverse Events

For AEs related to acupuncture, we will follow patients from the time we learn about them until they are resolved. In our previous acupuncture studies, virtually all participants' AEs were resolved within 1-2 weeks.

7.6 Safety Monitoring

The 5-member Independent Monitoring Committee has expertise in acupuncture, biostatistics, Medicare data analysis, medicine, pragmatic trials, qualitative methods and research in chronic low back pain. They will have reviewed the protocol prior to commencement of the trial. They will meet on a schedule recommended by NCCIH, generally every 12 months. We anticipate that the IMC will identify reports that they will ask our study team to routinely prepare for their meetings. We expect that an approved IMC monitoring plan will be created by the IMC in conjunction with NIH study sponsors.

8. INTERVENTION DISCONTINUATION

The acupuncture study interventions may be discontinued if recommended by the participant's acupuncturist or PCP. If the patient has a bad reaction to acupuncture (e.g., needle allergy) or is diagnosed with a condition that takes precedence over the acupuncture treatment (e.g., cancer, serious stroke), we will ask that they discontinue the study treatment. Patients can discontinue the intervention for any reason they choose. If possible, we will ask those participants to continue to complete their regularly scheduled outcome assessments for the standard 12-month enrollment period.

9. STATISTICAL CONSIDERATIONS

9.1 General Design Issues

BackInAction (BIA) is a 3-arm multi-site randomized controlled trial of older adults with cLBP. Patients are randomized individually. Aim 1 includes two objectives:

Aim 1 Primary Objective: Both standard and enhanced acupuncture will result in improved back-related disability compared to usual medical care at 6 months. We further hypothesize that enhanced acupuncture will be superior to standard acupuncture, albeit not expected to be a clinically important difference.

Aim 1 Key Secondary Objective: To examine the effectiveness of acupuncture at 3 and 12 months for improving back-related disability and to look at additional outcomes, including the PEG, a 3-item composite measure of pain intensity and pain interference with enjoyment of life and general activity at 3-, 6- and 12-months.

Aim 2 Objective: To conduct a cost-effectiveness analysis of enhanced acupuncture and standard acupuncture compared to usual care.

9.2 Sample Size and Treatment Assignment Procedures

Older adults with cLBP will be randomized, in equal proportions, to one of three groups: Usual medical care (UMC), standard acupuncture (SA), or enhanced acupuncture (EA).

We determined our sample size requirements for our primary outcome RMDQ at 6-months that focuses on detecting differences of each acupuncture group compared to UMC. Given a sample size of 630 total participants (210 per group) we have at least 90% power to detect a minimally clinically important difference (MCID) of two points on the RMDQ[29,72] between each acupuncture group and UMC assuming a SD of 6 in each arm (consistent with results from previous

trials)[25,41,100] and only testing for pairwise comparisons if the omnibus F-test is statistically at a 0.05 α-level (to control for multiple comparisons). Accordingly, we have high power to detect a MCID difference between SA and UMC as well as EA and UMC. Nevertheless, if instead we assume that at 6-months SA attenuates to be equivalent to UMC and EA has a 2-point MCID improvement relative to both SA and UMC we will have 91% power to detect a difference between SA and EA as well as between UMC and EA. Thus, we are sufficiently powered (≥90%) to detect MCID differences between all pairwise group comparisons. Power was calculated via simulation studies using R software.

For any two group comparisons, given our sample size of 210 per group and a SD of 6, the minimal detectable difference is 1.15 pts (i.e. the 95% CI width around difference in means between groups is +/- 1.15 pts). Further, for secondary analysis for the binary outcome 30% improvement in RMDQ from baseline we have >90% power to detect an MCID assuming the probability of improvement in UMC was between 33%[25] and 44%[6] and the MCID was a 15% improvement above UMC for each of the acupuncture groups. Assuming a 20% loss-to-follow-up rate we inflated our sample size to 263 per group (789 total) to assure that we are well powered for all analyses of interest. (Assuming a more conservative 25% loss-to-follow-up rate we would inflate our sample size to 280 per group (840 total) to assure that we are well powered for all analyses of interest.) We will use an intent-to-treat approach in which participants will remain as randomized regardless if they withdrawal from treatment or cross-over to other treatment arm (e.g. UMC participant seeks acupuncture outside of study).

Treatment Assignment Procedures

After completion of baseline questionnaire, participants will be randomized via a computer-generated randomization scheme in R developed by a study biostatistician in a 1:1:1 ratio to study condition (SA, EA, or UMC) stratified by HCS, age (65-74; 75-84; 85+) and sex. Stratification is only being used to maintain balance of treatment assignment with stratum and we do not have sample size requirements within a given stratum. We will employ random blocks of size 3 and 6 to ensure balance of groups over time as well as blinding of study team to next randomization assignment. The biostatistician will keep the randomization file in a secure folder only accessible to the programmer. The study programmer will be given the randomization scheme within specified strata and will only allow participants to be randomized once they consent and complete the baseline questionnaire. The study interviewer will press a button and the appropriate group assignment will appear. This method ensures that treatment allocation cannot be changed after randomization. Further, at the time of randomization those randomized to SA or EA will only be indicated that they are randomized to acupuncture. Patients randomized to EA will not be informed that they have been selected to receive additional maintenance treatment sessions until close to the end of the first 3 months of treatment (approximately 10 weeks into the study) so that their treatment is unlikely to be altered by the knowledge of additional visits.

After the randomization schemes have been generated by the biostatistician, the programmer will be the only one to have access to the randomization schemes that are embedded in the program. The coding will be held in a secure folder. Other study personnel including principal investigators will only receive unmasked summary information after the completion of the intervention and the database is locked. During the IMC reporting treatment assignment will be masked unless requested by the IMC. The programmer will run the reports for the IMC meetings.

9.3 Definition of Populations

The intent-to-treat analysis will include everyone in the study population. The per protocol exploratory analysis will include everyone who adhered to the protocol for the SA, EA, and UMC groups. If someone crossed over into another group (i.e., they received no acupuncture but were randomized to acupuncture or they received acupuncture but were in the UMC arm), they will be considered as part of the group whose treatments resemble theirs as long as they met the requirements for the minimum number of treatments. For adherence analyses we will include 1) participants in the acupuncture groups who, in the 3 months of standard acupuncture, received at least 8 treatments and the last visit indicated that they had completed treatment or received at least 12 treatments and 2) amongst those with last visit indicated that they had completed treatment (see section 5.4. for our proposed operationalization criteria for "completed treatment").

9.4 Interim Analyses and Stopping Rules

We are not proposing any interim analyses and have no stopping rules.

9.5 Outcomes

9.5.1 Aim 1: Primary Outcome

The Roland Morris Disability Questionnaire (RMDQ) is the primary outcome measure. Six months is the primary time point. For our primary outcomes, we will look at changes in the RMDQ score.

9.5.2 Aim 1: Secondary Outcomes

Secondary outcomes include the PEG as well as physical function and Patient Global Impression of Change for Pain. Three and 12 months are secondary time points. We will conduct analyses that look at changes in these scores at 3-, 6-, and 12-months. In addition, we will look at ≥ 30% improvement in the RMDQ and pain intensity measures relative to their baseline values.

9.5.3 Aim 1: Tertiary Outcomes

Tertiary outcomes included PROMIS measure of ability to engage in social roles, anxiety, depression, fatigue, and sleep disturbance. If these back-focused treatments can impact other common complaints of patients with cLBP, then we would expect improvements in these domains if they are sufficiently frequent in our patients. While these are important domains for patients with cLBP, we consider them tertiary because there is little empirical evidence regarding acupuncture's capacity to impact them. In addition, monthly measurements on the PEG and physical function, will be treated as tertiary outcomes and analyzed via exploratory analysis to understand the outcome trajectories.

9.5.4 Aim 2: Outcome Measures

We will measure effectiveness in terms of change in quality-adjusted life-years (QALYs) across the 12-month period calculated using the EQ-5D. Costs will be calculated from both the health care sector and the payer (Medicare) perspectives based on changes in all formal health care

sector (medical) costs and the portion of those costs reimbursed by Medicare, respectively.

9.6 Data Analyses

Aim 1 will evaluate the effectiveness of acupuncture and acupuncture plus maintenance relative to Usual Medical Care (UMC) at 3-, 6- (primary time-point), and 12-months after randomization. We will conduct a longitudinal analysis including the continuous outcome, change in Roland Morris Disability Questionnaire (RMDQ) from baseline (primary outcome) measured at all follow-up times, in one model estimated using generalized estimating equations (GEE). We will use a working independence correlation matrix and will calculate standard errors using the robust sandwich estimator to account for within-person and within-in provider (some participants may see the same provider) correlation.[101] We will include interactions between intervention groups and time (3-, 6-, and 12-months) to estimate time-specific intervention effects. Time will be included as a categorical variable. To gain power, since acupuncture and acupuncture plus maintenance at 3-months are the same intervention (maintenance period occurs between 3 and 6 months post randomization and neither participants in the EA condition nor the acupuncturists treating them will be aware they will receive maintenance treatments until close to the 3-month follow-up), we will combine acupuncture groups at 3-month follow-up. We chose GEE as our analytic method because our primary outcome, RMDQ, is not expected to be normally distributed. From our experience working with RMDQ data, adjusting for baseline RMDQ value results in more normally distributed residuals. However, we didn't want to make that assumption a priori and therefore chose GEE.

For the 6-month time point we will conduct a sequential series of analyses. We will first run a regression model with three groups (acupuncture [SA], acupuncture plus maintenance [EA] and UMC) for the 6-month follow-up. We will then assess differences in change in RMDQ at 6-months between the two acupuncture groups: with (EA) and without (SA) maintenance. If a statistically significant (α=0.05) and meaningful difference (>1 point difference) is found between the maintenance (EA) versus no maintenance (SA) groups (Scenario 1), we will further compare each of the acupuncture groups separately to UMC. Scenario 1 assessments will determine if acupuncture with maintenance (EA) is better than acupuncture without maintenance (SA) at 6-months and if either or both acupuncture groups are better than UMC. If acupuncture groups do not differ at 6-months (Scenario 2), we will combine acupuncture groups for this time point and run a second regression model including only UMC and the combined acupuncture group. If this regression model shows that acupuncture is better than UMC, we will conclude that acupuncture improved RMDQ at 6-months, but maintenance was not shown to be efficacious.

We will follow the same general framework for 12-month as we have specified for 6-months. Note that the analysis focused on the 12-month follow-up timepoint provides an important test of whether maintenance (EA) makes an appreciable difference over standard acupuncture in sustaining or improving the effect of acupuncture on pain-related functioning that endures after the end of treatment. Finally, note that we include all times points in a single model within this general modeling framework to handle correlation due to multiple outcomes on a given person.

To control for multiple comparisons when testing between the three groups at 6-months we will use Fisher's least significant (LSD) difference procedure.[102] Fisher's LSD has been shown to strongly hold the family-wise error rate at α-level for studies with three treatment groups.[103] Fisher's LSD is a simple procedure where the global Wald-test of the null hypothesis of equal means for all groups is performed first. If this overall test is statistically significant then the sequential series of pairwise comparisons will be performed as outlined previously; otherwise the procedure stops, failing to reject the over null hypothesis that at least one mean is different

from the others.

We will conduct a similar analysis for secondary and tertiary outcomes, including key secondary outcomes such as the PEG and will use appropriate link functions for non-continuous outcomes. All models will adjust for baseline outcome value, age, sex, and HCS as well as any baseline variables that are predictive of loss to follow-up. All analyses will be conducted following an intent-to-treat approach, including all individuals randomized regardless of their engagement with, or exposure, to the intervention. If loss to follow-up is above 15%, we will employ imputation techniques to address missing data issues.[104] However, our focus will be on minimizing loss to follow-up and in our collective substantial experience conducting similar trials, we have consistently had retention in line with this. The imputation method that we were referencing uses a pattern mixture approach that relaxes the missing at random assumption. This approach that we propose using is derived for GEE and is sensitive to potential non-ignorable missingness.[104]

Exploratory analyses will further use tertiary outcome data collected monthly (i.e., the PEG and PROMIS®, Physical Function). We will use these measures to assess the trajectory of how long it takes until patients improve and to address questions such as "What proportion of people improve at three months if they don't improve after one or two months of acupuncture?" These exploratory analyses will help address how much acupuncture is needed to improve and at what time, given a patient's outcome trajectory, should acupuncture treatment stop if improvement has not been shown up to that time.

We will conduct a set of prespecified moderator analyses using a similar analysis as Aim 1 except include interactions with the moderator and treatment groups. Moderators of interest include age (65-74; 75-84; 85+), patient expectations and gender. However, we do not anticipate being powered to detect a difference in treatment effects between men and women. According to results from the Acupuncture Trialists Collaboration, gender has not been consistently found to be related to benefits from acupuncture.[105] We will look at race and ethnicity as well, but we will not be powered to detect a difference between groups. We have identified potentially important clinical conditions that may well diminish treatment effect (i.e., cognitive impairment, frail elderly, co-morbid pain conditions, co-morbid depression). If these are sufficiently common in our study participants, we will perform pre-specified moderator analyses.

We will also conduct exploratory per protocol analyses assessing dose response to see if the effect changes dependent upon number of acupuncture treatments received and include UMC as having 0 treatments in the analyses. We will flexibly model the trajectory of the primary outcome at 3 months by number of treatments to see if there is a threshold effect of treatment dose. Further we will run adherence analyses comparing amongst those who adhered in the 3-months of standard acupuncture (at least 8 treatments and last visit indicated by acupuncturist that they completed treatment or at least 12 treatments) compared to UMC and amongst those adhered to those who did not adhere within the acupuncture groups. We will do a second set of adherence analyses but define adhered as only those that the acupuncturist indicated as completing treatment. Since these analyses are not intent-to-treat we will include further potential confounders that are associated with being non-adherent or receiving differential number of treatments.

Aim 2: We will perform economic evaluations from both the payer (Medicare) and health care sector perspectives alongside the randomized pragmatic trial comparing usual medical care (UMC) to the addition of 3-months of acupuncture (SA) and 3-months of acupuncture and 3-

months of maintenance acupuncture (EA) in older adults with cLBP. Costs for the health care sector perspective will include the costs of all healthcare utilization priced using Medicare's national fee for service rates, including acupuncture visits, and costs from the payer perspective will only include the Medicare covered amounts, including the reimbursed costs of acupuncture.[106,107] Effectiveness will be measured using quality-adjusted life-years (QALYs) based on changes (net of baseline)[108] in the preference-weighted[90] values of the EQ-5D[109] over the study year.

If costs of either of the acupuncture arms compared to UMC are reduced and effectiveness increased, we will describe acupuncture as cost saving and to dominate UMC in terms of cost effectiveness.[106] If incremental costs and effectiveness are both increased then an incremental cost-effectiveness ratio will be calculated and compared to society's willingness to pay for an additional QALY ($50,000 to $100,000 per QALY[110]) to see if acupuncture can be considered cost-effective.

Acupuncture implementation costs will be captured from study records in terms of the number of treatments received in 15-minute units and valued at typical community rates. For the payer perspective, we will use several assumptions regarding the amount of these costs reimbursed by Medicare with our base case based on typical acupuncture reimbursement rates (or trial allowable costs per Medicare). We would prefer to capture all healthcare utilization and cost data for the year before and after baseline from CMS for each patient in the study. However, this is unlikely given the time constraints of our project and when Medicare data are available. In addition, Medicare/CMS data will not include healthcare utilization for those not currently on Medicare (e.g., still on employer insurance or getting care at the VA). Since KPNC and KPWA tend to provide all care for their patients, we should be able to capture full healthcare utilization from their EHRs and price it using Medicare rates. SH provides most of their participants' care and we will explore whether we can use a similar pricing scheme for their participants. However, IFH only provides primary care. Therefore, the IFH EHR will only contain information on primary care-related healthcare utilization and incomplete information on referrals to specialists and hospitalizations. We will be limited to the data we can obtain in a timely manner from Medicare for IFH participants.

A bootstrap methodology will be used to estimate confidence intervals,[111,112] and one-way sensitivity analyses will be performed to determine the robustness of our estimates with different assumptions such as the reimbursement rate for acupuncture and the inclusion of back-pain related costs only.[106]

The cost-effectiveness analysis will be conducted by Dr. Patricia Herman with assistance from Dr. Samuel Mann at RAND in Santa Monica, California using de-identified data obtained from the Coordinating Center.

<u>Aim 3</u> (Qualitative Analyses): All interviews will be audio recorded. and the participant and stakeholder interviews will be transcribed by a professional transcriptionist; the acupuncturist interviews will be automatically transcribed by Teams and then reviewed by study team members. Focus groups with patients and acupuncturists will be transcribed in real time by a court reporter (IFH, KPNC and KPWA) or by a transcription service after-the-fact. Coding will be completed by trained coders using ATLAS.ti, a qualitative analysis software program that aids management and interpretation of text-based and other non-quantitative data. Coder reliability will be ensured through using an iterative process of coding the same text and comparing codes and discussing discrepancies. Code definitions will be updated as needed to ensure clarity.

Once the interviews are coded for each phase of the study (see Section 6.2.2. Aim 3 data collection), we will use ATLAS.ti queries to produce reports of text associated with primary codes and begin synthesizing themes from this text. ATLAS.ti allows retrieval of coded information in multiple ways, including by participant features (e.g., gender, pain diagnoses) by a code alone, or by combinations of co-occurring codes. This approach to coding and data reduction will allow us to examine issues from multiple perspectives and ensures a thorough review of the data increase the breadth and depth of insights generate from the qualitative data gathered.

10. DATA COLLECTION AND QUALITY ASSURANCE

10.1 Data Collection Forms

Staff at KPWHRI will develop the oral eligibility screening language and the baseline, monthly, 3, 6 and 12-month follow-up questionnaires. After consent, all sites will transfer data on participants to the centralized data capture system, Discovery, at KPWHRI. Data collection at baseline will be conducted by site staff using Discovery. KPWHRI Survey Research Program will do all follow-up assessments. These will be done by computer assisted telephone interviewing (CATI at 3-, 6-,12-month follow-ups), web (3-, 6-,12-month follow-up and monthly) or both, depending on the needs to each participant. These data will be considered primary source data. All interviewers will be masked to treatment group.

10.2 Data Management

A detailed data-quality management program including specific protocols for data collection and quality control will be developed by our KPWHRI Data Coordinating Center (DCC)[see Section 12] with input from pertinent staff across our performance sites during the UG3 phase.

For this study, Kaiser Permanente Washington Health Research Institute (KPWHRI) will serve as the Data Coordinating Center (DCC). Individual sites will be responsible for participant tracking throughout the pre-consent phase, and after consent, all primary data collection will be conducted using **Discovery**: DatStat's Research Management System (DD-RMS). DD-RMS is a self-hosted module of DatStat housed on servers at KPWA. All data input into the system are stored in a database server located behind the organizational firewall, with limited access between web server and database server. DD-RMS is built on the .Net platform, offers an open architecture and well-documented API, and is designed to streamline study management, participant management, data capture, aggregation, and reporting within and across complex research studies. As a web-based application, the DD-RMS provides a single, secure point of access and supports centralized studies and multi-site collaborative research efforts. DD-RMS has built-in flexibility to support the range of healthcare system-specific study workflows necessary during the process of participant identification and recruitment, including provisions for role-based access and privileges such that only local HCS staff can access patient health information prior to consenting when participant permissions are given that will allow for the KPWHRI DCC staff to manage all patient data for follow-up assessments and other linkage needs. The DD-RMS includes functionality for survey form development and data capture, management, and quality control. The system is designed to track most all study related information and provides a single point of access for all users (research coordinators, interviewers, and study patient participants. (Acupuncture treatment data will be collected in Epic at IFH and in another database, described below, for the other sites.). The DD-RMS supports multimodalities for data collection including web-based data form completion, telephone interviewer form support, and batched paper form entry. The system records and

tracks all study contacts (e.g., calls, visits, emails, mailings) and associated outcome status as well as having the functionality to manage sampling and random assignment. DD-RMS has been approved by KPWA's Institutional Review Board as a tool that can be used for secure collection and storage of data, including PII/PHI.

The DD-RMS will be the coordinating center's primary electronic data capture and management system and used exclusively to house and manage patient and intervention-related study data ***after*** participants are consented for the study. However, regulatory, compliance, and information-security technology reviews (TRO, SRA, etc.) are required by all participating health care systems prior to housing patient data. As this is a lengthy process and is still underway at the writing of this report, we have planned for back-up EDC and management systems at each of our satellite performance sites (KPNC, SH, and IFH) to manage patient data within the firewalls of the patients HCS prior to consent as described below.

Individual Sites
KPNC: For pre-consent data tracking, programmers at KPNC will build a recruiting tracking system utilizing Microsoft Office 365 Access. Standard procedures for tracking recruitment of trial participants will be employed.
SH: For pre-consent data tracking, programmers at SH will build a recruiting tracking system utilizing Microsoft Access 2013. Standard procedures for tracking recruitment of trial participants will be employed.
IFH: For pre-consent data tracking, programmers at IFH will build a recruiting tracking system utilizing REDCap (version 9.9.2). Standard procedures for tracking recruitment of trial participants will be employed.

All our performance sites (KPWA, KPNC, SH, KPNW, and IFH) are HIPAA-covered entities and comply with all HIPAA regulations regarding data security. All study files maintained at any of our affiliated research institutes will be maintained either behind secure firewalls on system network drives, in a centralized location on the institute servers or in locked file cabinets. Access to these data will be password protected and subject to the same security protections as other confidential health plan data. Access will be limited to staff working on this study who require access to these files. Whenever possible, study data will be stored on our central study DatStat tracking system. All staff at our participating sites are trained in appropriate security protections, computer passwords are changed on a regular basis, and all staff sign annual confidentiality agreements. Data transferred from sites to KPWHRI will be done via a web-based secure file transfer (SFT) application, which uses the 128-bit Secure Sockets Layer encryption protocol and meets the 2009 HIPAA HITECH safe harbor standard. This method is commonly used in our multi-site studies and has been reviewed and approved by our IRBs. No data or identifiable information will be stored on participants' phones or devices. No sensitive information will be shared in texts or emails with participants. The DCC at KPWA will securely transfer identifiable datasets and de-identified datasets via the SFT application to study sites (KPNC, Sutter, IFH, KPWA, KPNW) for analyses when applicable data sharing agreements are in place.

Clinical sites will be responsible for recruitment (outreach, assessment of eligibility, consent, baseline data collection and randomization) and connecting the patients to acupuncturists. By using centralized databases, we will be able to have standardized reports to the extent possible. The DCC will prepare the randomization schemes and insert them into the proper location in the computer program for the baseline interviews. The Survey Research Program at KPWHRI will conduct all follow-up interviews and outreach. The study sites will manage all acupuncture visits. Treatment data will be collected in Epic (IFH) or a study-specific data base for the

acupuncturists. KPWHRI staff will monitor progress, look for outliers in various treatment parameters and inform the acupuncture training team and site staff of any issues they detect. Section 10.3.5 describes monitoring in more detail.

KPWHRI will also build a password-protected HIPAA compliant SQL server database with a web-interface that contains the treatment data from all acupuncturists (KPNC, KPWA, SH) who do not chart in Epic (IFH). It will contain appropriate range and logic checks as well. Participants will be tracked by study identifier in this database.

10.3 Quality Assurance

10.3.1 Training

Protection of Human Subjects Training: All study personnel, including investigators, staff, and study acupuncturists, will complete the required training on the protection of human subjects before they engage in any human subjects activity. They will recertify on the schedule required by their institution.

Training of Study Site Coordinators: The site study coordinators will meet virtually for a training that will be developed during July of the UG3 year. Training will begin with a review of our study documentation (e.g., study protocol, manual of procedures, consents, questionnaires). We will also discuss communication with patients, acupuncturists and physicians. We will describe our database and data management, including our agreed upon procedures. We will have role playing as appropriate. We will incorporate cultural competency training in this training process. We will have ongoing training for quality assurance every six months during site visits or internal audits with the project manager.

Training of Acupuncturists: During the UG3 phase of the study, we developed materials to orient the acupuncturists to the cLBP trial specifics including the study protocol, number of sessions in each arm, the parameters of the consensus intervention protocol, minimum number of sessions recommended, when to discontinue treatment for failure to respond, compliance on recording a session using the trial treatment forms, tracking and reporting adverse events, procedures for communicating with participating patients' PCP. We developed procedures for acupuncturists to ask questions as issues come up and in turn how we will communicate that information to all study acupuncturists. Additionally, a review of harms risks will be required including safe needling practices and appropriate adaptations for older adults. Finally, each site will train their acupuncturists on site-specific procedures for getting paid and any other site-specific procedures (e.g., who to contact to report a concerning adverse event. Acupuncturist trial training will be presented in webinar format, using PowerPoint and recorded so it is accessible to acupuncturists for re-viewing and to new study acupuncturists for training throughout the treatment period. PDFs of material will be included in the training packet as well as a short quiz of the safety presentation. Materials on safety were developed by Dr. Arya Nielsen based on an existing NCCAOM accredited webinar on acupuncture therapy risks and harms.[113] After completing our training as well as a quiz covering the key components of the treatment and safety, acupuncturists will be certified for participation in the study.

Training of Interviewers: The Survey Research Program at KPWA has a rigorous foundational training program for all Research Interviewers. In addition, Interviewers train for specific studies, growing familiar with study goals, protocols and recruitment and survey instruments. Study training involves multiple learning activities to ensure that Interviewers are well prepared to engage with study participants. Typical training includes an introduction from the study

investigator and project manager, review of study materials including advance letters, brochures/information sheets, consent forms, as well as time dedicated to practice and role play study scripts. Training materials include a quick reference guide of recommended responses for known or anticipated frequently asked questions. Post training, Interviewers debrief their learnings, noting any questions and sharing their experiences with the survey instruments.

Research Interviewers conduct interviews and administer surveys via Computer Assisted Telephone Interviewing. CATI programming is tested for range and logic for each variable. The Survey Research Program will work with all sites and share resources to ensure that all recruiters and baseline interviewers have comparable training. Cultural competency training will be incorporated in the interviewer training.

Training of Qualitative Interviewers: Dr Clarissa Hsu or another team member with expertise in qualitative methods will ensure that all the qualitative interviewers have been appropriately trained so that the qualitative data collection optimizes resources and efficiently addresses study activities essential to Aim 3. This will include ensuring that all qualitative interviewers have sufficient training in qualitative interviewing and focus group facilitation and that they understand the intent of all questions to be able to probe and guide conversation effectively and efficiently. Dr. Hsu and other qualitative methods experts will organize oversight of study qualitative activities by assessing the training and experience of each person participating in qualitative data collection via a phone conversation, reviewing any interview/facilitation guides with qualitative team members and having individuals observe one or more focus groups/interviews facilitated by Dr. Hsu or another experienced team member followed by a group debrief session.

10.3.2 Quality Control Committee

The Study PI's, site PI's, a biostatistician, and other Co-I's and senior study staff as needed, will meet weekly (or bi-monthly if the study is recruiting without difficulty) to keep abreast of the day-to-day operations at each performance site and help if needed. This group will discuss the progress of the trial, recruitment and any safety or IRB concerns.

10.3.3 Metrics

We will collect the information necessary to ensure that we can report trial flow according to the CONSORT diagram.[114] Reports will include the number of referrals, letters sent out, number of contacts to study, number of phone screens and outcomes, and other elements of the enrollment process. Less frequently, we will obtain data on age group, gender, race and ethnicity and summaries of completed acupuncture treatments. For each monthly follow-up survey, we aim to achieve an 85% or higher follow-up rate. We will utilize a combination of web-based survey and telephone outreach to achieve maximal survey response – especially at the main 3-, 6- and 12-month follow-up time points. Reminder emails, letters or phone calls – depending on each participant's needs - will be provided the week before the surveys are due. Survey completion rates will be primarily based upon the completion of functional outcome and pain-related measures, but our data management team at KPWHRI will additionally tabulate follow-up by each instrument to monitor and evaluate survey burden. We will also create reports for the acupuncture treatments to monitor adherence to the intervention protocol. We anticipate that there will be other metrics we will use that will become clear to us as we delve more deeply into the database build and after discussion of our study with the Protocol Review Committee.

10.3.4 Protocol Deviations

We define a protocol deviation as any lack of compliance with the study protocol, manual of procedures or other approved study procedures that does not result in increased risk to the participant or integrity of the study. For example, failure to obtain all follow-up data from a participant. We will define a protocol violation as any lack of compliance with the study protocol, manual of procedures or other approved study procedures that could increase risk to the participant or integrity of the study. For example, enrolling a participant who does not meet the inclusion and exclusion criteria. Protocol deviation and violations will be tracked prospectively, captured in real time and reviewed at regularly scheduled full study team meeting as well as logged for IRB and Independent Monitoring Committee review as required.

10.3.5 Monitoring

We will implement procedures to review electronic consent forms, signed acupuncture consent forms as well as documentation of oral consent. For data captured electronically, we will generate summary reports on a weekly basis to review the recruitment process and conduct of the trial. Reports and findings will be shared across sites and the programmers from all sites will contribute to the finalized, curated code to ensure adequate recognition of site-specific issues and consistency in operation, implementation and documentation. Evaluation and monitoring will also be conducted via site visits or structured internal audits, where specific issues can be addressed and remediated.

The data manager from our KPWHRI survey department will run and review data quality and missing data reports on a weekly basis for the follow-up interviews. Free text data collection from study participants will be minimized to the extent possible and field masking and automated out of range checks will be implemented where applicable.

We will run reports from the treatment data collected by the acupuncturists to ensure that they are adhering to the protocol. These processes will be described in the Manual of Procedures and details will be fleshed out during the last several months of the UG3 year and the first month of the UH3 year.

11. PARTICIPANT RIGHTS AND CONFIDENTIALITY

11.1 Institutional Review Board (IRB) Review

This protocol, the informed consent documents (Appendix 3), and any subsequent modifications will be reviewed and approved by the KPNC IRB, who will be responsible for overall oversight of the UH3 clinical trial (sIRB of record). The consent form should be separate from the protocol document. The Institute for Family Health, KPWA and SH will yield to the KPNC IRB. The KPNC IRB will be apprised of any differences in state laws and customs of the other health care institutions that may require customization of consent forms for each performance site. The sIRB may require different procedures for some of the processes we have described (e.g., adverse event monitoring). If so, those changes would be made.

11.2 Informed Consent Forms

All sites will ask for a waiver of written consent to screen prospective participants using a structured eligibility screener.

FOR HCSs PERMITTING ORAL CONSENT

In addition, at some sites, individuals will have received a consent checklist that carefully lays out the elements of informed consent and HIPAA. The interviewers will make sure the participant has the checklist in front of him/her and will then review each element, make sure that prospective participants understand each element and are comfortable providing oral informed consent. This will be documented in the computer assisted interviewing program with date, time, the interviewers name and the participants name.

FOR HCSs REQUIRING WRITTEN CONSENT

An electronic signed consent form will be obtained from each participant. The consent form will include all necessary elements of informed consent including a description of the purpose of the study, the procedures to be followed, and the risks and benefits of participation. They will be able to print a signed copy for their records, and this fact will be documented in the participant's study record.

11.3 Participant Confidentiality

We will maintain strict patient confidentiality through several means. All study staff (including the acupuncturists) are required to complete training regarding principles and procedures for protecting the confidentiality of health information. Only staff who need to see particular data will be given access to those files. All information needed to recruit patients (name, address, phone number) will be stored in password protected databases (with strong passwords with timeouts and behind the firewalls of each HCS) and/or locked file cabinets. At the time of enrollment, participants will be assigned a unique study identifier (SID). After participants are enrolled and baseline data collected, their minimum-necessary contact information and pertinent electronic health record data will be securely transferred to the Survey Research Program at the Kaiser Permanente Washington Health Research Institute Data transferred from sites to KPWHRI (via a web-based secure file transfer (SFT) application; see section 10.2) for all follow-up interviews. Data will be stored securely in DatStat. Contact information will be needed to collect follow-up data but will be stored in a password protected database behind the KPWHRI firewall and will be available only to personnel who need to collect the follow-up data. The database will have multiple levels of permissions so that only those needing the contact information will have access to it. Language to explain the process will be included in the consent form so that participants will be informed.

Any data files created for research use will include no identifying information (name, health plan number, birth date) and records will be identified only by SID. Only site study programmers will have access to the crosswalk linking SIDs to health plan member numbers.

Acupuncturists will complete electronic visit forms, but they will only include the participant's ID number, dates and other details of the treatment so it will not be possible to identify individuals. If we have a need to transmit information about a patient to the acupuncturist, we will call their office.

Paper records will be kept in locked filing cabinets in secure research areas. Data collected at follow-up time points will be stored by ID number only. Information will not be released without the express consent of the participants. Any study participant information stored temporarily at an acupuncturist's office while the participant is undergoing treatment will be stored using HIPAA-compliant procedures.

Any data, forms, reports, audio recordings, and other records that leave the site will be identified only by a study identification number (SID) to maintain confidentiality. All records will be kept in a locked file cabinet. All computer entry and networking programs will be done using SIDs only. Information will not be released without written permission of the participant, except as necessary for monitoring by IRB, the NCCIH, and the OHRP.

11.4 Study Discontinuation

The study may be discontinued at any time by the IRB, the NCCIH, the OHRP or other government agencies as part of their duties to ensure that research participants are protected.

12. COMMITTEES

Study Principal Investigators and Core Executive Committee (CEC)

Drs. Lynn DeBar and Andrea Cook will serve as MPIs on this project. They will be responsible for implementation of the Specific Aims and will ensure that systems are in place to guarantee institutional compliance with US laws and federal policies including human research, data and facilities. They will be assisted by the Core Executive Committee (CEC), which will include the site- PI’s at IFH, KPNC and Sutter, the Statistical Methods Committee (SMC), which will include the biostatisticians and cost-effectiveness expert, other project content experts (in geriatrics, acupuncture, qualitative methods). The CEC will meet regularly to handle any major study decisions regarding the direction of scientific aims, allocation of resources, disputes that may arise, and other information related to the management of the proposed project. As appropriate, they will receive guidance from the other investigators on the team, consultants and study staff. Major study decisions will be made by vote of the CEC. The odd number of core executive team members eliminates the chance of a tie.

Statistical Methods Committee (SMC)

Dr. Andrea Cook will chair the SMC, which will interface with the NIH Collaboratory Biostatistics and Study Design core. The SMC will include Mr. Wellman and Dr. DeBar as ex-officio members. Dr. Herman will join the SMC for analytic strategies related to Aim 2. The SMC will refine the overarching analytic strategy and if additional scientific input is needed, reach out to the CEC. The CEC is responsible for all final decisions about the scientific aims, allocation of resources for statistical investigations, and resolution of disputes will be made by the CEC. Members will also serve as project liaisons to the NIH Collaboratory Working Groups.

NIH Leadership

Dr. Lanay Mudd will serve as the Program Officer from NCCIH and Drs. Robin Boineau from NCCIH (December 2019 to February 2022) and Basil Eldadah from NIA will be the Project Scientists. They will meet regularly with Drs. Cook and DeBar and will provide oversight and advice. As needed, other members of the scientific or study staff will join these meetings.

Data Coordinating Center (DCC)

Drs. DeBar and Cook in collaboration with the SMC will oversee the Data Coordinating Center for the study. KPWHRI was chosen as the Data Coordinating Center because there is an existing infrastructure that has been established through the KPWHRI Survey Research Program that will support screening, baseline, monthly check in and 3-, 6-, and 12-month follow

up assessments. Drs. DeBar and Cook each have substantial experience in multi-site collaborations involving both primary data collection from patients and secondary data collection from electronic medical records. However, they will remain masked to treatment outcomes until the data base is finalized and locked.

Independent Monitoring Committee

Approved by NCCIH, the IMC plays a crucial role in ensuring the safety and welfare of patients enrolled in this trial, and operates without undue influence from any interested party, including BIA study investigators or NCCIH staff. IMC responsibilities include protocol approval, interim review of trial enrollment, protocol compliance, and safety data. The protocol review committee is comprised of the same members as the IMC and is referred to such during the UG3 preparatory phase of the study.

13. PUBLICATION OF RESEARCH FINDINGS

Publication of the results of this trial will be governed by the policies and procedures developed by the CEC. Any presentation, abstract, or manuscript will be made available for review by the NCCIH and NIH Collaboratory Publications Committee prior to submission.

14. DATA SHARING

This study will comply with all applicable NIH Data Sharing Policies. (See https://grants.nih.gov/policy/sharing.htm for policies and resources)

The DCC will produce a public/releasable database from the study. The releasable database will be completely de-identified in accordance with the definitions provided in the Health Insurance Portability and Accountability Act (HIPAA). Namely, all identifiers specified in HIPAA will be recoded in a manner that will make it impossible to deduce or impute the specific identity of any patient. The database will not contain any institutional identifiers.

The DCC will also prepare a data dictionary that provides a concise definition of every data element included in the database. If specific data elements have idiosyncrasies that might affect interpretation or analysis, this will be discussed in the dictionary document. Data elements that are considered unreliable will be deleted, and this will be noted in the documentation.

The policies for release of this database will be determined by the NIH. These policies are expected to focus primarily on the timing of data release. The preliminary plan is to release the database at the time of publication of the primary manuscript. Implementation of the plan will follow the HEAL Public Access and Data Sharing Policy as outlined at https://heal.nih.gov/about/public-access-data.

In accordance with policies of the NIH, the DCC will send the releasable database and its relevant documentation to the entity determined by the NIH or specific institute to be the repository for data created under the HEAL initiative.

Access to the releasable database housed in the NIH-assigned repository will be in accordance with procedures and regulations of the NIH or specific institute.

14. REFERENCES

1. Hoy D, March L, Brooks P, et al. The global burden of low back pain: estimates from the Global Burden of Disease 2010 study. *Ann Rheum Dis.* 2014;73(6):968-974.
2. Martin BI, Deyo RA, Mirza SK, et al. Expenditures and health status among adults with back and neck problems. *JAMA.* 2008;299(6):656-664.
3. Weiner DK, Rudy TE, Morrow L, Slaboda J, Lieber S. The relationship between pain, neuropsychological performance, and physical function in community-dwelling older adults with chronic low back pain. *Pain Med.* 2006;7(1):60-70.
4. Deyo RA, Mirza SK, Turner JA, Martin BI. Overtreating chronic back pain: time to back off? *J Am Board Fam Med.* 2009;22(1):62-68.
5. Docking RE, Fleming J, Brayne C, et al. Epidemiology of back pain in older adults: prevalence and risk factors for back pain onset. *Rheumatology (Oxford).* 2011;50(9):1645-1653.
6. Rundell SD, Sherman KJ, Heagerty PJ, Mock CN, Jarvik JG. The clinical course of pain and function in older adults with a new primary care visit for back pain. *J Am Geriatr Soc.* 2015;63(3):524-530.
7. Arnstein P. Balancing analgesic efficacy with safety concerns in the older patient. *Pain Manag Nurs.* 2010;11(2 Suppl):S11-22.
8. Weiner DK. Deconstructing chronic low back pain in the older adult: shifting the paradigm from the spine to the person. *Pain Med.* 2015;16(5):881-885.
9. Makris UE, Abrams RC, Gurland B, Reid MC. Management of persistent pain in the older patient: a clinical review. *JAMA.* 2014;312(8):825-836.
10. Jarvik JG, Gold LS, Comstock BA, et al. Association of early imaging for back pain with clinical outcomes in older adults. *JAMA.* 2015;313(11):1143-1153.
11. Barkin RL, Beckerman M, Blum SL, Clark FM, Koh EK, Wu DS. Should nonsteroidal anti-inflammatory drugs (NSAIDs) be prescribed to the older adult? *Drugs Aging.* 2010;27(10):775-789.
12. Cooper JW, Burfield AH. Assessment and management of chronic pain in the older adult. *J Am Pharm Assoc (2003).* 2010;50(3):e89-99; quiz e100-101.
13. Fitzcharles MA, Lussier D, Shir Y. Management of chronic arthritis pain in the elderly. *Drugs Aging.* 2010;27(6):471-490.
14. Institute of Medicine (US) Committee on Advancing Pain Research Care and Education. *Relieving pain in america: a blueprint for transforming prevention, care, education, and research.* Washington (DC): National Academies Press; 2011.
15. Interagency Pain Research Coordinating Committee. *National pain strategy: a comprehensive population health-level strategy by pain.* 2015.
16. Reid MC, Ong AD, Henderson CR, Jr. Why we need nonpharmacologic approaches to manage chronic low back pain in older adults. *JAMA Intern Med.* 2016;176(3):338-339.

17. Townley S, Papaleontiou M, Amanfo L, et al. Preparing to implement a self-management program for back pain in new york city senior centers: what do prospective consumers think? *Pain Med.* 2010;11(3):405-415.
18. Nascimento P, Costa LOP, Araujo AC, Poitras S, Bilodeau M. Effectiveness of interventions for non-specific low back pain in older adults. a systematic review and meta-analysis. *Physiotherapy.* 2018.
19. Qaseem A, Wilt TJ, McLean RM, Forciea MA, Clinical Guidelines Committee of the American College of Physicians. Noninvasive treatments for acute, subacute, and chronic low back pain: a clinical practice guideline from the american college of physicians. *Ann Intern Med.* 2017;166(7):514-530.
20. Chou R, Deyo R, Friedly J, et al. Nonpharmacologic therapies for low back pain: a systematic review for an american college of physicians clinical practice guideline. *Ann Intern Med.* 2017;166(7):493-505.
21. Turk DC, Dworkin RH, Revicki D, et al. Identifying important outcome domains for chronic pain clinical trials: an IMMPACT survey of people with pain. *Pain.* 2008;137(2):276-285.
22. Vickers AJ, Vertosick EA, Lewith G, et al. Acupuncture for chronic pain: update of an individual patient data meta-analysis. *J Pain.* 2018;19(5):455-474.
23. Cherkin DC, Eisenberg D, Sherman KJ, et al. Randomized trial comparing traditional Chinese medical acupuncture, therapeutic massage, and self-care education for chronic low back pain. *Arch Intern Med.* 2001;161(8):1081-1088.
24. Thomas KJ, MacPherson H, Thorpe L, et al. Randomised controlled trial of a short course of traditional acupuncture compared with usual care for persistent non-specific low back pain. *BMJ.* 2006;333(7569):623.
25. Cherkin DC, Sherman KJ, Avins AL, et al. A randomized controlled trial comparing acupuncture, simulated acupuncture, and usual care for chronic low back pain. *Arch Intern Med.* 2009;169(9):858-866.
26. Molsberger AF, Mau J, Pawelec DB, Winkler J. Does acupuncture improve the orthopedic management of chronic low back pain - a randomized, blinded, controlled trial with 3 months follow up. *Pain.* 2002;99(3):579-587.
27. Brinkhaus B, Witt CM, Jena S, et al. Acupuncture in patients with chronic low back pain: a randomized controlled trial. *Arch Intern Med.* 2006;166(4):450-457.
28. Weidenhammer W, Linde K, Streng A, Hoppe A, Melchart D. Acupuncture for chronic low back pain in routine care: a multicenter observational study. *Clin J Pain.* 2007;23(2):128-135.
29. Haake M, Muller HH, Schade-Brittinger C, et al. German Acupuncture Trials (GERAC) for chronic low back pain: randomized, multicenter, blinded, parallel-group trial with 3 groups. *Arch Intern Med.* 2007;167(17):1892-1898.
30. Witt CM, Jena S, Selim D, et al. Pragmatic randomized trial evaluating the clinical and economic effectiveness of acupuncture for chronic low back pain. *Am J Epidemiol.* 2006.

31. Liu L, Skinner MA, McDonough SM, Baxter GD. Acupuncture for chronic low back pain: a randomized controlled feasibility trial comparing treatment session numbers. *Clin Rehabil.* 2017;31(12):1592-1603.
32. McKee MD, Nielsen A, Anderson B, et al. Individual vs. Group Delivery of Acupuncture Therapy for Chronic Musculoskeletal Pain in Urban Primary Care – A Randomized Trial. *J Gen Intern Med.* 2020;in press.
33. Macpherson H, Scullion A, Thomas KJ, Walters S. Patient reports of adverse events associated with acupuncture treatment: a prospective national survey. *Qual Saf Health Care.* 2004;13(5):349-355.
34. Witt CM, Pach D, Brinkhaus B, et al. Safety of acupuncture: results of a prospective observational study with 229,230 patients and introduction of a medical information and consent form. *Forsch Komplementmed.* 2009;16(2):91-97.
35. MacPherson H. How safe is acupuncture? Developing the evidence on risk. *J Altern Complement Med.* 1999;5(3):223-224.
36. White A, Hayhoe S, Hart A, Ernst E. Adverse events following acupuncture: prospective survey of 32 000 consultations with doctors and physiotherapists. *BMJ.* 2001;323(7311):485-486.
37. Hopton AK, Thomas KJ, MacPherson H. Willingness to try acupuncture again: reports from patients on their treatment reactions in a low back pain trial. *Acupunct Med.* 2010;28(4):185-188.
38. Jensen TSC, J.; Evans, MA, et al. National Coverage Determination for Acupuncture for Chronic Low Back Pain. In: Coverage and Analysis Group CfMMS, ed: US Government, CMS; 2020.
39. Rhee TG, Marottoli RA, Van Ness PH, Tinetti ME. Patterns and perceived benefits of utilizing seven major complementary health approaches in U.S. older adults. *J Gerontol A Biol Sci Med Sci.* 2018;73(8):1119-1124.
40. Ratcliffe J, Thomas KJ, MacPherson H, Brazier J. A randomised controlled trial of acupuncture care for persistent low back pain: cost effectiveness analysis. *BMJ.* 2006 Sep 23;333(7569):626.
41. Cherkin DC, Sherman KJ, Kahn J, et al. A comparison of the effects of 2 types of massage and usual care on chronic low back pain: a randomized, controlled trial. *Ann Intern Med.* 2011;155(1):1-9.
42. Ezzo J, Berman B, Hadhazy VA, Jadad AR, Lao L, Singh BB. Is acupuncture effective for the treatment of chronic pain? A systematic review. *Pain.* 2000;86(3.):217-225.
43. Sherman KJ, Cherkin DC, Deyo RA, et al. The diagnosis and treatment of chronic back pain by acupuncturists, chiropractors, and massage therapists. *Clin J Pain.* 2006;22(3):227-234.
44. Nielsen A, Anderson B, Citkovitz C, et al. Developing and employing a 'responsive manualization' in the 'Acupuncture Approaches to Decrease Disparities in Outcomes of Pain Treatment' comparative effectiveness study. *Acupunct Med.* 2019;37(3):184-191.
45. Brinkhaus B, Witt CM, Jena S, et al. Acupuncture in patients with chronic low back pain: a randomized controlled trial. *Arch Intern Med.* 2006 Feb 27;166(4):450-457.

46. Grant DJ, Bishop-Miller J, Winchester DM, Anderson M, Faulkner S. A randomized comparative trial of acupuncture versus transcutaneous electrical nerve stimulation for chronic back pain in the elderly. *Pain.* 1999;82(1):9-13.
47. MacPherson H, Thorpe L, Thomas K, Campbell M. Acupuncture for low back pain: traditional diagnosis and treatment of 148 patients in a clinical trial. *Complement Ther Med.* 2004;12(1):38-44.
48. Sherman KJ, Cherkin DC, Hogeboom CJ. The diagnosis and treatment of patients with chronic low-back pain by traditional Chinese medical acupuncturists. *J Altern Complement Med.* 2001;7(6):641-650.
49. Carlsson CP, Sjolund BH. Acupuncture for chronic low back pain: a randomized placebo-controlled study with long-term follow-up. *Clin J Pain.* 2001;17(4):296-305.
50. Comachio J, Oliveira Magalhaes M, Nogueira Burke T, et al. Efficacy of acupuncture and electroacupuncture in patients with nonspecific low back pain: study protocol for a randomized controlled trial. *Trials.* 2015;16:469.
51. Kerr DP, Walsh DM, Baxter D. Acupuncture in the management of chronic low back pain: a blinded randomized controlled trial. *Clin J Pain.* 2003;19(6):364-370.
52. Kong JT, MacIsaac B, Cogan R, et al. Central mechanisms of real and sham electroacupuncture in the treatment of chronic low back pain: study protocol for a randomized, placebo-controlled clinical trial. *Trials.* 2018;19(1):685.
53. Fan AY, Ouyang H, Qian X, et al. Discussions on real-world acupuncture treatments for chronic low-back pain in older adults. *J Integr Med.* 2019;17(2):71-76.
54. MacPherson H, Altman DG, Hammerschlag R, et al. Revised STandards for Reporting Interventions in Clinical Trials of Acupuncture (STRICTA): extending the CONSORT statement. *PLoS Med.* 2010;7(6):e1000261.
55. Sherman KJ, Hogeboom CJ, Cherkin DC. How traditinal Chinese medicine acupuncturists would diagnose and treat chronic low back pain: results of a survey of licensed acupuncturists in Washington State. *Complement Ther Med.* 2001;9:146-153.
56. Berman BM, Lao L, Langenberg P, Lee WL, Gilpin AM, Hochberg MC. Effectiveness of acupuncture as adjunctive therapy in osteoarthritis of the knee: a randomized, controlled trial. *Ann Intern Med.* 2004;141(12):901-910.
57. DeBar LL, Elder C, Ritenbaugh C, et al. Acupuncture and chiropractic care for chronic pain in an integrated health plan: a mixed methods study. *BMC Complement Altern Med.* 2011;11:118.
58. Sherman KJ, Cherkin DC, Eisenberg DM, Erro J, Hrbek A, Deyo RA. The practice of acupuncture: who are the providers and what do they do? *Ann Fam Med.* 2005;3(2):151-158.
59. Working Group on Health Outcomes for Older Persons with Multiple Chronic Conditions. Universal health outcome measures for older persons with multiple chronic conditions. *J Am Geriatr Soc.* 2012;60(12):2333-2341.

60. Deyo RA, Dworkin RH, Amtmann D, et al. *Report of the Task Force on Research Standards for Chronic Low-Back Pain.* NIH;2013.
61. Dworkin RH, Turk DC, Farrar JT, et al. Core outcome measures for chronic pain clinical trials: IMMPACT recommendations. *Pain.* 2005;113(1-2):9-19.
62. Jones SM, Lange J, Turner J, et al. Development and Validation of the EXPECT Questionnaire: Assessing Patient Expectations of Outcomes of Complementary and Alternative Medicine Treatments for Chronic Pain. *J Altern Complement Med.* 2016;22(11):936-946.
63. Coleman BC, Kean J, Brandt CA, Peduzzi P, Kerns RD. Adapting to disruption of research during the COVID-19 pandemic while testing nonpharmacological approaches to pain management. *Transl Behav Med.* 2020;10(4):827-834.
64. Roland M, Fairbank J. The Roland-Morris Disability Questionnaire and the Oswestry Disability Questionnaire. *Spine.* 2000;25(24):3115-3124.
65. Williams K, Abildso C, Steinberg L, et al. Evaluation of the effectiveness and efficacy of iyengar yoga therapy on chronic low back pain. *Spine (Phila Pa 1976).* 2009;34(19):2066-2076.
66. Vlaeyen JW, Morley S. Cognitive-behavioral treatments for chronic pain: what works for whom? *Clin J Pain.* 2005;21(1):1-8.
67. Cook AJ, Brawer PA, Vowles KE. The fear-avoidance model of chronic pain: validation and age analysis using structural equation modeling. *Pain.* 2006;121(3):195-206.
68. Goldsmith ES, Taylor BC, Greer N, et al. Focused Evidence Review: Psychometric Properties of Patient-Reported Outcome Measures for Chronic Musculoskeletal Pain. *J Gen Intern Med.* 2018;33(Suppl 1):61-70.
69. Chapman JR, Norvell DC, Hermsmeyer JT, et al. Evaluating common outcomes for measuring treatment success for chronic low back pain. *Spine (Phila Pa 1976).* 2011;36(21 Suppl):S54-68.
70. Chou R, Qaseem A, Snow V, et al. Diagnosis and treatment of low back pain: a joint clinical practice guideline from the American College of Physicians and the American Pain Society. *Ann Intern Med.* 2007;147(7):478-491.
71. Bombardier C, Hayden J, Beaton DE. Minimal clinically important difference. Low back pain: outcome measures. *J Rheumatol.* 2001;28(2):431-438.
72. Ostelo RW, Deyo RA, Stratford P, et al. Interpreting change scores for pain and functional status in low back pain: towards international consensus regarding minimal important change. *Spine.* 2008;33(1):90-94.
73. Deyo RA, Dworkin SF, Amtmann D, et al. Report of the NIH Task Force on research standards for chronic low back pain. *Phys Ther.* 2015;95(2):e1-e18.
74. Farrar JT, Young JP, Jr., LaMoreaux L, Werth JL, Poole RM. Clinical importance of changes in chronic pain intensity measured on an 11-point numerical pain rating scale. *Pain.* 2001;94(2):149-158.

75. Krebs EE, Lorenz KA, Bair MJ, et al. Development and initial validation of the PEG, a three-item scale assessing pain intensity and interference. *J Gen Intern Med.* 2009;24(6):733-738.
76. Cella D, Riley W, Stone A, et al. The Patient-Reported Outcomes Measurement Information System (PROMIS) developed and tested its first wave of adult self-reported health outcome item banks: 2005-2008. *J Clin Epidemiol.* 2010;63(11):1179-1194.
77. Gershon RC, Rothrock N, Hanrahan R, Bass M, Cella D. The use of PROMIS and assessment center to deliver patient-reported outcome measures in clinical research. *J Appl Meas.* 2010;11(3):304-314.
78. Deyo RA, Katrina R, Buckley DI, et al. Performance of a Patient Reported Outcomes Measurement Information System (PROMIS) Short Form in Older Adults with Chronic Musculoskeletal Pain. *Pain Med.* 2016;17(2):314-324.
79. Huang W, Rose AJ, Bayliss E, et al. Adapting summary scores for the PROMIS-29 v2.0 for use among older adults with multiple chronic conditions. *Qual Life Res.* 2019;28(1):199-210.
80. Cella D, Choi SW, Condon DM, et al. PROMIS((R)) Adult Health Profiles: Efficient Short-Form Measures of Seven Health Domains. *Value Health.* 2019;22(5):537-544.
81. Segawa E, Schalet B, Cella D. A comparison of computer adaptive tests (CATs) and short forms in terms of accuracy and number of items administrated using PROMIS profile. *Qual Life Res.* 2019.
82. Chen CX, Kroenke K, Stump T, et al. Comparative Responsiveness of the PROMIS Pain Interference Short Forms With Legacy Pain Measures: Results From Three Randomized Clinical Trials. *J Pain.* 2019;20(6):664-675.
83. Chen CX, Kroenke K, Stump TE, et al. Estimating minimally important differences for the PROMIS pain interference scales: results from 3 randomized clinical trials. *Pain.* 2018;159(4):775-782.
84. Chiarotto A, Roorda LD, Crins MH, Boers M, Ostelo RW, Terwee CB. PROMIS Physical Function short forms display item- and scale-level characteristics at least as good as the Roland Morris Disability Questionnaire in patients with chronic low back pain. *Arch Phys Med Rehabil.* 2019.
85. Full KM, Malhotra A, Crist K, Moran K, Kerr J. Assessing psychometric properties of the PROMIS Sleep Disturbance Scale in older adults in independent-living and continuing care retirement communities. *Sleep Health.* 2019;5(1):18-22.
86. Kroenke K, Spitzer RL, Williams JB, Lowe B. An ultra-brief screening scale for anxiety and depression: the PHQ-4. *Psychosomatics.* 2009;50(6):613-621.
87. Choi SW, Schalet B, Cook KF, Cella D. Establishing a common metric for depressive symptoms: linking the BDI-II, CES-D, and PHQ-9 to PROMIS depression. *Psychol Assess.* 2014;26(2):513-527.
88. Schalet BD, Cook KF, Choi SW, Cella D. Establishing a common metric for self-reported anxiety: linking the MASQ, PANAS, and GAD-7 to PROMIS Anxiety. *J Anxiety Disord.* 2014;28(1):88-96.

89. Von Korff M, DeBar LL, Krebs EE, Kerns RD, Deyo RA, Keefe FJ. Graded chronic pain scale revised: mild, bothersome, and high impact chronic pain. *Pain.* 2019.
90. Pickard SL, E; Jiang, R; Pullenayegum, E; Shaw, J; Xie, F; Oppe, F; Boye, K; Chapman, R; Gong, C; Balch, A; Busschbach, J. . United States Valuation of EQ-5D-5L Health States Using an International Protocol. *Value Health.* 2019;22(8):931-941.
91. Sanders GD, Neumann PJ, Basu A, et al. Recommendations for Conduct, Methodological Practices, and Reporting of Cost-effectiveness Analyses: Second Panel on Cost-Effectiveness in Health and Medicine. *JAMA.* 2016;316(10):1093-1103.
92. Stetler CB, Legro MW, Wallace CM, et al. The role of formative evaluation in implementation research and the QUERI experience. *J Gen Intern Med.* 2006;21 Suppl 2:S1-8.
93. Aarons GA, Hurlburt M, Horwitz SM. Advancing a conceptual model of evidence-based practice implementation in public service sectors. *Adm Policy Ment Health.* 2011;38(1):4-23.
94. Glasgow RE, Davidson KW, Dobkin PL, Ockene J, Spring B. Practical behavioral trials to advance evidence-based behavioral medicine. *Ann Behav Med.* 2006;31(1):5-13.
95. Glasgow RE, McKay HG, Boles SM, Vogt TM. Interactive computer technology, behavioral science, and family practice. *J Fam Pract.* 1999;48(6):464-470.
96. Elwyn G, O'Connor A, Stacey D, et al. Developing a quality criteria framework for patient decision aids: online international Delphi consensus process. *BMJ.* 2006;333(7565):417.
97. Flynn RA, L; Scott, SD. Two Approaches to Focus Group Data Collection for Qualitative Health Research: Maximizing Resources and Data Quality. *Int J Qual Methods.* 2018;17:1-9.
98. Tates K, Zwaanswijk M, Otten R, et al. Online focus groups as a tool to collect data in hard-to-include populations: examples from paediatric oncology. *BMC Med Res Methodol.* 2009;9:15.
99. Hogeboom CJ, Sherman KJ, Cherkin DC. Variation in diagnosis and treatment of chronic low back pain by traditional Chinese medicine acupuncturists. *Complemen Ther Med.* 2001;9:154-166.
100. Sherman KJ, Cherkin DC, Wellman RD, et al. A randomized trial comparing yoga, stretching, and a self-care book for chronic low back pain. *Arch Intern Med.* 2011;171(22):2019-2026.
101. Liang KY, Zeger SL. Longitudinal data analysis using generalized linear models. *Biometrika.* 1986;73:13-22.
102. Levin J, Serlin R, Seaman M. A controlled, powerful multiple-comparison strategy for several situaitons. *Psychological Bulletin.* 1994;115:153-159.
103. Hayter AJ. The Maximum Familywise Error Rate of Fisher's Least Significant Difference Test. *J Am Stat Assoc.* 1986;81(396):1000-1004.
104. Wang M, Fitzmaurice GM. A simple imputation method for longitudinal studies with non-ignorable non-responses. *Biom J.* 2006;48(2):302-318.

105. Witt CM, Vertosick EA, Foster NE, et al. The Effect of Patient Characteristics on Acupuncture Treatment Outcomes: An Individual Patient Data Meta-Analysis of 20,827 Chronic Pain Patients in Randomized Controlled Trials. *Clin J Pain.* 2019;35(5):428-434.
106. Drummond MF, Sculpher MJ, Torrance GW, O'Brien B, Stoddart GL. *Methods for the economic evaluation of health care programmes.* Vol Third Ed. Oxford: Oxford University Press; 2005.
107. Drummond MF, Sculpher MJ, Torrance GW, O'Brien BJ, Stoddart GL. *Methods for the Economic Evaluation of Health Care Programmes.* 3rd ed. Oxford: Oxford University Press; 2005.
108. Manca A, Hawkins N, Sculpher MJ. Estimating mean QALYs in trial-based cost-effectiveness analysis: the importance of controlling for baseline utility. *Health Econ.* 2005;14(5):487-496.
109. EuroQol Group. EuroQol: a new facility for the measurement of health-related quality of life. *Health Policy.* 1990;16:199-208.
110. Neumann PJ, Cohen JT, Weinstein MC. Updating cost-effectiveness—the curious resilience of the $50,000-per-QALY threshold. *New England Journal of Medicine.* 2014;371(9):796-797.
111. Efron B, Tibshirani RJ. *An Introduction to the Bootstrap.* Boca Raton, FL: Chapman & Hall/CRC; 1994.
112. Barber JA, Thompson SG. Analysis of cost data in randomized trials: an application of the non-parametric bootstrap. *Statistics in medicine.* 2000;19(23):3219-3236.
113. Nielsen A. Safety in Acupuncture Therapy: Risks and Harms. 2015; https://www.healthyseminars.com/product/safety-acupuncture-therapy-risk-and-harms. Accessed March 3, 2020.
114. Schulz KF, Altman DG, Moher D. CONSORT 2010 Statement: updated guidelines for reporting parallel group randomised trials. *Trials.* 2010;11:32.

15. SUPPLEMENTS/APPENDICES

1. Acupuncture Treatment Protocol with Acupoints

2. Acupuncture Treatment Visit Form

3. Informed Consent Drafts (available on June 30, 2020)
