# Supplementary material for: Acupuncture for Chronic Low Back Pain in Older Adults: A Randomized Clinical Trial
Source: JAMA Netw Open. 2025 Sep 12;8(9):e2531348. doi: 10.1001/jamanetworkopen.2025.31348 (PMC12432643; doi:10.1001/jamanetworkopen.2025.31348)
Supplement: Supplement 2. — Statistical Analysis Plan [file jamanetwopen-e2531348-s002.pdf]

Statistical Analysis Plan

Pragmatic Trial of Acupuncture for Chronic Low Back Pain in Older Adults

(BackinAction)

Principal Investigators:

Lynn L. DeBar, PhD, MPH
Andrea J. Cook, PhD
Senior Scientific Investigators
Kaiser Permanente Washington Health Research Institute

Date: 04/24/2023
Version: 1.3

Table of Contents

Statistical Analysis Plan 1
Pragmatic Trial of Acupuncture for Chronic Low Back Pain in Older Adults 1
1. Protocol Summary 3
1.1 Specific Aims 3
1.2 Study Population 4
1.3 Description of Intervention 4
2. Study Outcomes 4
2.1 Primary Outcome in Effectiveness Analyses 4
2.2 Secondary Outcomes in Effectiveness Analyses 5
2.3 Tertiary Outcomes in Effectiveness Analyses 6
2.4 Economic Evaluation Outcomes 8
3. Additional Variables for Effectiveness Analyses (Moderators and Predictors) 8
4. Sample Size and Treatment Assignment Procedures 10
5. Definition of Study Samples 11
5.1 Participants Inclusion and Exclusion Criteria 11
5.2 Intent-to-Treat (ITT) Sample 12
5.3 Safety Analysis Sample 12
5.4 Dosing and Adherence Samples 12
6. Definition of Treatment Adherence 12
7. Randomization and Blinding 13
8. Multiple Comparisons 13
9. Missing Data 14
10. Outlier Measures 15
11. Demographic and Baseline Characteristics 15
12. Descriptive Analysis 15
13. Effectiveness Analyses on Changes in RMDQ Scores 15
13.1 Primary Time Point: 6-Months Post Randomization 16
13.2 Secondary Time Points: 3- and 12-Months Post Randomization 16
14. Effectiveness Analyses on Secondary Outcomes 16
15. Additional Effectiveness Analyses for Primary Outcome 17
15.1 Moderators 17
15.2 Dosage of Acupuncture Treatment and Adherence 17
16. Exploratory Effectiveness Analyses on Tertiary Outcomes at 3-, 6-, and 12-Months 17
17. Exploratory Effectiveness Analyses Son Monthly Measurements 17

18. Economics Analyses ... 18
19. Safety Monitoring Analyses ... 19
19.1 Adverse Event ... 19
19.2 Serious Adverse Event ... 19
19.3 AE/SAE Reporting Procedure ... 19
19.4 Safety Outcomes ... 20
19.5 Descriptive Analysis ... 20
20. Interim Analyses ... 20
21. References ... 20

1. Protocol Summary

BackInAction (BIA) is a randomized pragmatic trial for investigating effectiveness of acupuncture therapy, including standardized acupuncture (3-months, SA) and enhanced acupuncture (3-months standardized acupuncture and 3-months maintenance acupuncture, EA) on improving back-related disability in older adults 65 years-old and older with chronic low-back pain (cLBP). The investigators use a 3-arm design with two parallel interventions, SA and EA, compared to the usual medical care (UMC).

Participants will be recruited from four healthcare systems (HCSs); Kaiser Permanente Washington - KPWA and Kaiser Permanente Northern California - KPNC, which have Kaiser Permanente integrated health plans; Sutter Health - SH, a largely fee for service organization; and the Institute for Family Health - IFH, a system of federally-qualified health centers (FQHCs). Potential eligible participants will be referred by primary care physicians (PCPs), identified based on electronic health records (EHR) followed by letter or email invitations, or self-referred from direct outreach. The eligibility screening procedure will be conducted via phone or in-person visits. Individuals who screen eligible and provide informed consent will receive a baseline questionnaire via a phone survey.

Once the baseline survey is completed, participants will be randomized to one of the three arms, with stratification by HCS and within that, on age and gender. Participants will be told whether they have been randomized to acupuncture (SA/EA) or UMC. Participants randomized to EA will not be informed that they have been selected to receive additional maintenance treatment sessions until close to the end of the first 3 months of treatment (approximately 10 weeks into the study) so that their treatment is unlikely to be altered by the knowledge of additional visits. Participants will remain in the trial for 12 months and we anticipate the entire duration of enrollment and follow-up will be 29 months. Physical function, pain intensity and pain interference will be measured monthly (short questionnaire) and along with back-related disability and other assessments at 3-, 6-, and 12-months after randomization (long questionnaire).

1.1 Specific Aims

Aim 1:
Primary Objective
We hypothesize that both types of acupuncture will result in improved back-related disability compared to usual medical care at 6 months. We further hypothesize that enhanced acupuncture will be superior to standard acupuncture, albeit not expected to be a clinically important difference.
Secondary Objectives

To examine the effectiveness of acupuncture at 3 and 12 months for improving back-related disability and to evaluate additional outcomes, including the PEG, a 3-item composite measure of pain intensity and pain interference with enjoyment of life and with general activity at 3, 6 and 12 months.

Aim 2: To conduct a cost-effectiveness analysis of enhanced acupuncture and standard acupuncture compared to usual care.

Aim 3: To conduct formative and summative evaluations to understand, describe and explain barriers and facilitators to adoption, implementation, and sustainability of acupuncture treatment for older adults.

1.2 Study Population

The 789 participants in this study will be recruited from the population of more than 90,000 older adults (at least 65 years old) in the four HCSs, who made an ambulatory care visit for uncomplicated cLBP in the 12-month period from December 2018 to November 2019 and who met the EHR-based inclusion and exclusion criteria (see section 6.1) and screen eligible based on their responses to questions administered by an interviewer.

1.3 Description of Intervention

The two interventions in this trial include (1) standardized acupuncture, SA, which will consist of 3 months of acupuncture therapy, with a proposed minimum of 8 treatments and a maximum of 15, and (2) enhanced acupuncture, EA, which will include the standard acupuncture plus an additional 3-month maintenance period, with a proposed minimum of 4 additional acupuncture therapy and a maximum of 6. Treatment visits will typically last 45-60 minutes. All treatments will include only acupuncture needling. Other adjunctive modalities (e.g., moxibustion or other forms of heat, cupping, gua sha, tui na), commonly used in practice, will be proscribed.

The UMC arm will consist of the care that individuals receive according to their insurance benefits plus anything else they pay for out of pocket. We will ask those assigned to UMC to avoid acupuncture for the year they are enrolled in the study. Both active treatment arms (SA, EA) will also have access to UMC as described above. Participants will be enrolled in the study for 12 months.

2. Study Outcomes

2.1 Primary Outcome in Effectiveness Analyses

Primary Outcome	Brief Description of Measure	Outcome Measured By	Time Frame
Change in back-related disability at 6-month post randomization (continuous)	Change in back-related disability is defined as score change in RMDQ, a 24-item questionnaire which asked whether 24 specific activities were limited due to back pain during the past week.	Patient reported outcome (PRO) of scores from RMDQ at baseline and 6-month	Baseline to 6-month

2.2 Secondary Outcomes in Effectiveness Analyses

Secondary Outcome	Brief Description of Measure	Outcome Measured By	Time Frame
Change in back-related disability at 3-, and 12-month (continuous)	See above	PRO of scores from RMDQ at baseline, 3- and 12-month	*Test of Standard Acupuncture:*<br>Baseline to 3 months<br><br>*Test of Maintenance effect:*<br>Baseline to 12 months
Achieving Minimal Clinically Important Difference (MCID) in back-related dysfunction (binary)	MCID as measured by a 30% reduction on the RMDQ (see above)	PRO of scores from RMDQ at baseline, 3-, 6- and 12-month	*Study primary outcome timepoint:* Baseline to 6 months<br><br>*Test of Standard Acupuncture:*<br>Baseline to 3 months<br><br>*Test of Maintenance effect:*<br>Baseline to 12 months
Change in PEG at 3-, 6- and 12-month (continuous)	Change in pain intensity and pain interference with enjoyment of life and general activity will be measured by PEG, a 3-item pain-intensity and pain-related interference composite measure assessing pain intensity, and pain interference with enjoyment of life and general Activity. Each item is measured by a 0 to 10 scale	PRO of scores from the average of the 3-item PEG scale at baseline, 3-, 6- and 12-month	*Study primary outcome timepoint:* Baseline to 6 months<br><br>*Test of Standard Acupuncture:*<br>Baseline to 3 months<br><br>*Test of Maintenance effect:*<br>Baseline to 12 months<br><br>*Exploratory analyses:*<br>Monthly measures<br>Baseline to 12 months
Achieving MCID in PEG (binary)	MCID as measured by a 30% reduction on PEG	At least 30% improvement from baseline on the PEG PRO at 3-, 6- and 12-month	*Study primary outcome timepoint:* Baseline to 6 months<br><br>*Test of Standard Acupuncture:*<br>Baseline to 3 months<br><br>*Test of Maintenance effect:*<br>Baseline to 12 months

Secondary Outcome	Brief Description of Measure	Outcome Measured By	Time Frame
Change in physical functioning at 3-, 6- and 12-month (continuous)	PROMIS Physical functioning short form 6b/PROMIS-29 subscale (6 items)	PRO of scores from PROMIS® physical function subscale at baseline, 3-, 6- and 12-month	*Study primary outcome timepoint:* Baseline to 6 months<br><br>*Test of Standard Acupuncture:*<br>Baseline to 3 months<br><br>*Test of Maintenance effect:*<br>Baseline to 12 months<br><br>*Exploratory analyses:*<br>Monthly measures<br>Baseline to 12 months
Patient global impression of change (PGIC)	Guy/Farrar Patient Global Impression of Change in Overall Pain (1 item)	PRO of scores at baseline, 3-, 6- and 12-month	*Study primary outcome timepoint:* 6 months<br><br>*Test of Standard Acupuncture:*<br>3 months<br><br>*Test of Maintenance effect:*<br>12 months

2.3 Tertiary Outcomes in Effectiveness Analyses

Tertiary Outcome	Brief Description of Measure	Outcome Measured By	Time Frame
Change in sleep duration at 3-, 6- and 12-month and in sleep during at 6-months(continuous)	Change in sleep quality will be measured by the 6-item subscale for sleep quality in PROMIS® and a 1-item sleep duration scale for sleep duration	PRO of scores from PROMIS® sleep quality subscale at baseline, 6-month or one question on sleep duration at baseline, 3-, 6- and 12-months.	*Study primary outcome timepoint:*<br>Baseline to 6 months (sleep quality and duration)<br><br>*Test of Standard Acupuncture:*<br>Baseline to 3 months (sleep duration only)<br><br>*Test of Maintenance effect:*<br>Baseline to 12 months (sleep duration only)

Tertiary Outcome	Brief Description of Measure	Outcome Measured By	Time Frame
Change in symptoms suggesting clinically relevant anxiety at 3-, 6- and 12-month (continuous)	Change in PRO scores about the frequency of being anxious or having uncontrollable worry over the past two weeks from the GAD-2, which is part of the PHQ-4.	PRO of scores from anxiety subscale of the PHQ-4 at baseline, 3-, 6- and 12-month	*Study primary outcome timepoint:* Baseline to 6 months<br><br>*Test of Standard Acupuncture:* Baseline to 3 months<br><br>*Test of Maintenance effect:* Baseline to 12 months
Change in symptoms suggesting depression at 3-, 6- and 12-month (continuous)	Change in PRO scores about the frequency of depressed mood and anhedonia over the past two weeks from the PHQ-2.	PRO of scores from depression subscale of the PHQ-4 at baseline, 3-, 6- and 12-month	*Study primary outcome timepoint:* Baseline to 6 months<br><br>*Test of Standard Acupuncture:* Baseline to 3 months<br><br>*Test of Maintenance effect:* Baseline to 12 months
Change in fatigue at 3-, 6- and 12-month (continuous)	Change in fatigue will be measured by the 4-item subscale for fatigue in PROMIS®	PRO of scores from PROMIS® fatigue subscale at baseline, 3-, 6- and 12-month	*Study primary outcome timepoint:* Baseline to 6 months<br><br>*Test of Standard Acupuncture:* Baseline to 3 months<br><br>*Test of Maintenance effect:* Baseline to 12 months
Change in ability to engage in social roles at 3-, 6- and 12-month (continuous)	Change in ability to engage in social roles will be measured by the 4-item subscale for ability to participate in social roles in PROMIS®	PRO of scores from PROMIS® ability to participate in social roles at baseline, 3-, 6- and 12-month	*Study primary outcome timepoint:* Baseline to 6 months<br><br>*Test of Standard Acupuncture:* Baseline to 3 months<br><br>*Test of Maintenance effect:* Baseline to 12 months

2.4 Economic Evaluation Outcomes

Cost Outcomes	Brief Description of Measures	Measured By	Time Frame
Cost and incremental cost-effectiveness	Health care utilization and intervention costs will be assessed.<br><br>Our primary measure of effectiveness (utility) at 12 months will be quality-adjusted life years (QALYs) gained<br><br>We will also estimate the incremental cost per additional patient with a MCID (30% from baseline) reduction in the RMDQ at 12 months.	Healthcare utilization costs: EHR data costed using standard costing algorithms [[1,2] and Medicare fee schedules<br><br>Intervention costs: Acupuncture sessions will be tracked in study records and priced using typical community rates<br><br>Utilities will be estimated using the EQ-5D-5L[3] and be used to calculate QALYs gained<br><br>PRO of scores from RMDQ at baseline and 12 months	Patient health care utilization costs from baseline to 12 months net of their utilization from the previousadj 12 months<br><br>EQ-5D-5L: Patient self-report at baseline, 3-, 6- and 12-months

3. Additional Variables for Effectiveness Analyses (Moderators and Predictors)

The following predictors/covariates and/or **moderators** will be assessed related to the **primary outcome.** Note: variables pre-specified for baseline adjustment in all models include: baseline outcome value, age, sex, race, and healthcare system

Moderators & Predictors	*Definition*	Role	Data Source
Sex	Male vs. Female/Other Sex at birth as reported by subject; assessed using HEAL CDE Demographic question	Moderator/Predictor	Patient self-report at baseline
Age	65-74, 75-84, 85+	Moderator/Predictor	EHR
Race/Ethnicity	White, non-white	Moderator/Predictor	Patient self-report at baseline
Expectations of acupuncture	EXPECT 1-item question – categorical based on the distribution of responses	Moderator/Predictor	Patient self-report at baseline
RMDQ	<18, 18+	Moderator/Predictor	Patient self-report at baseline
Pain catastrophizing	6-item subscale required by HEAL CDE	Predictor/covariate	Patient self-report at baseline

Moderators & Predictors	*Definition*	Role	Data Source
Fear avoidance	NIH Task Force single item	Predictor/covariate	Patient self-report at baseline
Frailty	Frailty Index[[4]	Predictor/covariate	Patient self-report at baseline and EHR data
Medical morbidity	Carey Ambulatory-based Morbidity Score [5]	Predictor/covariate	EHR data; based on last 12 months
Multiple non-malignant musculoskeletal pain conditions	1 pain cluster vs. >1<br><br>> 1 pain-related ICD-10 diagnosis corresponding to more than one (>1) of the non-malignant musculoskeletal chronic pain condition clusters developed for the National Pain Strategy chronic pain condition clusters[6]<br>Back pain<br>Neck pain<br>Limb/extremity pain, joint pain and arthritic disorders<br>Fibromyalgia<br>Headache<br>Orofacial, ear, and temporomandibular disorder pain<br>Musculoskeletal chest pain<br>General pain subcategory of the Other painful conditions cluster	Predictor/covariate	EHR data; diagnoses in participant's EHR extracted at baseline for prior 360 days
Mental health mood disorders	ICD-10 diagnosis for depression and/or anxiety diagnosis	Predictor/Covariate	EHR data; diagnoses in subject's EHR extracted at baseline for prior 360 days

Moderators & Predictors	*Definition*	Role	Data Source
COVID19 pandemic impact	Two questions modified from assessments designed by Pain Collaboratory. These focus on impacts of ability to get health care and on overall health	Time-varying covariate	Patient self-report
Sample Recruitment Time	Indicators of changes in sampling scheme to over-recruit certain racial and ethnic groups to improve representation and power for subgroup analyses	Covariate	Derived from randomization and changes in sampling distribution

4. Sample Size and Treatment Assignment Procedures

We determined our sample size requirements for our primary outcome the RMDQ at 6-months that focuses on detecting differences of each acupuncture group compared to UMC. Recruited older adults with cLBP will be randomized, in equal proportions, to one of the three groups (UMC, SA, and EA). The power calculation below was conducted under assumptions of a 20% loss-to-follow-up rate and a SD of 6 points in RMDQ score in each arm (consistent or larger then results from previous trials)[7] [8] [9], and that pairwise comparisons will only be conducted if the omnibus F-test is statistically significant at a 0.05 level to control for multiple comparisons (Fisher's Least Significance Difference multi-comparison approach). Given a sample of 789 total participants (263 per arm) among whom 630 (210 per arm) complete their 6-month follow-up data collection, we will have at least 90% power to detect a MCID of 2 points on the RMDQ score between each acupuncture group and UMC (pair-wise comparison power). Nevertheless, in the scenario where SA at 6-months attenuates to be equivalent to UMC and EA has a 2-point MCID improvement relative to both SA and UMC, we will have 91% power to detect a difference between SA and EA as well as between UMC and EA (each pair-wise comparison).

The above power calculation was conducted with RMDQ score as the outcome. When the outcome of interested is the change on RMDQ score from baseline to 6-month, we expect a smaller SD than 6 points in each arm (based on previous trial data conducted at KPWHRI only the SD RMDQ at 6 months was 5.42 and change in RMDQ was 5.11[8]). Thus, we are sufficiently powered (≥90%) to detect MCID differences between all pairwise group comparisons. Power was calculated via simulation using R software version 3.6 accounting for multiple comparisons of the three study arms using Fisher's Least significant different approach. Note to be conservative we assumed a larger SD then we expect since we may have loss to power due to clustering of participants within acupuncture provider (acupuncturists may see 10-15 participants) and we may have more variability in baseline outcome since this trial is pragmatic across four health care systems. Inflating the SD by more than 15% (6/5.11=1.17) should take into account both potential loss to power issues. Furthermore, although acupuncturists may see up to 10-15 participants at our smallest site (IFH) this accounts for 16% of our expected recruited sample (123 of 789 with only two-thirds of those randomized to an acupuncture arm where clustering is of issue). At other sites, the combination of HCS embedded and community acupuncturists are expected to result in much small caseloads (3-8 participants on average). In keeping with a pragmatic trial approach and to lower barriers to participant receipt of acupuncturists, we are motivated to include a larger number of acupuncturists serving participants in the study

For any two group comparisons, given our sample size of 210 per group and a SD of 6, the 95% CI width around the difference in means between groups is +/- 1.15 pts. Further, for secondary analysis for the binary outcome 30% improvement in RMDQ from baseline we have >90% power to detect an MCID assuming the probability of improvement in UMC was between 33% [10] and 44% [7] and the MCID was a 15% improvement above UMC for each of the acupuncture groups. We will use an intent-to-treat approach in which participants will remain as randomized regardless if they withdrawal from treatment or cross-over to other treatment arm (e.g. UMC participant seeks acupuncture outside of study).

5. Definition of Study Samples

5.1 Participants Inclusion and Exclusion Criteria

We will require all participants to meet all the following inclusion criteria in order to participate in the trial.

Inclusion Criteria	Rationale and Source
Is at least 65 years of age	Age range of the Medicare older adult population (EHR)
Is a current member or patient of the healthcare system	A method for identifying participants who have current and consistent contact with the healthcare system (EHR and PRO)
Visited a health care provider for low back pain within the past 12 months	A method for identifying potential participants who may have cLBP (EHR)
Received primary care at one of the participating health care systems.	Location of our study sites (EHR)
Has back pain that is uncomplicated with or without radicular pain.	This is the type of back pain we are studying. (EHR)
Back pain $\geq$ 3 months	Meets our definition of chronic back pain (PRO)
General activity question from PEG $\geq$3	Meets minimum definition of back dysfunction (PRO)
Primary care provider provides permission to contact patient	Ensures that there is no medical or related reason not to include patient (via email)
Willing and able (Callahan screener $\geq$ 3) to provide consent	Ethical requirement (PRO)

Persons who meet any of the exclusion criteria at baseline will be excluded from study participation.

Exclusion Criteria	Rationale
Specific types of back pain (metastatic cancer or bone cancer or secondary cancers, vertebral fractures, spinal infection, active inflammatory disease)	Other treatments are more appropriate than acupuncture for these specific causes (or likely causes) of LBP (EHR)
Low back surgery within past 3 months	May still be healing from surgery (PRO)

Receiving workers compensation or involved in litigation related to cLBP	Additional treatments may be required as there are disincentives to improve (PRO)
Acupuncture within the last 6 months	Ensures that they have not received acupuncture for this episode of care (PRO)
Does not speak or write English or Spanish	Cannot complete outcomes questionnaires or treatments (PRO)
Major psychosis, dementia	Unable to give adequately informed consent (EHR)
Current cancer treatment	Need for primary focus on cancer treatment (EHR)
Red flags of serious underlying illness (a fever most days in the last month, recent unexplained weight loss of 10 lbs or more)	Need to look for serious underlying illness to not delay any needed treatment for those. (PRO)
Living in a nursing home, on Hospice, or palliative care	Requires a different study design and logistics (EHR; confirm via PRO)
Non-speaking deafness	Cannot communicate with acupuncturists and study staff (EHR)
Non-reliable transportation	Cannot attend acupuncture treatments (PRO)

5.2 Intent-to-Treat (ITT) Sample

All study participants randomized meeting eligibility criteria prior to randomization will be included in the ITT sample and will be analyzed according to their assignment group at the time of randomization. Note we will exclude participants if they were randomized but after further review based on data collected prior to randomization the participants did not meet all eligibility criteria. These participants will be listed as protocol violations.

5.3 Safety Analysis Sample

The safety analysis will include all randomized participants in the sample in the 12-months period of follow-up.

5.4 Dosage and Adherence Samples

The dosage exploratory analysis will include everyone with a 3-month outcome for the SA, EA, and UMC groups. We will use the number of acupuncture treatments received regardless of which group a person was randomized to as their dose. For adherence analyses we will include 1) participants in the acupuncture groups who, in the 3 months of standard acupuncture, received at least 8 treatments and the last visit indicated that they had completed treatment or received at least 12 treatments and 2) amongst those with last visit indicated that they had completed treatment (see Protocol section 5.4. for our proposed operationalization criteria for "completed treatment").

6. Definition of Treatment Adherence

Treatment adherence in SA group is defined as (1) receiving a minimum of 8 acupuncture treatment in the 3-month of standard acupuncture period with the last clinical visit indicating a completion of treatment or (2) receiving at least 12 acupuncture treatments in the standard acupuncture period. For EA group, treatment

adherence is defined as, in addition to the adherence defined in SA group, (1) receiving at least 4 maintenance acupuncture or (2) a note of completion remarked by acupuncturists. Participants who are randomized to the EA group will be asked to attend the maintenance visits.

7. Randomization and Blinding

After completion of baseline questionnaire, participants will be randomized via a computer-generated randomization scheme in R developed by a study biostatistician in a 1:1:1 ratio to study condition (SA, EA, or UMC) stratified by HCS (KPWA; KPNC; SH; IFH), age group (65-74; 75-84; 85+) and sex. Stratification is only being used to maintain balance of treatment assignment with stratum and we do not have sample size requirements within a given stratum. We will employ random blocks of size 3 and 6 to ensure balance of groups over time as well as blinding of study team to next randomization assignment. The biostatistician will keep the randomization file in a secure folder only accessible to the biostatisticians and programmer. The study programmer will be given the randomization scheme within specified strata and the program will only allow participants to be randomized once they consent and complete the baseline questionnaire. The study interviewer will press a button and the appropriate group assignment (acupuncture, UMC) will appear. This method ensures that treatment allocation cannot be changed after randomization.

After the generic randomization schemes (Arm 1, Arm 2, Arm 3) have been generated by the biostatistician, the programmer will assign a code to each of the be the only one to have access to the randomization schemes that are embedded in the program. The coding will be held in a secure folder. Other study personnel including principal investigators will only receive unmasked summary information after the completion of the intervention and the database is locked. During the Independent Monitoring Committee (IMC) reporting treatment assignment will be masked unless requested by the IMC. The programmer will run the reports for the IMC meetings.

Blinding

This is an unmasked trial for participants, although the participants assigned to SA and EA will only know they are randomized to acupuncture groups at the time of randomization. Participants assigned to EA group will not be informed about additional maintenance treatment sessions until close to the end of the first 3 months of treatment (approximately 10 weeks into the study) so that their treatment is unlikely to be altered by the knowledge of additional visits. Acupuncturists will not know whether their patients are in the SA or EA group until 10 weeks into the standard treatment period. For qualitative data collection, interviewers are expected to be unmasked to participants treatment condition. No members of the Core Executive Team (Multiple PI's, site PI's and the Statistical Methods Committee, which includes the study biostatisticians) will have access to the outcomes data during the course of the trial. Conceivably, a site PI or one of the Multiple PI's may become aware of an individual's treatment group if they have an Adverse Event that requires the investigator intervene. However, one biostatistician, Dr. Piccorelli, will be partially unblinded only to the proportion of missing data by masked intervention group and other baseline characteristics by masked intervention group which are included in the closed report of the DSMB. Dr. Piccorelli will not have direct access to the actual outcome data or any information about the distribution of the non-missing outcome data and is only lending statistical expertise to ongoing trial monitoring and reporting for purpose of correspondence with the DSMB and NIH. All other members of the CET will become unblinded after the data base is locked. All primary outcome analyses for the main results paper of the trial will be conducted by a fully blinded biostatistician, Mr. Wellman.

8. Multiple Comparisons

We have one primary outcome – the change on RMDQ scores for low-back pain related disability at 6 months since baseline. To control for multiple comparisons due to 3 intervention groups we will apply Fisher's Least Significant Difference approach in which first an omnibus Wald-test for any statistically significant difference

between the three groups is evaluated at the 0.05 alpha level. If the omnibus test is statistically significant, then the pair-wise differences are then evaluated, each using two-sided alpha=0.05. If the omnibus test is not statistically significant, we will conclude that there is no benefit of acupuncture for that time point. In addition, secondary analyses will use a similar approach as that described for the primary outcome to control for the three group comparisons.

9. Missing Data

The investigators and data managers will monitor data collection process on a weekly basis and ensure a minimal amount of loss to follow-up in the study. All analyses will be conducted following an intent-to-treat approach, including all individuals randomized regardless of their engagement with, or exposure, to the intervention. If missing data for RMDQ is minimal, our primary analysis will be a complete case analysis adjusting for baseline RMDQ score, age, sex, race and HCS. If missing data for RMDQ at 6 months is above 15% in any arm, or differential by 10% between any two arms, then for our primary analysis we will employ missing not at random imputation techniques to address missing data issues.[11] Though the metric used to make decisions about the analysis method focuses on missing data at the primary time point (6 months), if criteria are met missing data will be imputed 3, 6 and 12-month time points. However, our focus will be on minimizing missing data, and in our collective substantial experience conducting similar trials, we have consistently had retention in line with this. Should it be necessary, the imputation method we propose, derived for use with GEE and sensitive to potential non-ignorable missingness, includes all follow up time points in the same model and uses a pattern mixture approach that relaxes the missing at random given baseline covariates assumption. This accomplished via the implementation of an imputation model which includes indicators classifying the mutually exclusive missing data patterns observed across all time points in the data. We will describe all of the missing data patterns present in the observed data to identify a scientifically reasonable and estimable set of patterns to use as indicators in imputation models for each outcome. Specifically, following the recommendations of Wang and Fitzmaurice[11], we plan to use the most flexible (parameterizing the largest number of missing patterns) that are estimable with the data. We will construct the imputation models such that the effects of missing data on the outcome are allowed to vary by treatment arm and follow-up time and will include main effects for all covariates used as stratification factors in the study randomization and/or thought to be potential confounders of the relationship between outcome and treatment arm. Further, we will include additional baseline variables that are predictive of missing the 6 months outcome. Specifically, baseline covariates that are significantly (at 0.10 significance level) associated with missingness at 6 months using flexible logistic regression models will be included as main effects in imputation models.

Following imputation of the data using the models described here, final effect estimates will be obtained in the full imputed data using GEE with the functional model form described in section 13 below, where the vector of covariates, $\boldsymbol{Z}$, includes stratification variables, potential confounders and variables identified as being significantly associated with missingness. Standard error estimates will be calculated using the sandwich-style estimator derived by Wang and Fitzmaurice[11], which takes into account variability due to imputation and is shown to provide consistent estimates of the true standard errors. It is an extension of the standard GEE model which is incorporating the variability due to multiple time points and acupuncturists as proposed in our primary analysis model (see Section 13), but adds imputation to relax the missing at random given baseline covariates assumption of standard GEE. Because the sites participating in this study represent multiple disparate health systems each with its own unique member populations, culture, guidelines, and practices, we will conduct a sensitivity analysis allowing the effect of potentially non-ignorable missing data patterns to vary by site and treatment group at each follow-up time point instead of just including site indicators as main effects in the imputation model. Further sensitivity analyses related to the choice of missing data patterns will be considered as necessary.

10. Outlier Measures

The occurrence of outlier measures in this study will be unlikely since most measurements are obtained from questionnaires with a limited range of values; range and logic checks will be built into the outcomes database. Thus, we will not correct for any outlier in the effectiveness analyses.

11. Demographic and Baseline Characteristics

Demographic variables, including sex, age, race and ethnicity, will be extracted from EHR, and summarized for each intervention arm. We define baseline characteristics as information collected during baseline interview (pre-randomization), including but not limited to employment status, education, marital status, income, tobacco use, alcohol use, duration of pain condition or any variables potentially associated with primary and secondary outcomes, moderating the impact of acupuncture treatments, and predictive for loss to follow-up.

12. Descriptive Analysis

Descriptive statistics for all follow-up data will be provided for each intervention. For continuous variables we will include the mean, median, SD, maximum and minimum. Frequencies, percentage, and tabulations will be provided for categorical variables. Summary statistics will be performed on patient demographics and baseline characteristics.

13. Effectiveness Analyses on Changes in RMDQ Scores

We will evaluate the effectiveness of acupuncture and acupuncture plus maintenance relative to Usual Medical Care (UMC) at 3-, 6- (primary time-point), and 12-months after randomization. We will conduct a longitudinal analysis including the continuous outcome, change in Roland Morris Disability Questionnaire (RMDQ) from baseline (primary outcome) measured at all follow-up times, in one model estimated using generalized estimating equations (GEE). [12] We will use a working independence correlation matrix and will calculate standard errors using the robust sandwich estimator to account for within-person and within-in provider (some participants may see the same provider) correlation.[12] All models will adjust for baseline RMDQ score, age, sex, race and HCS. Further, if missing RMDQ at 6 months (primary time point) is more than 15% in any study arm, or differential by more than 10% between any two arms, we will use imputation approaches as the primary analysis to account for missing data and further adjust for any baseline variables that are predictive of loss to follow-up determined as described in Section 9. All analyses will be conducted following an intent-to-treat approach, including all individuals randomized regardless of their engagement with, or exposure, to the intervention.

We will include interactions between intervention groups and time (3-, 6-, and 12-months) to estimate time-specific intervention effects. Time will be included as a categorical variable. To gain power, since acupuncture and acupuncture plus maintenance at 3-months are the same intervention (maintenance period occurs between 3 and 6 months post randomization and neither participants in the EA condition nor the acupuncturists treating them will be aware they will receive maintenance treatments until close to the 3-month follow-up), we will combine acupuncture groups at 3-month follow-up. We chose GEE as our analytic method because our primary outcome, RMDQ, is not expected to be normally distributed. From our experience working with RMDQ data, adjusting for baseline RMDQ value results in more normally distributed residuals. However, we didn’t want to make that assumption a priori and therefore chose GEE.

Our specific model for change in RMDQ is the following:

(1)
$$E(Y_{im}|T_i, \boldsymbol{Z}_i) = \alpha + \beta_{Acu} I(T_i = SA \text{ or } EA) + \boldsymbol{\beta}_z \boldsymbol{Z}_i + \beta_6 I(m = 6) + \beta_{12} I(m = 12)$$
$$+ \gamma_{SA,6} I(\mathrm{T_i} = SA) I(m = 6) + \gamma_{SA,12} I(\mathrm{T_i} = SA) I(m = 12)$$
$$+ \gamma_{EA,6} I(\mathrm{T_i} = EA) I(m = 6) + \gamma_{EA,12} I(\mathrm{T_i} = EA) I(m = 12)$$

where Y_{ij} is the change in RMDQ score from baseline for individual *i* at time point m, $\mathrm{T_i}$ is the intervention group, m is the number of months post randomization, and $\mathbf{Z}_\mathrm{i}$ is the vector of baseline covariates as stated in the beginning of this section, respectively. The parameters α, β's and γ's are the intercept, main effects, and the interaction effects of the corresponding category indicators in the subscripts.

13.1 Primary Time Point: 6-Months Post Randomization

For the 6-month time point we will conduct a sequential series of analyses after fitting the model in equation (1). The difference in change in RMDQ at 6-months between the two acupuncture groups, SA and EA, will be assessed first by testing $\gamma_{SA,6} = \gamma_{EA,6}$. If a statistically significant (α=0.05) and meaningful difference (>1 pt difference) is found between SA and EA (Scenario 1), comparisons of each acupuncture group to UMC will be carried out individually. Scenario 1 assessments will determine (1) if acupuncture treatment with additional maintenance period (EA) is better than the standard acupuncture treatment (SA) at 6-months and (2) if either or both acupuncture groups are better than UMC. In an alternative scenario where SA and EA do not differ at 6-months (Scenario 2), the two acupuncture groups will be combined for the time point of 6-months and run a second regression model (Model 2) including only UMC and the combined acupuncture group. If this regression model shows that acupuncture is better than UMC, we will conclude that acupuncture improved RMDQ at 6-months, but maintenance was not shown to be effective.

13.2 Secondary Time Points: 3- and 12-Months Post Randomization

The acupuncture's impact on the change of RMDQ scores at 3-months will be assessed by testing if $\beta_{Acu} = 0$ in equation (1) since acupuncture and acupuncture plus maintenance at 3-months are the same intervention (maintenance period occurs between 3 and 6 months post randomization and neither participants in the EA condition nor the acupuncturists treating them will be aware they will receive maintenance treatments until close to the 3-month follow-up). We will follow the same general framework for 12-months as we have specified for 6-months in Section 13.1. Note that the analysis focused on the 12-month follow-up timepoint provides an important test of whether EA makes an appreciable difference over SA in sustaining or improving the effect of acupuncture on pain-related functioning that endures after the end of treatment. Finally, note that we include all times points in a single GEE model within this general modeling framework to handle correlation due to multiple outcomes on a given person. We will report both the fully saturated model 1 results and an additional final model if scenario 2 is correct for either 6 or 12 months. For example, if scenario 2 is concluded for both time points 6 and 12 months the final model is:

Scenario 2 is correct for 6 and 12 months:

(2) $$E(Y_{im}|T_i, \boldsymbol{Z}_i) = \alpha + \beta_{Acu} I(T_i = SA \text{ or } EA) + \beta_z \boldsymbol{Z}_i + \beta_6 I(m = 6) + \beta_{12} I(m = 12) + \gamma_{Acu,6} I(\mathrm{T_i} = SA \text{ } or \text{ } EA) I(m = 6) + \gamma_{Acu,12} I(\mathrm{T_i} = SA \text{ } or \text{ } EA) I(m = 12).$$

14. Effectiveness Analyses on Secondary Outcomes

Secondary outcomes including the PEG, patient global impression of change for pain, and physical function will be evaluated at 3-, 6-, and 12-months post randomization by comparing the measurements at the three time points to the baseline. We will follow similar framework as described above for RMDQ scores to analyze the effectiveness of acupuncture treatments, SA and EA, on the secondary outcomes. In addition, the occurrence of an ≥30% improvement in RMDQ and PEG measures at 3-, 6-, and 12-months compared to the baseline will be evaluated under the same framework. A link function will be chosen appropriately for each outcome.

15. Additional Effectiveness Analyses for Primary Outcome

15.1 Moderators

The five potential effect moderators including sex (male and female), age group (65-74, 75-84, 85+), race/ethnicity (non-Hispanic white and other), patient expectation (measured with PRO, see Section 4), and RMDQ at baseline (<18, 18+) will be evaluated by investigating their impact on acupuncture's effectiveness on RMDQ score change at 6-months (primary time point) compared to the baseline outcome. We will follow the proposed framework in Section 13.1 with additional interaction terms between individual moderators, intervention, and indicator of 6=months in model (1) and (2). The moderators will be assessed separately. For the race and ethnicity moderator we will further adjust for a spline of calendar time to account for potential calendar time confounding due to over-sampling certain racial and ethnic subgroups to increase numbers to improve representation. For the knots in the spline we will use the times when we changed the sampling proportions of racial groups across the study. Other clinically meaningful moderators, for example cognitive impairment, frail elderly, co-morbid pain conditions, co-morbid depression, will be considered given there is greater than 10% of participants in the sample for a given subgroup of interest. All moderator analyses that are conducted will be reported in a single manuscript. Interpretation of findings therefore will be in context of number of tests being conducted and are only exploratory findings.

15.2 Dosage of Acupuncture Treatment and Adherence

We will flexibly model the change of the primary outcome at 3 months by number of treatments to see if there is a threshold effect of treatment dose. Further we will run adherence analyses comparing amongst those who adhered in the 3-months of SA (at least 8 treatments and last visit indicated by acupuncturist that they completed treatment or at least 12 treatments) compared to UMC and amongst those adhered to those who did not adhere within the SA groups. A second set of adherence analyses will be carried out with an alternative definition of adherence as only those who the acupuncturist indicated as completing treatment. Since these analyses are not intent-to-treat, we will include further potential confounders which are potential predictors for being non-adherent or receiving differential number of treatments.

16. Exploratory Effectiveness Analyses on Tertiary Outcomes at 3-, 6-, and 12-Months

We will assess the tertiary outcomes including PROMIS measures of ability to engage in social roles, anxiety, depression, fatigue, and sleep disturbance/duration in exploratory analyses. These measures are related to other common complaints which acupuncture treatment may have impact on, so improvements on these metrics after acupuncture treatment will be expected. However, since there are less data linking them to acupuncture, especially in a chronic pain population, we will conduct only exploratory analyses on these outcomes. We will fit models for these outcomes separately similar to the model for secondary outcomes at 3-, 6-, and 12-months as described in Section 15 and observe the estimated effects of acupuncture treatments.

17. Exploratory Effectiveness Analyses on Monthly Measurements

Monthly measurements of the secondary outcomes, including pain intensity, pain interference, and physical function, will be assessed by addressing (1) the trajectory of how long it takes until patients improve (i.e, demonstrate a 30% improvement) and (2) what proportion of people receiving acupuncture treatments improve at three months if they don't improve (30% improvement) after one or two months of acupuncture treatment. Address these questions will help understand how much acupuncture is needed to improve and at what time should acupuncture treatment stop if improvement is not observed by then, given each individual's outcome trajectory. We will examine individuals' longitudinal trajectory on the monthly measurements in the exploratory analyses. In addition, we will conduct exploratory per protocol analyses assessing dose response to evaluate

the impact of numbers of received acupuncture treatments on improving the secondary outcomes. The UMC group will be included with their numbers of received treatments assigned as 0 in the analyses.

18. Economics Analyses

We will conduct full economic evaluations (cost-utility and cost-effectiveness analyses) of SA and EA compared to UMC according to Medicare (payer) and health care sector perspectives and following economic evaluation best practices [13] [14,15].These full analyses will be conducted for the Kaiser Permanente and Sutter Health clinical sites where the capture of all health care utilization is available through administrative data from the Health Care Systems Research Network Virtual Data Warehouse (HCSRN VDW). Unfortunately, IFH is not a member of this VDW and only provides primary care. However, as we perform these analyses, we will explore how to capture some comparable usage and cost data for IFH.

The cost-utility analyses will use gains quality-adjusted life-years (QALYs) measured using the EQ-5D-5L [3] across the year to calculate QALYs following an area under the curve approach correcting for baseline [16]. The cost-effectiveness analyses will use the proportion of participants in each group who experienced at 12 months MCID (30%) reduction in the RMDQ from baseline.

<u>Costs to be collected</u>. Medical care utilization and intervention costs will be considered. Medical care utilization includes pharmacy, outpatient visits (including specialty care), inpatient stays, and referrals and will be captured from the VDW. For the Medicare perspective this health care utilization, whether provided within Medicare Advantage programs, Medicare fee for service programs or other health plans for those still on their employers' plans, will be valued using standard costing algorithms[1,2] and Medicare fee schedules. For the health care sector perspective, we will add in estimates of patients' out-of-pocket (e.g., copay) amounts by cost category. The cost of the intervention (acupuncture) will be captured from study records (number of acupuncture treatment sessions received) and valued using typical community rates. For the Medicare perspective we will analyze different assumptions for the amount reimbursed by Medicare.

<u>Cost-effectiveness calculations</u>**.** We will aggregate and calculate the incremental average cost per participant in each treatment arm (SA and EA) compared to UMC (C_{SA} - C_{UMC} and C_{EA} - C_{UMC}) and compare those incremental costs to incremental benefits between groups in terms of QALYs gained and additional patients who saw at least a MCID reduction in RMDQ. If costs of either of the acupuncture arms compared to UMC are reduced and effectiveness increased it will be said to be cost saving and to dominate UMC in terms of cost effectiveness [15]. If incremental costs and effectiveness are both increased then an incremental cost-effectiveness ratio will be calculated and compared to society's willingness-to-pay for an additional QALY (often $50,000 to $100,000 per QALY is used) [17] to see if it can be considered cost-effective.

Because acupuncture may influence other common CLBP comorbidities (e.g., depression and sleep) we will capture both total healthcare utilization for our base case and back-pain-related-only utilization to be included in a sensitivity analysis. We will calculate overall cost-effectiveness as well as the cost-effectiveness by site so that we can examine differences in healthcare utilization and its changes across sites. All cost effectiveness analyses will follow intention to treat.
A bootstrap methodology will be used to estimate confidence intervals, [18] [19] as well as to produce cost-effectiveness planes and cost-effectiveness acceptability curves to show variation around our results [15]. One-way sensitivity analyses will be performed to determine the robustness of our estimates with different assumptions such as the reimbursement rate for acupuncture and the inclusion of only back-related costs [15].

19. Safety Monitoring Analyses

The Data Safety Monitoring plan (DSMP) contains further detail, but we briefly summarize the general framework here. Safety monitoring analyses will be prepared for the external Independent Monitoring Committee (IMC) align with the IMC meeting schedule (see DSMP for exact schedule).

19.1 Adverse Event

A non-serious AE will be defined as an unfavorable and unintended diagnosis, symptom, syndrome, or disease that occurs or worsens during the acupuncture intervention period and is plausibly related to acupuncture, including bleeding and needling pain. AEs will be collected in multiple ways: (1) during 3-, 6- and 12-month follow up assessments by querying whether the participant believed that anything about their acupuncture treatments caused significant discomfort or pain and, if so, how long that lasted; (2) via electronic acupuncturist treatment reports; and (3) from participants who may phone the study team at any time to report AEs. Because acupuncture has relatively short-term physiological effects, we will not report AEs that first manifest more than 30-days after a participant's final acupuncture treatment.

We will classify each non-Serious AE using the following definitions: Mild (transient or minimal symptoms; no changes in activity level; no therapy or only symptomatic therapy; Moderate (symptomatic with moderate changes in activity level; no decrease in social activities; specific therapy required); Severe (incapacitating; bed rest; substantial decrease in social activities; loss of work). These definitions are consistent with the International Council for Harmonisation (ICH) standards in characterizing AEs. In the unlikely event that an adverse effect occurs that requires medical care, treatment will be provided as covered by participants' existing health care coverage. We will also assess the likely relatedness of the adverse event and acupuncture given the nature of the event, the timing related to treatment and any important contextual factors.

19.2 Serious Adverse Event

For the proposed study, we are operationally defining a serious adverse event (SAE) as a death or hospitalization, prolongation of a hospitalization or other serious or life-threatening event during a patient's active participation in the trial and study acupuncture treatments. We will review/query active study participants' EHR data every month to identify deaths and hospitalizations among enrolled participants. In the case of a death, a chart review will be conducted by an independent physician at the clinical site to assess whether the death was related to the study intervention (definitely, probably, possibly, or unrelated to the study intervention). For hospitalization, a study clinician at each HCS will review the list of diagnoses for possible relatedness to the acupuncture intervention. For any diagnosis at least possibly related to acupuncture, a more in-depth examination of the medical chart will be conducted.

19.3 AE/SAE Reporting Procedure

A report of AEs will be reviewed by the PIs and Co-Investigators every month and by the IMC every six months. Reports of AE's will be reviewed from the 3 and 6-month questionnaires at regular study meetings. They will be signed off by the Site PI and a study physician. If acupuncturists or patients report an AE, study staff will complete an AE form and follow-up with the patient as appropriate. Serious adverse events that are at least possibly related to acupuncture will be dealt with as soon as the study staff are aware of them. Non-serious AE's will be reported to the IRB yearly and to the IMC at the next meeting. Incidents or events that meet the OHRP criteria for unanticipated problems will be reported to the IRB and the BIA IMC. We will report Unanticipated Problems to our NCCIH Program Officer and OCRA and the BIA IMC by submission of an Unanticipated Problem Report via secure email to NCCIH and fax or email to the IMC chair.

19.4 Safety Outcomes

See DSMP for specifics.

19.5 Descriptive Analysis

The safety outcomes will be summarized by type and by intervention in terms of frequency of the event in each individual, number of participants having the event, timing relative to randomization, and relatedness to the study treatment (definitely, probably, possibly, definitely not). As acupuncture is a relatively safe treatment, we anticipate incidence of possibly treatment related hospitalizations and deaths to be relatively rare so do not propose any formal statistical tests of the SAE data.

20. Interim Analyses

There are no planned interim analyses of primary or secondary outcome data before the study is completed. However, if in context of evaluating the safety outcomes the DSMB requests interim effectiveness estimates they will be provided. No formal futility or effectiveness interim analyses will be conducted.

21. References

1. Hornbrook M, Goodman, M.J., editor. Adjusting health benefit contributions to reflect risks. Greenwich: JAI Press; 1991.
2. O'Keeffe-Rosetti MC, Hornbrook MC, Fishman PA, Ritzwoller DP, Keast EM, Staab J, Lafata JE, Salloum R. A standardized relative resource cost model for medical care: application to cancer control programs. Journal of the National Cancer Institute Monographs. 2013;2013:106-16. Epub 2013/08/22. PMCID: PMC3748000.
3. Pickard AS, Law EH, Jiang R, Pullenayegum E, Shaw JW, Xie F, Oppe M, Boye KS, Chapman RH, Gong CL, Balch A, Busschbach JJV. United States Valuation of EQ-5D-5L Health States Using an International Protocol. Value Health. 2019;22:931-41.
4. Kim DH, Schneeweiss S, Glynn RJ. Comparing Approaches to Measure Frailty in Medicare Data: Deficit-Accumulation Frailty Index Versus Phenotypic Frailty. J Gerontol A Biol Sci Med Sci. 2018;73:989-90. PMCID: PMC6279157.
5. Carey IM, Shah SM, Harris T, DeWilde S, Cook DG. A new simple primary care morbidity score predicted mortality and better explains between practice variations than the Charlson index. Journal of clinical epidemiology. 2013;66:436-44.
6. Mayhew M, DeBar LL, Deyo RA, Kerns RD, Goulet JL, Brandt CA, Von Korff M. Development and Assessment of a Crosswalk Between ICD-9-CM and ICD-10-CM to Identify Patients with Common Pain Conditions. The journal of pain : official journal of the American Pain Society. 2019. Epub 2019/05/28.
7. Cherkin DC, Sherman KJ, Avins AL, Erro JH, Ichikawa L, Barlow WE, Delaney K, Hawkes R, Hamilton L, Pressman A, Khalsa PS, Deyo RA. A randomized trial comparing acupuncture, simulated acupuncture, and usual care for chronic low back pain. Arch Intern Med. 2009;169:858-66. PMCID: PMC2832641.
8. Cherkin DC, Sherman KJ, Kahn J, Wellman R, Cook AJ, Johnson E, Erro J, Delaney K, Deyo RA. A comparison of the effects of 2 types of massage and usual care on chronic low back pain: a randomized, controlled trial. Ann Intern Med. 2011;155:1-9. PMCID: PMC3570565.
9. Sherman KJ, Cherkin DC, Erro J, Miglioretti DL, Deyo RA. Comparing yoga, exercise, and a self-care book for chronic low back pain: a randomized, controlled trial. Ann Intern Med. 2005;143:849-56.
10. Rundell SD, Sherman KJ, Heagerty PJ, Mock CN, Jarvik JG. The clinical course of pain and function in older adults with a new primary care visit for back pain. J Am Geriatr Soc. 2015;63:524-30.
11. Wang M, Fitzmaurice GM. A simple imputation method for longitudinal studies with non-ignorable non-responses. Biom J. 2006;48:302-18.

12. Liang KY, Zeger SL. Longitudinal data analysis using generalized linear models. Biometrika. 1986;73:13-22.
13. Weinstein MC, Russell LB, Gold MR, Siegel JE. Cost-effectiveness in health and medicine: Oxford university press; 1996.
14. Sanders GD, Neumann PJ, Basu A, Brock DW, Feeny D, Krahn M, Kuntz KM, Meltzer DO, Owens DK, Prosser LA. Recommendations for conduct, methodological practices, and reporting of cost-effectiveness analyses: second panel on cost-effectiveness in health and medicine. Jama. 2016;316:1093-103.
15. Drummond M, Sculpher M, Torrance G, O'Brien B, Stoddart GL. Methods for the economic evaluation of health care programmes. 3rd ed: Oxford University Press; 2005.
16. Manca A, Hawkins N, Sculpher MJ. Estimating mean QALYs in trial-based cost-effectiveness analysis: the importance of controlling for baseline utility. Health economics. 2005;14:487-96.
17. Neumann PJ, Cohen JT, Weinstein MC. Updating cost-effectiveness—the curious resilience of the $50,000-per-QALY threshold. N Engl J Med. 2014;371:796-7.
18. Barber JA, Thompson SG. Analysis of cost data in randomized trials: an application of the non-parametric bootstrap. Statistics in medicine. 2000;19:3219-36.
19. Efron B, Tibshirani R. An Introduction to the Bootstrap. Boca Raton, Fla. Chapman & Hall/CRC, Monographs on Statistics and Applied Probability; 1994.
