# Supplementary material for: Acupuncture for Chronic Low Back Pain in Older Adults: A Randomized Clinical Trial
Source: JAMA Netw Open. 2025 Sep 12;8(9):e2531348. doi: 10.1001/jamanetworkopen.2025.31348 (PMC12432643; doi:10.1001/jamanetworkopen.2025.31348)
Supplement: Supplement 3. — eAppendix 1. Missing Data eTable 1. Primary Outcome Follow-Up Rates and Patterns Overall and By Arm eTable 3. Baseline Variables Used in Process of Selecting Covariates Related to Missingness and to the 6-Month RDMQ eTable 4. Imputation Step 1: Summary of Fitted Imputation Model for Primary Analysis eTable 5. Imputation Step 2: Summary of Primary Estimation Model Fit to Imputed Data With Weighting eTable 6. Imputation Step 2: Summary of Estimation Model With Combined Acupuncture Groups With Weighting eTable 7. Summary of Logistic Weighting Model for Any Observed Follow-Up eTable 8. Summary of Estimated Weights eFigure. Histogram of Estimated Probabilities and Weights eTable 9. Summary of Step 2 Estimation Model Fit on Imputed Data Without Nonresponse Weights eAppendix 2. Sensitivity Analyses eTable 10. Primary Outcome Results Using Imputed Data Without Weights eTable 11. Complete Case Analysis of Primary Outcome Adjusting for Baseline Age, Sex, Race and Ethnicity, HCS, and RMDQ eTable 12. Complete Case Analysis of Primary Outcome Adjusting for Baseline Age, Sex, Race and Ethnicity, HCS, Education, RMDQ, PEG, Substance Use Disorder, Fear Avoidance, General Pain, and Pain Catastrophizing eTable 13. Complete Case Analysis of Primary Outcome Weighted for Nonresponse and Adjusting for Baseline Age, Sex, Race and Ethnicity, HCS, Education, RDMQ, PEG, Substance Use Disorder, Fear Avoidance, General Pain, and Pain Catastrophizing eAppendix 3. Secondary and Exploratory Analyses eTable 14. Adjusted Mean Changes from Baseline by Combined Acupuncture Arm and Adjusted Mean Differences in Change Between Combined Acupuncture Arm Versus UMC for the Primary Outcome (RMDQ) eTable 15. Moderator Analysis of the Effect of Acupuncture Versus Usual Medical Care (UMC) on Change in RMDQ at 6 Months From Baseline eTable 16. Moderator Analysis of the Effect of Acupuncture Versus Usual Medical Care (UMC) by Baseline RMDQ on 30% Reduction in RMDQ at 6 Months From Baseline eTable 17. Unadjusted [file jamanetwopen-e2531348-s003.pdf]

Supplemental Online Content

DeBar LL, Wellman RD, Justice M, et al. Acupuncture and chronic low back pain in older adults: a pragmatic randomized clinical trial. *JAMA Netw Open*. 2025;8(9):e2531348. doi:10.1001/jamanetworkopen.2025.31348

eAppendix 1. Missing Data
eTable 1. Primary Outcome Follow-Up Rates and Patterns Overall and By Arm
eTable 3. Baseline Variables Used in Process of Selecting Covariates Related to Missingness and to the 6-Month RDMQ
eTable 4. Imputation Step 1: Summary of Fitted Imputation Model for Primary Analysis
eTable 5. Imputation Step 2: Summary of Primary Estimation Model Fit to Imputed Data With Weighting
eTable 6. Imputation Step 2: Summary of Estimation Model With Combined Acupuncture Groups With Weighting
eTable 7. Summary of Logistic Weighting Model for Any Observed Follow-Up
eTable 8. Summary of Estimated Weights
eFigure. Histogram of Estimated Probabilities and Weights
eTable 9. Summary of Step 2 Estimation Model Fit on Imputed Data Without Nonresponse Weights
eAppendix 2. Sensitivity Analyses
eTable 10. Primary Outcome Results Using Imputed Data Without Weights
eTable 11. Complete Case Analysis of Primary Outcome Adjusting for Baseline Age, Sex, Race and Ethnicity, HCS, and RMDQ
eTable 12. Complete Case Analysis of Primary Outcome Adjusting for Baseline Age, Sex, Race and Ethnicity, HCS, Education, RMDQ, PEG, Substance Use Disorder, Fear Avoidance, General Pain, and Pain Catastrophizing
eTable 13. Complete Case Analysis of Primary Outcome Weighted for Nonresponse and Adjusting for Baseline Age, Sex, Race and Ethnicity, HCS, Education, RDMQ, PEG, Substance Use Disorder, Fear Avoidance, General Pain, and Pain Catastrophizing
eAppendix 3. Secondary and Exploratory Analyses
eTable 14. Adjusted Mean Changes from Baseline by Combined Acupuncture Arm and Adjusted Mean Differences in Change Between Combined Acupuncture Arm Versus UMC for the Primary Outcome (RMDQ)
eTable 15. Moderator Analysis of the Effect of Acupuncture Versus Usual Medical Care (UMC) on Change in RMDQ at 6 Months From Baseline
eTable 16. Moderator Analysis of the Effect of Acupuncture Versus Usual Medical Care (UMC) by Baseline RMDQ on 30% Reduction in RMDQ at 6 Months From Baseline
eTable 17. Unadjusted Percentage of Participants With 30% Improvement in RMDQ
eAppendix 4. Usual Medical Care Services and Fidelity
eTable 18. Health Care System Provided Health Insurance Covered/Partially Covered Pain-Related Usual Medical Care
eTable 19. Back Pain-Related Health Care Utilization by Study Group
eAppendix 5. STRICTA CONSORT Extensions (Standards for Reporting Interventions in Clinical Trials of Acupuncture)
eTable 20. Fidelity to STRICTA Checklist for Reporting Interventions in a Clinical Trial of Acupuncture
eReferences

This supplemental material has been provided by the authors to give readers additional information about their work.

© 2025 DeBar LL et al. *JAMA Network Open*.

eAppendix 1. Missing Data

The study met the pre-specified criteria enumerated in the statistical analysis plan for the primary analysis to use an imputation approach which assumes the data are missing not at random (MNAR). The specific criteria were missingness of the 6-month timepoint above 15% in any arm, or differential missingness of 10% or more between arms. As shown in **eTable 1**, the primary outcome was complete for 83.8% of those randomized to UMC and 93.6% and 92.2% for the SA and EA arms, respectively. Overall, 84.3% of individuals had all three time points observed and 57 individuals (7.1%) were missing follow-up at all three time points.

To estimate treatment effects for the primary analysis and minimize bias due to missing data, imputation of missing outcome data assuming MNAR was used in combination with regression adjustment and non-response weighting. Details regarding the imputation procedure, the models for imputation and for the weights, measured covariates, and variable selection are provided in the sections below. **eTable 2** (Supplement 4) displays the distributions of all measured baseline characteristics, both overall and within groups defined by three patterns of observed follow-up data for the primary outcome: 1) no recorded follow-up measures, 2) one or 2 follow-up measures recorded, or 3) all follow-up measures recorded.

Adjustment: The statistical analysis plan for the primary outcome pre-specified adjustment for age, sex, race, health care system site, and baseline RMDQ. Additional predictor variables were assessed and included in the imputation and estimation models if they were related both to missingness of the 6-month time-point and were predictive of the change in RMDQ between 6 months and baseline (primary outcome time point). Specific additional predictors for the imputation and estimation models were selected after assessing all other available covariates, one-by-one, in separate logistic regression models for missingness at 6-months, and in separate linear models for change in RDMQ at 6-months, while adjusting for study arm and the base set of pre-specified adjustment variables: age, sex, race, site and baseline RDMQ. In this context, if a covariate was significant at the 0.10 level in both the missingness and outcome models while adjusting for the base set of predictors, then it was added as an adjustment factor in the imputation and estimation models for the main analysis. A similar process was used to select additional covariates for the weight model, details of which are described below in the section on non-response weighting. **eTable 3** provides a list of variables that were assessed during the selection process and provides a list of additional variables that were selected for the main models and the weight model. **eTable 2** also provides p-values for variables that were significant in one or more of the selection models used to identify additional adjustment variables, i.e., variables that were significantly (P<0.10) related to the change from baseline in RDMQ at 6 months and/or the presence of missing values in the 6-month change from baseline in the RDMQ and/or the likelihood of missing all follow-up points (non-response) .

Imputation (MNAR): Implementation of the MNAR imputation approach for GEE[1] requires the specification and execution of a two-step modeling process. The first step is to impute missing outcome data using a pattern-mixture approach with reasonable choices for included missing patterns given the amount of missing data. To classify patterns of missing data in this study, among those with any observed follow-up measure we created a categorical variable indicating whether each participant had one (1.9%), two (6.8%) or three (84.3%) follow-up observations recorded. The full imputation model was specified as follows:

$$E(Y_{im}|X_i, \boldsymbol{Z}_i) = \alpha + \beta_{Acu} I(X_i = SA \text{ or } EA) + \boldsymbol{\beta}_z \boldsymbol{Z}_i + \beta_6 I(m=6) + \beta_{12} I(m=12)$$

$$+ \gamma_{SA,6} I(X_i = SA) I(m=6) + \gamma_{SA,12} I(\mathrm{X_i} = SA) I(m=12)$$

$$+\gamma_{EA,6} I(\mathrm{X_i} = EA) I(m=6) + \gamma_{EA,12} I(\mathrm{X_i} = EA) I(m=12)$$

$$+\eta_1 I(Obs_i = 1) + \eta_2 I(Obs_i = 2)$$

$$+\eta_{SA,1} I(X_\mathrm{i} = SA) I(Obs_i = 1) + \eta_{EA,1} I(X_\mathrm{i} = EA) I(Obs_i = 1)$$

$$+\eta_{SA,2} I(X_\mathrm{i} = SA) I(Obs_i = 2) + \eta_{EA,2} I(X_\mathrm{i} = EA) I(Obs_i = 2)$$

where Y_{im} is the change in RMDQ score from baseline for individual i at time point m, X_i is the intervention group, m is the number of months post randomization, and $\boldsymbol{Z}_i$ is the vector of baseline covariates. Further, Obs_i is a variable that indicates the pattern of observed outcomes present. In this case, Obs_i takes on the values 1, 2 or 3

© 2025 DeBar LL et al. *JAMA Network Open*.

depending on whether 1, 2 or 3 of the follow-up measures are observed for a randomized person. The $\boldsymbol{Z}_i$ include all adjustment variables as specified previously. We fit this model using GEE with independent working correlation and robust standard errors, accounting for clustering of participants within acupuncturists. The fitted imputation model was then used to replace missing outcomes data with predicted values from the model.

In the second step, the following estimation model was fit to the full imputed data using the following pre-specified functional form from the statistical analysis plan:

$$E(Y_{im}|X_i, \boldsymbol{Z}_i) = \alpha + \beta_{Acu} I(X_i = SA\ or\ EA) + \boldsymbol{\beta}_z \boldsymbol{Z}_i + \beta_6 I(m = 6) + \beta_{12} I(m = 12)$$

$$+ \gamma_{SA,6} I(\mathrm{X_i} = SA) I(m = 6) + \gamma_{SA,12} I(\mathrm{X_i} = SA) I(m = 12)$$

$$+ \gamma_{EA,6} I(\mathrm{X_i} = EA) I(m = 6) + \gamma_{EA,12} I(\mathrm{X_i} = EA) I(m = 12).$$

Parameter estimates are taken directly from the solution to the second step GEE model above and the variance-covariance matrix for the joint imputation and analysis model are calculated as described in Wang and Fitzmaurice[1]. Summaries of the fitted imputation and estimation models are provided in **eTable 4** and **eTable 5**.

Note: Per the pre-specified statistical analysis plan, because there was not a significant difference between the standard and enhanced acupuncture groups at the 6-month follow-up point, the primary analysis model was modified to reflect this change using a pooled acupuncture group (results **eTables 6 and 14**):

$$E(Y_{im}|X_i, \boldsymbol{Z}_i) = \alpha + \beta_{Acu} I(X_i = SA\ or\ EA) + \boldsymbol{\beta}_z \boldsymbol{Z}_i + \beta_6 I(m = 6) + \beta_{12} I(m = 12)$$

$$+ \gamma_{SA,6} I(\mathrm{X_i} = SA \text{ or } EA) I(m = 6) + \gamma_{SA,12} I(\mathrm{X_i} = SA \text{ or } EA) I(m = 12).$$

As a final note on imputation, there were a small number of cases where baseline covariates contained a limited number of missing values. To keep the entire randomized cohort intact for all analyses, these missing baseline values were imputed with their corresponding population means estimated from all available baseline data.[5]

Non-Response Weighting: The imputation approach assuming data are MNAR was used to impute missing data for those that had at least one observation, but we observed a higher-than-expected number of participants with all outcome time points missing (7.1%) and a differential by study arm: 13.5% for UMC, 3.4% for standard acupuncture, and 4.5% for enhanced acupuncture (**eTable 1**).

To be able to estimate an average treatment effect (ATE) amongst all randomized, and due to concern about differential missingness, we added non-response weighting to the pre-specified analysis. This was discussed with the funder and DSMB, but the statistical analysis plan was not modified. Therefore, for completeness, we include sensitivity analysis results without non-response weighting, and only imputation. **eTable 9** and **eTable10** provide model parameters estimates and summarized primary results, respectively, with non-response weighting removed. Compared to the primary analysis model with weights (**eTable 5**) there are some differences in model parameters estimates, but the estimated adjusted mean changes and adjusted pairwise mean differences in change are indistinguishable from the primary results in Table 2 of the manuscript. Correspondingly we believe the non-response weighted analysis is the best model to present as primary.

For the primary weighted analysis, to estimate the non-response weights, we fit a logistic regression model to the entire randomized population, with an indicator of at least one observed follow-up as the outcome. Predictor variables included study arm, age, sex, race, site, baseline RMDQ and additional variables that were associated both with the 6-month change from baseline in the RMDQ outcome and with having any follow-up at 3, 6 or 12 months (**eTable 2**). As detailed above for adjustment variables, additional variables for the weight model were evaluated one-at-a-time by fitting a sequence of separate regressions for 6-month change from baseline (linear) and for any follow-up (logistic). Those baseline covariates that, when included in models with study arm and the base set of adjustment variables, reached statistical significance at the 0.10 level in model for change and in the model for any follow-up (**eTable 3**) were added to the weight model.

The final model for the non-response weights took the following form:

$$logit(E(R_i|X_i, \boldsymbol{Z}_i)) = \beta_0 + \beta_{SA} I(X_i = SA) + \beta_{EA} I(X_i = EA) + \boldsymbol{\beta}_z \boldsymbol{Z}_i,$$

where R_i indicates having at least one observed follow-up measurement. Predictions, p_i, from this model are used to calculate weights $w_i = \frac{1}{p_i}$. The w_i were then scaled to the study sample size and included as weights in the GEE estimation model described in the imputation section (step 2). The fitted weight model is presented in **eTable 7,** and **eTable 8** and **eFigure** provide summaries of the predicted probabilities and corresponding weights.

eTable 1. Primary Outcome Follow-Up Rates and Patterns Overall and By Arm

	OVERALL		UMC		SA		EA	
	N	%	N	%	N	%	N	%
Randomized	800	100	266	100	265	100	269	100
3 months	720	90.0	221	83.1	247	93.2	252	93.7
6 months	719	89.9	223	83.8	248	93.6	248	92.2
12 months	706	88.3	220	82.7	246	92.8	240	89.2
At least 1 follow-up	743	92.9	230	86.5	256	96.6	257	95.5
No follow-up	57	7.1	36	13.5	9	3.4	12	4.5
One observed follow-up	15	1.9	3	1.1	5	1.9	7	2.6
Two observed follow-ups	54	6.8	20	7.5	17	6.4	17	6.3
Three observed follow-ups	674	84.3	207	77.8	234	6.3	233	86.6

eTable 3. Baseline Variables Used in Process of Selecting Covariates Related to Missingness and to the 6-Month RDMQ

**Category**	**Variables**
Variables included in all models	Age (65 to <75, 75-84, ≥85), sex, indicators of race/ethnicity (Black or African American, Hispanic, White, other (Asian, AI/AN, NH/PI, Multiple, unrecorded), indicators of site, baseline RMDQ, and indicator of study arms.
VARIABLES PROVIDED FOR SELECTION	
Demographic characteristics	Education (Any college vs less than college), household income (<$35K, $35 to <$150K, ≥$150K, unrecorded), employment (full-time, part-time, retired, unemployed), marital status (married/domestic partner vs not),
Pain Related & expectations	High impact chronic pain, received disability for pain, sciatica, multiple musculoskeletal conditions (EHR), number of musculoskeletal pain conditions (EHR), types of musculoskeletal pain conditions (EHR), pain has limited life in the past 3 months, expectations of acupuncture treatment, fear avoidance, pain catastrophizing.
Clinical Characteristics/Health Habits	BMI, Comorbidities (Elixhauser; EHR), frailty (EHR), diagnoses of depression (EHR) , diagnoses of anxiety (EHR), PHQ2, GAD2, presence of substance abuse disorder (EHR) and type (opioids, stimulants, sedatives, alcohol, cannabis, other; EHR), TAPS-1 screen (Tobacco, Alcohol, Prescription medication and other substance use), over the counter medications and self-help activities for lower back pain (self-management program for back pain, mind/body techniques, online information/classes, counseling), use of other substances for back pain (NSAIDS, acetaminophen, cannabis, CBD, herbal supplements), frequency and amount of physical, back and movement-based exercise.
Baseline secondary and Tertiary Outcomes	Pain intensity, PEG, PROMIS physical functioning, PROMIS fatigue, PROMIS sleep, PROMIS social engagement.
Baseline secondary and Tertiary Outcomes	Pain intensity, PEG, PROMIS physical functioning, PROMIS fatigue, PROMIS sleep, PROMIS social engagement.
FINAL VARIABLES SELECTED	
Additional variables selected for inclusion in imputation and estimation models	pain catastrophizing and the presence of general pain (EHR)
Additional variables selected for inclusion in non-response weight model.	Education (Any college vs. no college), active substance use disorder, presence of fear avoidance, baseline PEG score.

© 2025 DeBar LL et al. *JAMA Network Open*.

eTable 4. Imputation Step 1: Summary of Fitted Imputation Model for Primary Analysis

Step 1. Imputation Model	Overall	N			
Number of observations used	2145	715			
Num of missing values	84	28			
**Variable**	**Est.**	**SE**	**95% CI**		**p-value**
Intercept	2.34	0.65	1.07	3.62	0.0003
Treatment SA	-1.53	0.40	-2.31	-0.75	0.0001
Treatment EA	-1.42	0.47	-2.34	-0.49	0.0027
Time 6M	-0.12	0.26	-0.63	0.40	0.6561
Time 12M	0.20	0.32	-0.43	0.83	0.5396
Pattern 1 follow-up	1.35	0.98	-0.56	3.27	0.1663
Pattern 2 follow-ups	-1.48	1.21	-3.86	0.89	0.2204
SA*Time 6M	0.65	0.42	-0.18	1.47	0.1246
SA*Time 12M	0.44	0.46	-0.45	1.33	0.3359
EA*Time 6M	-0.27	0.35	-0.94	0.41	0.4401
EA*Time 12M	-0.44	0.47	-1.37	0.49	0.3565
SA*Pattern 1	-2.83	3.05	-8.80	3.15	0.3538
SA*Pattern 2	-0.95	1.96	-4.80	2.90	0.6276
EA*Pattern 1	-2.18	4.13	-10.27	5.90	0.5966
EA*Pattern 2	3.00	1.44	0.19	5.82	0.0366
Age 75 to <84	1.07	0.31	0.45	1.68	0.0007
Age >=85	2.24	0.57	1.12	3.37	<.0001
Sex Male	-0.56	0.26	-1.08	-0.05	0.033
Race Black/AfAm	1.28	0.43	0.43	2.13	0.0032
Race Hispanic	0.48	0.59	-0.68	1.64	0.4162
Race Other	0.09	0.68	-1.24	1.43	0.8899
Baseline RDMQ	-0.31	0.03	-0.38	-0.24	<.0001
Presence of general pain	1.22	0.33	0.58	1.86	0.0002
Presence of pain catastrophizing	0.06	0.03	0.00	0.13	0.0409

© 2025 DeBar LL et al. *JAMA Network Open*.

eTable 5. Imputation Step 2: Summary of Primary Estimation Model Fit to Imputed Data With Weighting

**Step 2. Estimation Model – Imputation with Weights**	**Overall**	**Persons**			
Number of Observations Used	2229	743			
Sum of Weights	2229				
**Variable**	**Estimate**	**SE**	**95% CI**		**p-value**
Intercept	2.26	0.64	1.00	3.52	0.000
Acupuncture	-1.41	0.36	-2.12	-0.71	<.0001
Time 6M	-0.07	0.26	-0.58	0.43	0.77
Time 12M	0.22	0.31	-0.39	0.83	0.48
SA*Time 6M	0.42	0.45	-0.46	1.30	0.35
SA*Time 12M	0.22	0.45	-0.67	1.11	0.63
EA*Time 6M	-0.12	0.33	-0.78	0.53	0.71
EA*Time 12M	-0.27	0.41	-1.08	0.54	0.51
Age 75 to <84	1.10	0.30	0.51	1.69	0.000
Age >=85	2.30	0.56	1.20	3.40	<.0001
Sex Male	-0.60	0.26	-1.12	-0.08	0.02
Race Black/AfAm	1.32	0.42	0.49	2.14	0.002
Race Hispanic	0.17	0.54	-0.89	1.24	0.75
Race Other	0.10	0.71	-1.28	1.49	0.88
Baseline RDMQ	-0.31	0.03	-0.38	-0.24	<.0001
Presence of general pain	1.18	0.32	0.55	1.80	0.000
Presence of pain catastrophizing	0.06	0.03	0.01	0.12	0.03

© 2025 DeBar LL et al. *JAMA Network Open*.

eTable 6. Imputation Step 2: Summary of Estimation Model With Combined Acupuncture Groups With Weighting

**Estimation Model-Combined Acupuncture Groups**	**Overall**	**Persons**			
Number of observations used	2229	743			
Sum of Weights	2229				
**Variable**	**Estimate**	**SE**	**95% CI**		**p-value**
Intercept	2.23	0.64	0.99	3.48	0.000
Acupuncture	-1.42	0.36	-2.12	-0.72	<.0001
Time 6M	-0.07	0.26	-0.58	0.43	0.772
Time 12M	0.22	0.31	-0.39	0.83	0.479
Acup*Time 6M	0.15	0.32	-0.47	0.77	0.641
Acup*Time 12M	-0.03	0.38	-0.78	0.73	0.945
Age 75 to <84	1.10	0.30	0.51	1.69	0.000
Age >=85	2.28	0.56	1.19	3.38	<.0001
Sex Male	-0.60	0.26	-1.11	-0.08	0.024
Race Black/AfAm	1.34	0.42	0.53	2.15	0.001
Race Hispanic	0.20	0.55	-0.87	1.27	0.710
Race Other	0.13	0.71	-1.26	1.53	0.851
Baseline RDMQ	-0.31	0.03	-0.38	-0.24	<.0001
Presence of general pain	1.17	0.31	0.55	1.78	0.000
Presence of pain catastrophizing	0.07	0.03	0.01	0.12	0.025

eTable 7. Summary of Logistic Weighting Model for Any Observed Follow-Up

**Variable**	**OR***	**95% CI**		**P-value**
SA	5.29	2.42	11.54	<0.001
EA	3.68	1.83	7.41	<0.001
Age 75 to <84	0.85	0.46	1.57	0.602
Age >=85	3.13	0.40	24.40	0.276
Sex Male	1.01	0.55	1.85	0.975
Race Black/AfAm	1.42	0.59	3.42	0.434
Race Hispanic	0.91	0.34	2.40	0.847
Race Other	1.01	0.35	2.89	0.989
Baseline RMDQ	1.04	0.97	1.13	0.285
Education Any College	1.85	0.89	3.85	0.099
Active SUD	0.27	0.08	0.93	0.037
Fear avoidance	0.63	0.31	1.29	0.205
Baseline PEG	0.73	0.60	0.90	0.003
c-statistic	0.77			
* OR = Odds Ratio				

© 2025 DeBar LL et al. *JAMA Network Open*.

eTable 8. Summary of Estimated Weights

	Sum	Mean	SD	Min.	P25	P50	P75	Max.
Estimated probability of having at least 1 follow-up.	--	0.93	0.07	0.44	0.92	0.96	0.98	1.00
Calculated weight used for analysis.	743	1.00	0.10	0.93	0.95	0.97	1.01	2.11

eFigure. Histogram of Estimated Probabilities and Weights

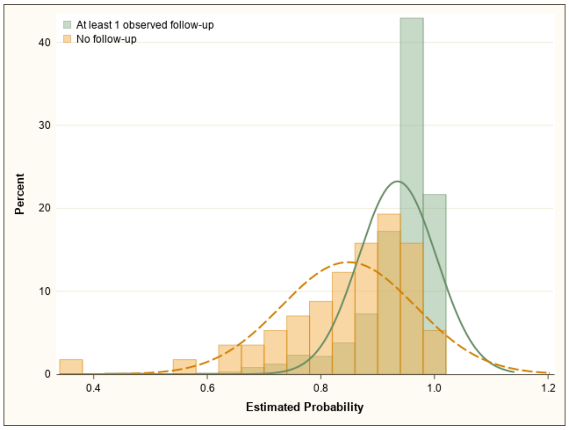

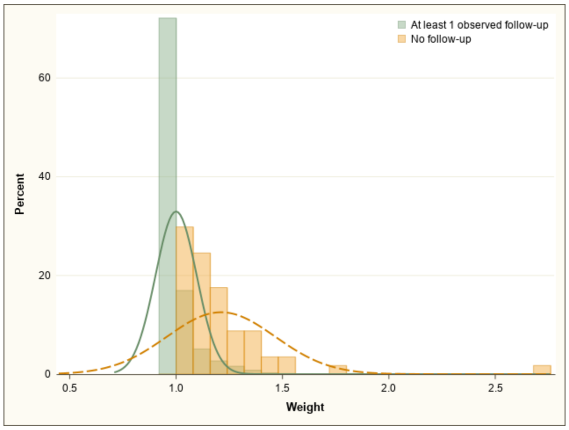

© 2025 DeBar LL et al. *JAMA Network Open*.

eTable 9. Summary of Step 2 Estimation Model Fit on Imputed Data Without Nonresponse Weights

**Step 2. Estimation Model - Imputation without Weights**	**Overall**	**Persons**			
Number of observations used	2229	743			
**Variable**	**Estimate**	**SE**	**95% CI**		**p-value**
Intercept	2.18	0.62	0.98	3.39	0.000
Acupuncture	-1.41	0.35	-2.10	-0.72	<.0001
Time 6M	-0.12	0.26	-0.62	0.38	0.647
Time 12M	0.20	0.31	-0.41	0.80	0.521
SA*Time 6M	0.46	0.45	-0.43	1.34	0.310
SA*Time 12M	0.25	0.45	-0.63	1.13	0.579
EA*Time 6M	-0.08	0.33	-0.73	0.57	0.811
EA*Time 12M	-0.25	0.41	-1.05	0.56	0.545
Age 75 to <84	1.12	0.30	0.53	1.71	0.000
Age >=85	2.34	0.56	1.25	3.44	<.0001
Sex Male	-0.60	0.26	-1.10	-0.10	0.019
Race Black/AfAm	1.33	0.42	0.51	2.15	0.001
Race Hispanic	0.25	0.53	-0.79	1.28	0.641
Race Other	0.18	0.68	-1.16	1.51	0.796
Baseline RDMQ	-0.31	0.03	-0.38	-0.24	<.0001
Presence of general pain	1.25	0.32	0.63	1.87	<.0001
Presence of pain catastrophizing	0.06	0.03	0.01	0.12	0.031

© 2025 DeBar LL et al. *JAMA Network Open*.

eAppendix 2. Sensitivity Analyses

Effect of Non-Response Weights: To understand the sensitivity of the effect estimates to the non-response weights, the estimation model was fit to the imputed data excluding the non-response weights. **eTable 9** provides a summary of the model and **eTable 10** shows the primary change and difference estimates. The difference between these results and the primary results including the non-response weights (main manuscript Table 2) are minimal.

eTable 10. Primary Outcome Results Using Imputed Data Without Weights

	Adj. Mean Changes from Baseline (95% CI)[b]			Omnibus	Adjusted Pairwise Differences (95% CI)[b]		
	UMC	SA	EA	P-Value[c]	SA vs UMC	EA vs UMC	EA vs SA
**Back-Related Dysfunction (RMDQ)**							
3 Months	-2.0 (-2.6, -1.5)	-3.5 (-3.9, -3.1)		>.001	-1.4 (-2.1, -0.7)		
6 Months[a]	-2.2 (-2.8, -1.5)	-3.1 (-3.7, -2.5)	-3.7 (-4.3, -3.0)	0.003	-1.0 (-1.8, -0.1)	-1.5 (-2.4, -0.6)	-0.5 (-1.5, 0.5)
12 Months	-1.9 (-2.5, -1.2)	-3 (-3.5, -2.5)	-3.5 (-4.2, -2.9)	0.001	-1.2 (-2.0, -0.3)	-1.7 (-2.6, -0.7)	-0.5 (-1.4, 0.4)

[a] Primary timepoint.
[b] Adjusted Mean change from baseline and adjusted mean differences were estimated by linear regression models on change from baseline using GEE with multiple outcomes per individual (3, 6 and 12 months), independent working correlation with sandwich standard errors incorporating correlation within person and acupuncturist, main effects for treatment group and follow-up time, interactions between treatment group and follow-up time, and adjusting for baseline age, sex, race, and clinical site, as well as additional variables related to both outcome and outcome missingness. As specified in the statistical analysis plan, missing data for individuals with at least 1 observed outcome were imputed using a single imputation method for data missing not at random when using GEE. Variables in imputation included treatment group, age, sex, race, and clinical site, as well as variables related to missingness for each outcome.
[c] To account for multiple comparisons pairwise inference only performed if omnibus p-value is significant. Bolding indicates non-significant omnibus p-value (p > 0.05).

© 2025 DeBar LL et al. *JAMA Network Open*.

Complete-Case Analyses: Sensitivity analysis results for the RMDQ outcome are presented in **eTables 11**, **12** and **13**. **eTable 11** provides results from a complete-case analysis (missing outcomes removed) adjusting only for age, sex, race, HCS and baseline RDMQ. The results in **eTable 12** augment the model with inclusion of variables related to missingness and to the six-month RMDQ outcome: PEG score, active SUD, general pain, fear avoidance and pain catastrophizing. **eTable 13** shows the analysis results when non-response weights are added to the model in **eTable 12**. The analyses below agree strongly with each other and with the final analytic results using imputation.

eTable 11. Complete Case Analysis of Primary Outcome Adjusting for Baseline Age, Sex, Race and Ethnicity, HCS, and RMDQ

	Adjusted Mean Changes from Baseline (95% CI)[b]			Omnibus P-Value[c]	Adjusted Pairwise Differences in Change (95% CI)[b]		
	UMC	SA	EA		SA vs UMC	EA vs UMC	EA vs SA
**Back-Related Dysfunction (RMDQ)**							
3 Months	-2.1 (-2.7, -1.5)	-3.4 (-3.8, -3.0)		<.0001	-1.3 (-2.1, -0.6)		
6 Months[a]	-2.2 (-2.8, -1.6)	-3.0 (-3.6, -2.5)	-3.7 (-4.3, -3.0)	.006	-0.8 (-1.6, 0.0)	-1.4 (-2.3, -0.5)	-0.6 (-1.5, 0.3)
12 Months	-1.9 (-2.5, -1.2)	-2.9 (-3.4, -2.4)	-3.6 (-4.2, -2.9)	.001	-1.0 (-1.9, -0.2)	-1.7 (-2.6, -0.8)	-0.6 (-1.5, 0.2)
**RDMQ, 30% Improvement**							
3 Months	31.2 (25.9, 37.6)	42.5 (38.3, 47.3)		.003	1.36 (1.11, 1.68)		
6 Months[a]	31.1 (25.9, 37.5)	39.3 (33.9, 45.6)	45.5 (39.6, 52.4)	.004	1.26 (1.00, 1.60)	1.46 (1.17, 1.83)	1.16 (0.93, 1.44)
12 Months	29.7 (24.5, 35.9)	36.9 (32.9, 41.4)	46.0 (41.2, 51.4)	<.0001	1.24 (1.00, 1.55)	1.55 (1.25, 1.92)	1.25 (1.06, 1.46)

[a] Primary timepoint.
[b] Adjusted Mean change from baseline and adjusted mean differences were estimated by linear regression models on change from baseline using GEE with multiple outcomes per individual (3, 6 and 12 months), independent working correlation with sandwich standard errors incorporating correlation within person and acupuncturist, main effects for treatment group and follow-up time, interactions between treatment group and follow-up time, and adjusting for baseline age, sex, race, health care system and baseline RMDQ. Adjusted mean percentage and relative risks were estimated by modified Poisson regression models using GEE with multiple outcomes per individual (3, 6 and 12 months), independent working correlation with sandwich standard errors incorporating correlation within person and acupuncturist, main effects for treatment group and follow-up time, interactions between treatment group and follow-up time, and adjusting for baseline age, sex, race, health care system, and baseline RMDQ.
[c] To account for multiple comparisons pairwise inference only performed if omnibus p-value is significant.

© 2025 DeBar LL et al. *JAMA Network Open*.

eTable 12. Complete Case Analysis of Primary Outcome Adjusting for Baseline Age, Sex, Race and Ethnicity, HCS, Education, RMDQ, PEG, Substance Use Disorder, Fear Avoidance, General Pain, and Pain Catastrophizing

	Adjusted Mean Changes from Baseline (95% CI)[b]			Omnibus P-Value[c]	Adjusted Pairwise Differences in Change (95% CI)[b]		
	UMC	SA	EA		SA vs UMC	EA vs UMC	EA vs SA
**Back-Related Dysfunction (RMDQ)**							
3 Months	-1.9 (-2.5, -1.3)	-3.4 (-3.8, -3.0)		<.0001	-1.5 (-2.2, -0.8)		
6 Months[a]	-2.1 (-2.7, -1.5)	-3.0 (-3.6, -2.5)	-3.6 (-4.3, -3.0)	.001	-1.0 (-1.8, -0.1)	-1.6 (-2.5, -0.7)	-0.6 (-1.6, 0.3)
12 Months	-1.7 (-2.4, -1.1)	-2.9 (-3.4, -2.4)	-3.5 (-4.2, -2.9)	<.0001	-1.2 (-2.0, -0.4)	-1.8 (-2.7, -0.9)	-0.7 (-1.5, 0.2)
**RDMQ, 30% Improvement**							
3 Months	29.0 (24.0, 35.0)	41.0 (37.3, 45.1)		<.0001	1.42 (1.16, 1.73)		
6 Months[a]	28.9 (24.0, 34.7)	37.9 (32.2, 44.5)	43.7 (38.1, 50.2)	<.0001	1.31 (1.04, 1.66)	1.52 (1.22, 1.89)	1.15 (0.91, 1.46)
12 Months	27.5 (22.7, 33.3)	35.5 (31.7, 39.9)	44.2 (39.3, 49.7)	<.0001	1.29 (1.04, 1.61)	1.61 (1.3, 1.99)	1.24 (1.05, 1.46)

[a] Primary timepoint.
[b] Adjusted Mean change from baseline and adjusted mean differences were estimated by linear regression models on change from baseline using GEE with multiple outcomes per individual (3, 6 and 12 months), independent working correlation with sandwich standard errors incorporating correlation within person and acupuncturist, main effects for treatment group and follow-up time, interactions between treatment group and follow-up time, and adjusting for baseline age, sex, race, health care system, baseline RMDQ, PEG, education, substance use disorder, fear avoidance, general pain and pain catastrophizing. Adjusted mean percentage and relative risks were estimated by modified Poisson regression models using GEE with multiple outcomes per individual (3, 6 and 12 months), independent working correlation with sandwich standard errors incorporating correlation within person and acupuncturist, main effects for treatment group and follow-up time, interactions between treatment group and follow-up time, and adjusting for baseline age, sex, race, health care system, baseline RMDQ, PEG, education, substance use disorder, fear avoidance, general pain and pain catastrophizing.
[c] To account for multiple comparisons pairwise inference only performed if omnibus p-value is significant.

© 2025 DeBar LL et al. *JAMA Network Open*.

eTable 13. Complete Case Analysis of Primary Outcome Weighted for Nonresponse and Adjusting for Baseline Age, Sex, Race and Ethnicity, HCS, Education, RDMQ, PEG, Substance Use Disorder, Fear Avoidance, General Pain, and Pain Catastrophizing

	Adjusted Mean Changes from Baseline (95% CI)[b]			Omnibus P-Value[c]	Adjusted Pairwise Differences in Change (95% CI)[b]		
	UMC	SA	EA		SA vs UMC	EA vs UMC	EA vs SA
**Back-Related Dysfunction (RMDQ)**							
3 Months	-2.0 (-2.6, -1.4)	-3.4 (-3.8, -3.0)		<.0001	-1.5 (-2.2, -0.7)		
6 Months[a]	-2.1 (-2.7, -1.5)	-3.0 (-3.6, -2.5)	-3.6 (-4.3, -3.0)	.001	-0.9 (-1.8, -0.1)	-1.6 (-2.4, -0.7)	-0.6 (-1.6, 0.3)
12 Months	-1.7 (-2.4, -1.1)	-2.9 (-3.4, -2.4)	-3.5 (-4.2, -2.9)	<.0001	-1.2 (-2.0, -0.3)	-1.8 (-2.7, -0.9)	-0.6 (-1.4, 0.2)
**RDMQ, 30% Improvement**							
3 Months	29.4 (24.2, 35.6)	41.1 (37.3, 45.2)		<.0001	1.40 (1.14, 1.72)		
6 Months[a]	28.7 (23.8, 34.6)	37.9 (32.2, 44.5)	43.8 (38.1, 50.3)	<.0001	1.32 (1.04, 1.68)	1.53 (1.22, 1.90)	1.16 (0.91, 1.47)
12 Months	27.5 (22.6, 33.4)	35.6 (31.7, 40.0)	44.3 (39.3, 49.9)	<.0001	1.30 (1.04, 1.62)	1.61 (1.30, 2.00)	1.24 (1.06, 1.47)

[a] Primary timepoint.
[b] Adjusted Mean change from baseline and adjusted mean differences were estimated by linear regression models on change from baseline using GEE with multiple outcomes per individual (3, 6 and 12 months), independent working correlation with sandwich standard errors incorporating correlation within person and acupuncturist, main effects for treatment group and follow-up time, interactions between treatment group and follow-up time, and adjusting for baseline age, sex, race, health care system, baseline RDMQ, PEG, education, substance use disorder, fear avoidance, general pain and pain catastrophizing. Additionally, non-response weights have been included to realign the estimates with the randomized population. Adjusted mean percentage and relative risks were estimated by modified Poisson regression models using GEE with multiple outcomes per individual (3, 6 and 12 months), independent working correlation with sandwich standard errors incorporating correlation within person and acupuncturist, main effects for treatment group and follow-up time, interactions between treatment group and follow-up time, and adjusting for baseline age, sex, race, health care system, baseline RDMQ, PEG, education, substance use disorder, fear avoidance, general pain and pain catastrophizing. Additionally, non-response weights have been included to realign the estimates with the randomized population.
[c] To account for multiple comparisons pairwise inference only performed if omnibus p-value is significant.

© 2025 DeBar LL et al. *JAMA Network Open*.

eAppendix 3. Secondary and Exploratory Analyses

Combining Acupuncture Arms for the Primary Outcome: As specified in the primary analysis plan if the two acupuncture groups were not statistically different and had a mean difference of 1.0 points or less on the RMDQ scale between groups we would combine acupuncture groups and re-run the primary analysis using the combined acupuncture arms. Results are shown in **eTable 14**.

eTable 14. Adjusted Mean Changes from Baseline by Combined Acupuncture Arm and Adjusted Mean Differences in Change Between Combined Acupuncture Arm Versus UMC for the Primary Outcome (RMDQ)

	Adjusted Mean Changes from Baseline (95% CI)[b]		Adjusted Mean Difference in Change (95% CI)[b]	P-Value	SMD[c]
	UMC	Acupuncture	Acu vs UMC		
**Back-Related Dysfunction (RMDQ)**					
3 Months	-2.0 (-2.6, -1.4)	-3.4 (-3.8, -3.0)	-1.4 (-2.1, -0.7)	<.0001	-0.32
6 Months[a]	-2.1 (-2.7, -1.5)	-3.4 (-3.7, -3.0)	-1.3 (-2.0, -0.5)	0.001	-0.27
12 Months	-1.8 (-2.5, -1.1)	-3.2 (-3.7, -2.8)	-1.4 (-2.2, -0.6)	<.0001	-0.27

[a] Primary timepoint.
[b] Adjusted Mean change from baseline and adjusted mean differences were estimated by linear regression models on change from baseline using GEE with multiple outcomes per individual (3, 6 and 12 months), independent working correlation with sandwich standard errors incorporating correlation within person and acupuncturist, main effects for combined acupuncture group and follow-up time, interactions between treatment group and follow-up time, and adjusting for baseline age, sex, race, and clinical site, as well as additional variables related to both outcome and outcome missingness. Missing data is handled as specified for the primary analysis.
[c] Standardized Mean Difference (SMD) is the adjusted mean change divided by the standard deviation of the change in outcome for that given time point.

© 2025 DeBar LL et al. *JAMA Network Open*.

Moderator Analyses: A series of moderator analyses were conducted as prespecified in the SAP (Supplementary Material 2). Sciatica was added post-hoc as an exploratory moderator following discussions with our DSMB. We conducted the moderator analysis using the combined acupuncture group relative to UMC for the 6-month primary time-point. We simplified the analyses by doing a complete case analysis amongst those with an observed 6-month outcome (N=719). For each moderator we applied a linear regression model fit using GEE with the moderator, acupuncture group indicator, and interaction between moderator and acupuncture group indicator. We further adjust for baseline RMDQ, race and ethnicity, age (75+), sex, Health Care System, BMI (overweight and obese categories), baseline PEG, education (college or higher), and general pain. For race and ethnicity subgroups a flexible cubic bspline for calendar time (df=5) was included to control for differences due to changes in recruitment over the course of the study. See **eTable 15** for the results showing no statistically significant moderators. An additional exploratory moderator analysis was added via the request of reviewer for this manuscript conducted for the binary outcome 30% reduction in RMDQ by baseline RMDQ following a similar modeling procedure as the continuous change in RMDQ outcome except modified Poisson regression was used instead of linear regression. The concern was the effect would be only amongst those with low RMDQs at baseline given 30% reduction is a smaller absolute change relative to those with higher RMDQs at baseline. There was no statistically significant moderation and further there was a larger estimated effect of the intervention amongst those with higher RMDQ at baseline (**eTable 16**)

eTable 15. Moderator Analysis of the Effect of Acupuncture Versus Usual Medical Care (UMC) on Change in RMDQ at 6 Months From Baseline

		Adjusted Mean Change from Baseline[a]		Adjusted Mean		
	N	UMC Est (95% CI)	Acupuncture Est (95% CI)	Difference Est (95% CI)	Moderator P-Value	Interaction P-Val
**POC vs Non-POC**						
POC	244	-2.5 (-3.9, -1.1)	-2.9 (-3.7, -2.1)	-0.4 (-1.8, 0.9)	0.528	0.131
Non-POC	465	-2.1 (-3.0, -1.2)	-3.8 (-4.6, -2.9)	-1.7 (-2.5, -0.8)	<0.001	
**Age**						
65-74	421	-2.4 (-3.2, -1.6)	-3.8 (-4.3, -3.3)	-1.4 (-2.3, -0.4)	0.005	0.524
75+	298	-1.8 (-2.7, -0.9)	-2.7 (-3.4, -2.0)	-0.9 (-2.0, 0.2)	0.108	
**Sex**						
Male	275	-3.3 (-4.6, -2.1)	-4.1 (-5.1, -3.2)	-0.8 (-1.9, 0.4)	0.197	0.362
Female	444	-2.3 (-3.3, -1.3)	-3.7 (-4.7, -2.8)	-1.4 (-2.3, -0.6)	0.001	
**Expectations of Acupuncture**						
Low/Medium (1-6)	361	-1.6 (-2.4, -0.7)	-3.1 (-3.5, -2.6)	-1.5 (-2.4, -0.6)	0.001	0.378
High(7-10)	334	-2.8 (-3.7, -1.8)	-3.7 (-4.3, -3.1)	-0.9 (-2.0, 0.2)	0.119	
**RMDQ at baseline**						
<18	557	-1.7 (-2.4, -1.0)	-2.6 (-3.1, -2.2)	-1.0 (-1.8, -0.2)	0.019	0.507
18+	162	-4.0 (-5.7, -2.2)	-5.6 (-6.6, -4.7)	-1.7 (-3.5, 0.2)	0.085	
**Sciatica (exploratory)**						
No	226	-2.4 (-3.3, -1.4)	-3.4 (-4.1, -2.7)	-1.0 (-2.2, 0.2)	0.100	0.533
Yes	486	-1.9 (-2.6, -1.2)	-3.4 (-3.9, -2.9)	-1.5 (-2.4, -0.6)	0.001	

POC: People of Color referring to those who responded with race and ethnicity as not white nor Hispanic

[a] Adjusted Mean Change and Difference from baseline are estimated with a linear regression model fit using GEE with indicator of acupuncture versus usual medical care, moderator, and interaction between moderator and acupuncture adjusting for baseline RMDQ, race and ethnicity, age (75+), sex, Health Care System, BMI (overweight and obese categories), baseline PEG, education (college or higher), and general pain. For race and ethnicity subgroups additionally adjusts for the bspline(calendar time, df=5) to control any differences over calendar since recruitment changed overtime.

[b] Moderator P-Value is the two-sided p-value comparing Acupuncture to UMC within the subgroup and Interaction P-Value is the two-sided p-value for the interaction term from the linear regression model.

*Included in this analysis are those with an observed outcome at 6 months (719; for the race and ethnicity(N=11) and expectations (N=24) moderators we further excluded those missing those variables). Missing adjusted covariates were imputed using mean imputation.

© 2025 DeBar LL et al. *JAMA Network Open*.

eTable 16. Moderator Analysis of the Effect of Acupuncture Versus Usual Medical Care (UMC) by Baseline RMDQ on 30% Reduction in RMDQ at 6 Months From Baseline

		Adjusted Percent MCID[a]				
		UMC	Acupuncture	Relative Risk	Moderator	Interaction
	N	Est(95% CI)	Est(95% CI)	RR(95% CI)	P-Value	P-Val
**RMDQ at baseline**						
<18	555	0.31(0.25, 0.38)	0.42(0.37, 0.47)	1.36(1.1, 1.67)	0.004	0.625
18+	162	0.28(0.15, 0.52)	0.45(0.35, 0.57)	1.61(0.83, 3.13)	0.157	

[a] Adjusted mean percentage and relative risks were estimated by modified Poisson regression models using GEE with multiple outcomes per individual (3, 6 and 12 months), independent working correlation with sandwich standard errors incorporating correlation within person and acupuncturist adjusting for baseline RMDQ, race and ethnicity, age (75+), sex, Health Care System, BMI (overweight and obese categories), baseline PEG, education (college or higher), and general pain.
[b] Moderator P-Value is the two-sided p-value comparing Acupuncture to UMC within the subgroup and Interaction P-Value is the two-sided p-value for the interaction term from the linear regression model.

Additional Unadjusted MCID RMDQ Outcome Data: For completeness we present raw unadjusted numbers with 30% improvement in RMDQ (**eTable 17)**.

eTable 17. Unadjusted Percentage of Participants With 30% Improvement in RMDQ

	Unadjusted Number with 30% improvement in RMDQ, n/N (%)		
	UMC	SA	EA
3 months[a]	67/217 (30.1)	109/247 (44.1)	112/251 (44.6)
6 months	78/222 (35.1)	100/247 (40.5)	121/247 (49.0)
12 months	70/219 (32.0)	96/244 (39.3)	116/238 (48.7)

© 2025 DeBar LL et al. *JAMA Network Open*.

eAppendix 4. Usual Medical Care Services and Fidelity

We present a description of usual medical care services for pain management that is covered or partially covered by health care service system (**eTable 18**) and summarize the utilization of back pain-related services 12 months pre and post randomization by study group (**eTable 19**).

eTable 18. Health Care System Provided Health Insurance Covered/Partially Covered Pain-Related Usual Medical Care

	**KPWA** (Integrated HCS)	**KPNC** (Integrated HCS)	**SH** (FFS HCS)	**IFH** (FQHC Network)
**Medication-related treatment**				
Opioids	Covered [a]	Covered [a]	Covered [a]	Covered
Gabapentin	Covered [a]	Covered [a]	Covered [a]	Covered
Duloxetine	Covered [a]	Covered [a]	Covered [a]	Covered
Other antidepressants	Covered [a]	Covered [a]	Covered [a]	Covered
Muscle relaxants	Covered [a]	Covered [a]	Covered [a]	Covered
Nonsteroidal anti-inflammatory drugs (NSAIDS)	Reduced Cost [c]	Reduced Cost [c]	Not covered [f]	Covered
Topical creams	Reduced Cost [c]	Reduced Cost [c]	Not covered [f]	Variable coverage
**Other Medical/device pain management strategies**				
TENS unit	Covered [a]	Covered [a]	Not covered [f]	Not covered
Injection therapy (e.g., ESI)	Covered [a]	Covered [a]	Covered [a]	Covered (limited)
Surgery	Covered [a]	Covered [a]	Covered [a]	Covered [b]
**Conventional nonpharmacological services**				
Physical therapy	Covered [a]	Covered [a]	Covered [a]	Covered [b]
Counseling / psychotherapy	Covered [a,e]	Covered [a,e]	Covered [a,e]	Covered [a]
**Other nonpharmacological services**				
Acupuncture	Covered [b]	Covered [a]	w/Supplemental insurance	Covered (very limited availability)
Chiropractic care	Covered [b]	Not covered [f]	w/Supplemental insurance	Not covered [f]
Massage therapy	Covered (limited)	Not covered [f]	Not covered [f]	Not covered [f]
Meditation/Mindfulness	Calm App only	Calm App only	Limited apps only	Not covered [f]
Tai Chi	Not covered [f]	Not covered [f]	Not covered [f]	Not covered [f]
Yoga	Not covered [f]	Not covered [f]	Not covered [f]	Not covered [f]

Abbreviations: KPWA – Kaiser Permanente Washington, KPNC – Kaiser Permanente Northern California, SH -Sutter Health, IFH – Institute of Family Health, HCS – healthcare system, FFS – fee for service, FQHC – Federally qualified healthcare clinic, TENS – Transcutaneous electrical nerve stimulation, ESI – epidural steroid injections

[a] generally available within healthcare system as part of healthcare insurance benefits (co-pays vary)

[b] generally available as part of healthcare insurance benefits but outside HCS clinics/facilities

[c] generally available within healthcare system at reduced cost to patients

[d] KPNC – also chronic-pain programs for patients with longstanding, difficult to manage cLBP, SH – includes chronic pain programs; KPWA – includes chronic pain specific psychotherapy service

[e] some patients have additional insurance riders that could cover this pain management treatment modality

[f] not covered by health insurance/healthcare system; available in community but payment largely out-of-pocket for patient; generally not feasible for patients with limited incomes receiving care in FQHCs

eTable 19. Back Pain-Related Health Care Utilization by Study Group

	Health services received during 12-month period prior to Randomization			Health services received during 12-month Intervention Period		
	**UMC Mean (SD, range)**	**SA Mean (SD, range)**	**EA Mean (SD, range)**	**UMC Mean (SD, range)**	**SA Mean (SD, range)**	**EA Mean (SD, range)**
Hospital inpatient stays	0.04 (0.2, 0-2)	0.05 (0.2, 0-1)	0.04 (0.2, 0-1)	0.03 (0.2, 0-2)	0.04 (0.2, 0-1)	0.02 (0.1, 0-1)
Hospital inpatient days	0.10 (0.6, 0-7)	0.14 (1.0, 0-14)	0.11 (0.6, 0-6)	0.16 (1.2, 0-14)	0.12 (0.9, 0-11)	0.05 (0.3, 0-3)
Hospital ambulatory visits	0.18 (0.5, 0-5)	0.28 (1.1, 0-13)	0.27 (0.7, 0-5)	0.19 (0.7, 0-6)	0.17 (0.6, 0-6)	0.12 (0.6, 0-4)
Hospital ambulatory surgical center visits	0.13 (0.6, 0-6)	0.29 (1.0, 0-7)	0.22 (0.7, 0-6)	0.11 (0.6, 0-7)	0.14 (0.6, 0-6)	0.11 (0.4, 0-2)
Outpatient clinic visits	2.72 (4.2, 0-28)	3.43 (5.9, 0-65)	3.13 (4.7, 0-42)	1.68 (3.6, 0-26)	1.87 (3.9, 0-31)	1.55 (3.5, 0-28)
Email, telephone and video visits	1.04 (1.8, 0-15)	1.19 (2.0, 0-10)	1.24 (1.9, 0-10)	0.50 (1.1, 0-8)	0.49 (1.2, 0-11)	0.37 (1.0, 0-6)
Pharmacy fills/refills	5.38 (8.4, 0-59)	4.25 (6.3, 0-58)	4.28 (5.6, 0-38)	5.32 (8.9, 0-63)	4.07 (6.0, 0-41)	3.81 (5.9, 0-42)

Note. Back-pain-related healthcare utilization was identified using back-pain-specific ICD-10 codes, medications commonly used to treat back pain (e.g., skeletal muscle relaxants, opioids), and back-specific procedures (e.g., injections, surgeries)
Only reflects data from three (Kaiser Permanente Washington, Kaiser Permanente Northern California, Sutter Health) of four clinical sites as information not available at IFH site where ambulatory care services only and limited ability to extract EHR data

© 2025 DeBar LL et al. *JAMA Network Open*.

eAppendix 5. STRICTA CONSORT Extensions (Standards for Reporting Interventions in Clinical Trials of Acupuncture)

eTable 20. Fidelity to STRICTA Checklist[10] for Reporting Interventions in a Clinical Trial of Acupuncture

Item	Description	BIA Acupuncture Intervention Protocol	Fidelity to BackInAction Acupuncture Intervention Protocol
1. Acupuncture rationale	(a) Style of acupuncture (e.g., TCM, Japanese, Korean, Western medical, Five Element, ear acupuncture, etc.)<br>(b) Reasoning for treatment provided, based on historical context, literature sources, and consensus methods, with references where appropriate<br><br>(c) Extent to which treatment was varied	Trial limited to acupuncture needling by funder for consistency with Center for Medicaid and Medicare Services (CMS) reimbursement criteria.[3] Details of needling options described in consensus intervention paper based on the literature and expert panel input and tracked on session forms.[4] Acupuncture needling follows principles of traditional acupuncture emphasizing local and distal needling, using acupoints on channels that traverse the back or are otherwise indicated for LBP and needling stimulation with intention to obtain 'de qi'. TCM patterns of disharmony were deemed to not be relevant to application of acupuncture needling for cLBP.[5,6] Intervention designed to be varied and individualized to participant presentation.	1(a)-(b). Study acupuncturists were oriented to the consensus acupuncture intervention and carefully recorded each session utilizing session forms and point options grid. Acupuncturists were able to select points not on the point option grid if they provided a rationale. (See 2. (b) below)<br><br>1(c). Re: treatment variation: the acupuncture intervention was designed to be varied and responsive to individual participants presentation withing the recommended framework reported below.
2. Details of needling	(a) Number of needle insertions per subject per session (mean and range where relevant)*<br><br>(b) Names (or location if no standard name) of points used (uni/bilateral)	(a) recommended range: 6-20 insertion sites; can be less with nervous or acupuncture naïve participant.<br><br>(b) **Points named in point grid on session form** organized by channel distinguishing local, distal, regional/torso, non-channel distal (includes those acupoints on the mid or upper back or on the front of the body as well as 'calming'/anxiety points (auricular/Shen Men, Yin tang).	2(a). Number of insertion sites/acupoints treated: Range **3-36**; Mean (SD): **15.3 (5.0)**<br><br>2(b). **76% of sessions used** both local and distal acupoints.<br>**23%** of sessions used additional **calming points** (Yin tang, auricular Shen men).<br>**20%** of sessions included additional regional/torsal area points.<br><br>**21%** of sessions incorporated points not included in the point grid with acupuncturist rationale provided.

© 2025 DeBar LL et al. *JAMA Network Open*.

Item	Description	BIA Acupuncture Intervention Protocol	Fidelity to BackInAction Acupuncture Intervention Protocol
	(c) Depth of insertion, based on a specified unit of measurement, or on a particular tissue level<br><br>(d) Response sought (e.g., de qi or muscle twitch response)<br><br>(e) Needle stimulation (e.g., manual, electrical)<br><br>(f) Needle retention time*<br><br>(g) Needle type (diameter, length, and manufacturer or material)	(c) Needle depth: **75% of safe needle depths** recommended per the literature.[7] Clarified in practitioner safety review training.<br><br>(d) Response sought: **'de qi' response**** recommended, based on practitioner discretion.<br><br>(e) Needle stimulation: **manual only**<br><br>(f) Needle retention time: 10-25 minutes recommended; 0-40 minutes allowed (allowing very brief to +15minutes needle retention time, per practitioner discretion and limitation of setting).<br><br>(g) Needle type: presterilized stainless-steel needles required; recommended **non-coated needles** for increased analgesic effect.[8] Size and gauge left to **practitioner discretion**; recommendations clarified in safety review.	2(c). Depth of insertion recommended at 75% safe needling depths. Needling depth was not tracked.<br><br>2(d). 'De qi'** intended response reported for No, Some or Most/all of insertion point:<br>No (**1%** of sessions)<br>Some (**61%** of sessions)<br>Most/all (**39%** of sessions)<br><br>2(e) Manual needle stimulation only.<br><br>2(f) Needle retention time; Mean needle retention time (SD): **25.5** minutes (**6.5)**; Range **10-45** minutes. **63%** within 2-25 minutes; recommended needle retention range: **97%** of sessions ≤ 40 minutes<br><br>2(g). Only pre-sterilized, pre-packaged, stainless-steel needles are allowed. Needles: **39%** of acupuncturists used coated needles; **73%** uncoated. Needle gauge, length and brand varied.
3. Treatment regimen	(a) Number of treatment sessions<br><br>(b) Duration of treatment sessions*<br><br>(c) Frequency of treatment sessions	(a) Number of sessions: up to 15 treatments allowed during first 12 weeks (8 sessions considered critical dose); additional 6 sessions in next 12 weeks for those randomized to enhanced (maintenance) acupuncture arm (4 considered critical dose).<br><br>(b) Session duration: 40–60 min<br><br>(c) Frequency of sessions: weekly sessions within session number limit, left to the discretion of the practitioner and participant. For example, 2 sessions a week if needed, or skipping weeks if needed. Eight	3(a). Acupuncture sessions over first 12 weeks (M=**10.6**, SD = **4.0**); **82%** with ≥ 8 sessions (critical dose). For those in enhanced acupuncture (EA) arm, maintenance sessions included M=**4.6**, (SD=**2.2)**; with 78% with ≥ 4 sessions (critical dose)<br><br>3(b). Duration of session: mean=**53.6 minutes**, (SD=**10.6)**; range: **21-105**.<br><br>3(c). For the standard acupuncture (SA) group; Mean time between visits = **7.3** days, (SD = **3.2** days), range: **3-51** days.

© 2025 DeBar LL et al. *JAMA Network Open*.

Item	Description	BIA Acupuncture Intervention Protocol	Fidelity to BackInAction Acupuncture Intervention Protocol
		sessions deemed minimum critical number of sessions in first 12 weeks, with four minimum number of sessions in maintenance period	For the maintenance acupuncture (EA) group; Mean time between visits = **13.5** days, (SD = **5.5** days), range of time between visits: **3-56** days.
4. Other components of treatment	(a) Details of other interventions administered to the acupuncture group (e.g., moxibustion, cupping, herbs, exercises, lifestyle advice)<br><br>(b) Setting and context of treatment, including instructions to practitioners, and information and explanations to patients	(a) trial limited to acupuncture needling only. Recommended steps of intervention: interview/conversation, palpation, selection of acupuncture points (body and ear if used), a range of number of points treated, a range of point retention times and a range of session times. No other interventions typical to acupuncture therapy are allowed.<br><br>(b). Kaiser and Sutter sites used acupuncturists practicing in their community/practice settings. IFH created dedicated clinical space at their FQHC primary care sites for community acupuncturists to provide care. Acupuncturists were trained in safety review, acupuncture intervention protocol[4], site-specific logistics and use of REDCap forms for submitting acupuncture treatment forms. Trainings could be accessed throughout the study. Acupuncturists completed training for the protection of Human Subjects for Research.	4(a). Other interventions were not included in this trial. Acupuncture treatment details described above.<br><br>Self-care recommendations (general) were given verbally at 3554/6926 **(51.3%)** of participant sessions, including:<br>**45%** Movement, activity, exercise–general<br>**11%** Nutrition / hydration<br>**8%** Meditative movement like (Qigong, Tai Chi)<br>**7%** Breathing guidance /awareness<br><br>4(b). For three of the four site's acupuncture was provided at the acupuncturist's community office setting; for the 4th site (IFH) care was provided at the FQHC primary care clinics.
5. Practitioner background	Description of participating acupuncturists (qualification or professional affiliation, years in acupuncture practice, other relevant experience)	Trial acupuncturists required to be state licensed and qualified to practice acupuncture in the state where care will be provided. At least 5 years clinical experience post licensing preferred with experience in treating older adults with cLBP having multi-morbidities. Exceptions for three years' experience per individual applicant permitted. Consistent with pragmatic trail objectives, study acupuncturists were	All acupuncturists were state licensed with current registration, NCCAOM certification, credentialed at each health system with at least at least 5 years post-licensing clinical experience and specific experience working treating older adults with multi-morbidities and cLBP. Exceptions for three years' experience per individual applicant was permitted.

© 2025 DeBar LL et al. *JAMA Network Open*.

Item	Description	BIA Acupuncture Intervention Protocol	Fidelity to BackInAction Acupuncture Intervention Protocol
		limited to those currently delivering such services in everyday practice settings.	
6. Control or comparator interventions	(a) Rationale for the control or comparator in the context of the research question, with sources that justify this choice<br><br>(b) Precise description of the control or comparator. If sham acupuncture or any other type of acupuncture-like control is used, provide details as for Items 1 to 3 above.	(a) Pragmatic trial design and protocol of acupuncture compared to usual care described in DeBar et al (2023)[9] designed to: (1) align acupuncture with key parameters of current Medicare coverage for acupuncture for older adults with cLBP while incorporating pragmatic features to best reflect treatment flexibility encouraged to meet needs of diverse population of older adults with cLBP (two acupuncture comparator conditions), and (2) compare to and allow receipt of routine healthcare services received by older adults with CLBP within participating healthcare systems for pain management (usual care comparator).<br><br>(b) comparator ‘usual medical care’ alone.	6(a). Reported in main text and in design paper.[9]<br><br>6(b). Comparator of usual medical care (UMC) confirmed and reported in main text.

Note: The STRICTA protocol checklist is designed to replace CONSORT 2010's item 5 when reporting an acupuncture trial.[10]
* Extreme outliers (1%-tile and 99%-tile) where removed due to likely data entry errors for acupuncture reporting number of needles, needle time retention, and duration of session. This removed <140 records out of 6926 sessions reported (varied depending on amount of missing data for a given variable).
** “De qi is a sensation of qi arriving at an acupuncture point as it is being needled. De Qi can feel like soreness, heaviness, an ache, or twitch/fasciculation. The practitioner may feel the needle ‘grab’, tug or resist.”[4]

© 2025 DeBar LL et al. *JAMA Network Open*.

eReferences

1) Wang M, Fitzmaurice GM. A simple imputation method for longitudinal studies with non-ignorable non-responses. Biom J. 2006 Apr;48(2):302-18. doi: 10.1002/bimj.200510188. PMID: 16708780.
2) White IR, Thompson SG. Adjusting for partially missing baseline measurements in randomized trials. Stat Med. 2005 Apr 15;24(7):993-1007. doi: 10.1002/sim.1981. PMID: 15570623.
3) Centers for Disease Control and Prevention. National coverage determination, acupuncture for chronic lower back pain (clbp) 30.3.3. 2020; https://www.cms.gov/Medicare-Coverage-Database/view/ncacal-decision-memo.aspx?proposed=N&NCAId=295. Accessed October 30, 2024.
4) Nielsen A, Ocker L, Majd I, et al. Acupuncture intervention protocol: Consensus process for a pragmatic randomized controlled trial of acupuncture for management of chronic low back pain in older adults, an nih heal initiative funded project. *Glob Adv Health Med.* 2021;10:2164956121100709
5) Sherman KJ, Cherkin DC, Hogeboom CJ. The diagnosis and treatment of patients with chronic low-back pain by traditional chinese medical acupuncturists. *J Altern Complement Med.* 2001;7(6):641-650
6) Sherman KJ, Hogeboom CJ, Cherkin DC. How traditional chinese medicine acupuncturists would diagnose and treat chronic low back pain: Results of a survey of licensed acupuncturists in washington state. *Complement Ther Med.* 2001;9(3):146-153
7) Chou PC, Chu HY, Lin JG. Safe needling depth of acupuncture points. J Altern Complement Med. 2011;17(3):199-206
8) Kwon S, Lee Y, Park HJ, Hahm DH. Coarse needle surface potentiates analgesic effect elicited by acupuncture with twirling manipulation in rats with nociceptive pain. BMC Complement Altern Med. 2017;17(1):1.PMC5209881
9) DeBar LL, Justice M, Avins AL, et al. Acupuncture for chronic low back pain in older adults: Design and protocol for the backinaction pragmatic clinical trial. *Contemp Clin Trials.* 2023;128:107166
10) MacPherson H, Altman DG, Hammerschlag R, et al. Revised standards for reporting interventions in clinical trials of acupuncture (stricta): Extending the consort statement. *J Evid Based Med.* 2010;3(3):140-155

© 2025 DeBar LL et al. *JAMA Network Open*.
