# Supplementary material for: Acupuncture for Chronic Low Back Pain in Older Adults: A Randomized Clinical Trial
Source: JAMA Netw Open. 2025 Sep 12;8(9):e2531348. doi: 10.1001/jamanetworkopen.2025.31348 (PMC12432643; doi:10.1001/jamanetworkopen.2025.31348)
Supplement: Supplement 5. — Data Sharing Statement [file jamanetwopen-e2531348-s005.pdf]

Data Sharing Statement

DeBar. Acupuncture for Chronic Low Back Pain in Older Adults. *JAMA Netw Open*. Published September 12, 2025. doi:10.1001/jamanetworkopen.2025.31348

Data
Additional Information: ClinicalTrials.gov Identifier: NCT04982315 Clinical trial registration date: July 29, 2021.
Data available: Yes
Data types: Deidentified participant data, Data dictionary
How to access data: NIMH Data Archive, https://nda.nih.gov (Collection C5670) for three of the sites' data. For the fourth site's data, contact the authors at Andrea.J.Cook@kp.org and Lynn.DeBar@kp.org
When available: With publication

Supporting Documents
Document types: None

Additional Information
Who can access the data: Individuals with IRB approval from their local, non-for-profit organization with a research question related to pain conditions.
Types of analyses: For a specified purpose
Mechanisms of data availability: After IRB approval
Any additional restrictions: N/A
